# A fine‐scale phylogenetic assessment of digenean trematodes in central Alberta reveals we have yet to uncover their total diversity

**DOI:** 10.1002/ece3.4939

**Published:** 2019-02-28

**Authors:** Michelle A. Gordy, Patrick C. Hanington

**Affiliations:** ^1^ School of Public Health University of Alberta Edmonton Alberta Canada

**Keywords:** Alberta, cercariae, digenea, diversity, phylogenetics, trematoda

## Abstract

Despite over 100 years of digenean trematode parasite species descriptions, from a wide diversity of vertebrate and invertebrate host species, our ability to recognize the diversity of trematode species within a single lake remains an incredible challenge. The most challenging aspect is the identification of species from larval stages derived from intermediate hosts, due to the disjointed data of adult worm morphological descriptions, from which species are named, and links to corresponding molecular identifiers in depauperate databases. Cryptic species also play a significant role in the challenge of linking trematode larvae to adults, species identifications, and estimating diversity. Herein, we utilize a large, longitudinal dataset of snail first‐intermediate host infection data from lakes in Alberta, Canada, to infer trematode larval diversity using molecular phylogenetics and snail host associations. From our assessments, we uncover a diversity of 79 larval trematode species among just five snail host species. Only 14 species were identified to a previously described species, while the other 65 species are either cryptic or otherwise unrepresented by mitochondrial genes in GenBank. This study currently represents the largest and most diverse singular molecular survey of trematode larval fauna composed of over one thousand mitochondrial sequences. Surprisingly, rarefaction analyses indicate we have yet to capture the complete diversity of trematodes from our sampling area.

## 1 INTRODUCTION

Trends in the ecology of pathogens are reliant upon an accurate identification of both pathogen and host species. However, the precise identification of endoparasites can be precarious, due to the lack of hard, morphological structures that arthropod ectoparasites have (Mathison & Pritt, 1997). Furthermore, larval and immature endoparasites often lack reproductive structures used to identify their adult counterparts. Both of these problems are common among helminths (Jensen & Bullard, 2018; Roeber, Jex, & Gasser, 1998; Schell, 2009). Additionally, the revelation of cryptic species is becoming more common, as molecular methods expose diversity not identifiable by traditional, morphological methods (Detwiler, Bos, & Minchella, 2016; Detwiler, Zajac, Minchella, & Belden, 2013; Georgieva, Selbach et al., 2001; Herrmann, Poulin, Keeney, & Blasco‐Costa, 1993a; Locke, McLaughlin, Dayanandan, & Marcogliese, 2010; Miura, Kuris, & Torchin, 2003; Nadler & León, 2016; Pérez‐Ponce de León & Poulin, 2010b). Finally, there is a lack of general survey data on parasites, causing gaps in our understanding of diversity and richness for defined geographical locations (Adlard, Miller, & Smit, 1998; Adlard & O'Donoghue, 2015; Mollaret et al., 2015). Taken together, plastic and cryptic morphology, with a lack of survey data, makes it more difficult to correctly identify a parasite sample from a new location.

Recent meta‐ and spatial analyses have shown that our understanding of parasite diversity is biased toward location, time, and parasite class, correlating with when and where taxonomists are most active during their careers, and it is argued that more taxonomists are needed (Poulin, 2011; Poulin & Jorge, 2018). Molecular methods have come a long way in allowing faster and more precise species identifications and the ability to make hypotheses about species relationships and evolution considering cryptic morphology. However, even with these methods, regional checklists of host–parasite relationships remain incomplete (Poulin, Besson, Morin, & Randhawa, 2015). One major issue is depauperate and biased databases, directly related to research and funding interests, expertise, and the natural evolution of improving methodologies over time. So, not only do we need more taxonomists, but we need them to study more broadly to fill in these gaps in our understanding of parasite diversity.

Ecologically speaking, most parasites have incomplete life cycle descriptions. Likewise, our understanding of their distributions and interactions within and among host species is limited due to a lack of surveillance records and repeated or long‐term studies. The dispersion of parasite data constrains our knowledge of the finer details of their ecology across broad geographic ranges. Additionally, unreliable morphological assessments in survey data present the caveats that (a) the species identities may not be accurate and (b) the survey may not represent true diversity within the area, missing cryptic species all together and underestimating overall diversity. Furthermore, the onset of molecular methods for species identifications has widened the knowledge gap through revelations of prior undetected diversity that cannot always be traced to a described species. In fact, the revelation of cryptic species is enhanced with greater sequencing effort, and more so for trematodes than any other group of parasitic helminth (Pérez‐Ponce de León & Poulin, 2010b). This, overall, can make it incredibly difficult to understand the larger picture when it comes to parasite ecology because we are lacking long‐term, field studies, and precision in data collection.

Digenean trematodes are a very large group of parasitic helminths, with complex life cycles. The adult worms infect vertebrate hosts, in which their eggs are passed into the environment with the feces of the animal. The eggs hatch and infect a snail (or other mollusk), in which their larval development occurs. Larvae will emerge from their obligate, snail, first‐intermediate host to then infect either a second‐intermediate host or a definitive host, depending on the species. Current estimates for the number of trematode species range from 18,000 (Cribb et al., 2016) to 24,000 (Poulin & Morand, 2014).

Traditionally, taxonomic descriptions of trematodes are from morphological traits of adult worm stages derived from vertebrate hosts, as their most prominent features are fully developed and measurable, contrasting the less developed features of the larval stages (Schell, 2009). With the onset of molecular barcoding, not only have we realized the problems of cryptic morphology and the need for multiple lines of evidence for species delineation, but that for trematodes, we can now use larval stages to delineate species (Detwiler et al., 2016, 2013; Georgieva, Selbach et al., 2001; Gordy, Locke, Rawlings, Lapierre, & Hanington, 2013b; Locke, Mclaughlin et al., 2010; Schwelm, Soldánová, Vyhlídalová, Sures, & Selbach, 2011; Soldánová et al., 2014). This is advantageous in that it is considerably easier to collect larvae from snail, first‐intermediate hosts. The disadvantage is the lack of a direct connection between adult morphological records and molecular records.

The goal of this study was to capture an accurate identification of the trematode biodiversity among snail first‐intermediate hosts to establish a better, ecological understanding of trematode communities and how they differ geographically and change over time. In this study, we use molecular phylogenetic methods to assess species relationships, to identify collected specimens, and account for possible cryptic morphology. Snails and trematodes were collected from six lakes in central Alberta, Canada, over 3 years, from June to September. This longitudinal dataset provides novel contributions to the species diversity of trematodes, new geographical species records in central Alberta, and snail host association records, to better connect trematode life cycles. Though the data collected for this study were a continuation of our previous long‐term dataset (Gordy, Kish, Tarrabain, & Hanington, 2013a), the use of phylogenetic methods herein both expand and improve upon our understanding of trematode diversity and clarify identification issues we confronted previously.

Several trematode families have previously been given a considerable amount of attention in molecular phylogenies, more than others (e.g., Diplostomidae and Echinostomatidae). Therefore, species delimitation methods and acceptable sequence divergence limits have been tested for specific genes within these, well‐studied, trematode families (Blasco‐Costa & Locke, 2011; Detwiler et al., 2013; Georgieva et al., 2012; Georgieva, Selbach et al., 2001). Most trematode families have not been given such attention. Though there are general assumptions extrapolated from previous studies, such as 5% sequence divergence of cytochrome *c* oxidase subunit 1 (*cox1*) as an acceptable limit for species delimitation (Vilas, Criscione, & Blouin, 2014), this remains to be tested for all trematode families. Herein, we test this 5% assumption for delimitation using *cox1* across seven trematode families.

The resulting diversity estimates from this study exemplify both the power and utility of molecular phylogenetics for species identification, but this study also identifies gaps and caveats that trematode taxonomists may face in future studies. Therefore, we provide commentary on the current caveats of the field of trematode taxonomy, cryptic species, depauperate databases, and areas in need of further research. We also provide a current record of trematode and host associations within Alberta and encourage the continued effort to better understand trematode diversity from both a regional and global context.

## 2 METHODS

### 2.1 Trematode and snail sample collection and selection

**Figure 1:**
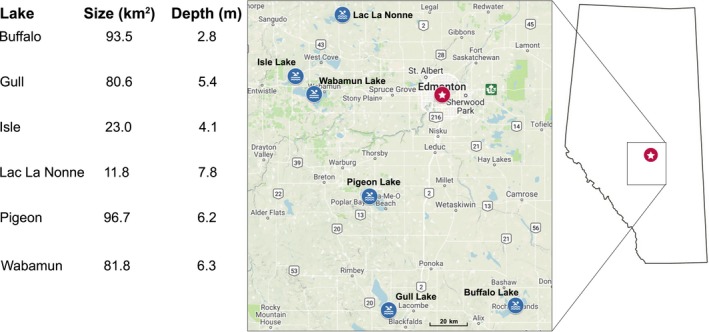
Sample collection locations. Map of the six lakes from which snails and trematodes were collected in central Alberta, Canada. Depth of lake is given as a mean depth in meters

As a continuation of the 2‐year survey described in Gordy et al. (2013a), snails were collected for an additional year in the same manner from the following sites: Lake Isle, Lake Wabamun, Gull Lake—Aspen Beach, and Buffalo Lake—Pelican Point, Rochon Sands, and The Narrows (Figure 1). All methods regarding collection and sample processing, including molecular methods, were the same as previously described (Gordy et al., 2013a, 2013b).

Briefly, snails were collected from sites previously established and brought back to the laboratory for examination of patent infection by larval trematodes. Trematode infections, when patent, resulted in larval cercariae emerging from the snail into the surrounding water. Free‐swimming cercariae were detected with a dissecting microscope, collected from the sample well, and preserved for downstream molecular work. Our original aim was to extract DNA and barcode every parasite sample. However, with over 2,400 samples, this goal was not feasible in cost and time. Nearly half the trematodes derived from the total collection were xiphidiocercariae, and previous sequencing efforts revealed these samples to be closest to *Plagiorchis *sp. (Gordy et al., 2013a). Therefore, much of the sequencing efforts went to all other morphotypes for which there were enough cercariae available for sequencing (i.e., >10 cercariae, to keep a voucher stored in ethanol). For cost feasibility, we chose to sequence only 70 haphazardly sampled xiphidiocercariae samples, representative of sites and snail host species from which they were found. The sequencing effort strategy for all other morphotypes was complete coverage.

### 2.2 DNA isolation, sequencing, and analysis

DNA was extracted from cercariae preserved in 50% RNAlater or 95% ethanol, as previously described (Gordy et al., 2013a). The partial NADH dehydrogenase subunit 1 (*nad1*) mitochondrial gene was sequenced for all cercariae for which morphological characterization or previous mitochondrial *cox1* sequencing attempts (Gordy et al., 2013a) placed them in the family Echinostomatidae. Because of high saturation within the *cox1* gene for this family, *nad1* has been the gene of choice in the literature (Detwiler et al., 2016, 2013; Georgieva et al., 2012; Morgan & Blair, 2015) and best represented the samples within GenBank for comparisons. For all other families, partial *cox1* was used (Gordy et al., 2013a, 2013b; Moszczynska, Locke, McLaughlin, Marcogliese, & Crease, 2016; Van Steenkiste, Locke, Castelin, Marcogliese, & Abbott, 2018). Nucleotide sequence inspection, trimming, alignments, model testing for best‐fit substitution models, and maximum‐likelihood (ML) and Bayesian inference (BI) phylogenetic analyses were as described in Gordy et al. (2013b). Model testing, utilizing BIC scores for determining best‐fit, was implemented in MEGA7 (Kumar, Stecher, & Tamura, 2012). All BI analyses were run in the MrBayes (Huelsenbeck & Ronquist, 2010) plugin with chain length 10,000,000, subsampling frequency 100,000, four heated chains (chain temp 0.2), and burn‐in length of 1,000,000. All ML analyses were run with the PhyML plugin (Guindon et al., 2018), estimating parameters, and with 1,000 bootstrap iterations. All molecular analyses were run using Geneious version 11 (http://www.geneious.com, Kearse et al., 2014).

Phylogenies were first constructed using a broad sampling of taxa within each family. Sequences of the same gene (either *cox1 *or *nad1*) were gathered from each species available in GenBank within that family. Because there are no standard methods yet employed for molecular taxonomic analysis within the Digenea, and much sequencing effort has been based on personal preference, we were unable to consistently attain a good representation of the species or even genera for several families, including Psilostomidae, Notocotylidae, and Plagiorchiidae. Because of issues with substitution saturation at broader taxonomic groupings for some families, their phylogenies were further refined into either genera or groups of closely related genera that were previously published as such (e.g., *Hypoderaeum* is paraphyletic to *Echinoparyphium* within the family Echinostomatidae (Detwiler et al., 2016; Kostadinova & Herniou, 2014)).

Phylogenies were constructed at a family‐level with nonredundant sequences to understand species relationships. These family‐based phylogenies were used as a benchmark for later phylogenies, in which redundant sequences were included for identification of individual sequences (specimen samples). Because there were many sequences, some phylogenies were divided to reduce the computation time (i.e., Strigeidae, Diplostomidae, and Echinostomatidae). We only present the information relevant to species identification phylogenies below, as the species relationships were the same as those within the nonredundant family‐level phylogenies.

### 2.3 Species delimitation

Trematode samples were first separated by gross morphology, evidenced by previously published larval trematode descriptions (Schell, 2009). Then, percent nucleotide identities by tBLASTn (Altschul, Gish, Miller, Myers, & Lipman, 1990), phylogenies from the literature where available (Blasco‐Costa, Poulin, & Presswell, 1977; Detwiler et al., 2016; Gordy et al., 2013b; Hernández‐Mena, García‐Varela, & Pérez‐Ponce de León, 2017; Locke, Mclaughlin et al., 2010), and species names given to the sequences in GenBank to which they most closely matched from BLAST results were used to group samples into trematode families and hypothesized genera.

After phylogenetic analyses, because many of our sequences were not directly within monophyletic groups of previously identified species, we employed additional tools to further distinguish taxa. The web app, Automatic Barcode Gap Discovery (ABGD; Puillandre, Lambert, Brouillet, & Achaz, 2005), was used in combination with a priori assumptions of a 5% cutoff in sequence divergence for species delimitation using *p* distances calculated in MEGA7 (Gordy et al., 2013b; Kumar et al., 2012). For ABGD, nucleotide alignments were inserted and tested using all three distance measurements (Jukes‐Cantor, Kimura 2.0, and simple distance) to look for agreements on grouping and prior maximal distance, using the default minimum slope of 1.5. Other specific methods will be described separately for each trematode family below. The one family that was included in downstream diversity analyses, but not described below is the Schistosomatidae because their phylogeny from this dataset was described and previously published (Gordy, Cobb, & Hanington, 2012).

#### 2.3.1 Family Notocotylidae

A final alignment of 98 *cox1 *sequences with a length of 327 bp was used for phylogenetic analyses. *Echinostoma hortense *(KR062182) was used as an out‐group because of its prior demonstrated phylogenetic relationship to the notocotylid *Ogmocotyle sikae *(KR006934.1; Liu et al., 2001), which was one of only two sequences from GenBank we were able to use for comparison. The *E. hortense *sequence did cause one small gap in the final alignment. Only *O. sikae *and *Notocotylus *sp. BOLD (KM538104) were used for comparison to the 95 sequences from this study, due to a lack of Notocotylid *cox1* sequences available with significant overlap. HKY + G was the best substitution model and was used for phylogenetic analyses.

#### 2.3.2 Family Psilostomidae

A *cox1* nucleotide alignment was made for 11 sequences, six from this study and five from GenBank, for a final length of 498 bp. *Echinochasmus japonicus* (NC_030518) was used as the out‐group for phylogenetic analyses because of its previously demonstrated relationship outside of Psilostomidae, but within the superfamily Echinostomatoidea (Tkach, Kudlai, & Kostadinova, 2015). Three other species were used for comparison, namely *Sphaeridiotrema globulus* (GQ890329), *S. pseudoglobulus* (GQ890328 & FJ477222), and *Pseudopsilostoma varium* (JX468064). HKY + G was the best‐supported nucleotide substitution model and was used for phylogenetic analyses. Because there were so few sequences, and therefore groups of species, ABGD was not utilized for confirmation.

#### 2.3.3 Family Haematoloechidae

A final nucleotide alignment consisted of seven sequences, one from *Plagiorchis *sp. (FJ477214) as the out‐group, two from GenBank (KM538096–KM538097: *Haematoloechus *sp. BOLD), and four from this study. The *Plagiorchis *sequence was used as the out‐group, based on previous use as such for phylogenies of Haematoloechidae sequences (Snyder & Tkach, 2015). The alignment was 469 bp with a few short gaps due to the out‐group sequence. HKY + G + I was the best‐supported nucleotide substitution model.

#### 2.3.4 Family Plagiorchiidae

A final nucleotide alignment of 56 *cox1* sequences was 437 bp in length. A sequence for *Haematoloechus *sp. (KM538096) was used as the out‐group (for reasons previously specified). Model test results showed the best nucleotide substitution model was HKY + I, which was utilized in BI and ML analyses.

#### 2.3.5 Family Echinostomatidae

Though *nad1 *was the primary gene of interest for this family, based on previous work, many samples from this study were first (or only) analyzed using *cox1* sequences. To resolve the issue of having some samples of one gene and some of another, sequencing of both genes for a few samples was done to make the link between gene trees. The only successful sequences from this attempt were from isolates MGC16B, MGC1214, and MGC1665. These sequences allowed the comparison between *nad1 *and *cox1 *phylogenies.

An alignment was made for all echinostome *cox1* sequences from this study along with those gathered from GenBank to represent as many species as available and that covered the same region of the gene. The final alignment included 113 sequences and was 391 bp long. Two sequences for *Euparyphium capitaneum* (KY636235–KY636236) were used as the out‐group (Tkach et al., 2015). From GenBank, sequences from the genera *Drepanocephalus, Hypoderaeum, *and *Echinostoma *were included in the alignment, as those were all that were available. Sequences included in the alignment from this study were from the genera *Echinoparyphium *and *Echinostoma*, and while there were no reference sequences within certain clades, there was overlap from the *nad1* gene tree to confirm the identity of these clades. The best‐fit model was GTR + G for both genes and for all genera within this family. The *nad1* phylogenies were split into multiple groups as discussed below.

##### *Drepanocephalus*

The final nucleotide alignment (*nad1*) was 390 bp and included two *Drepanocephalus auritus *(KP053262 and KP053263) sequences, one *Drepanocephalus *sp. (KP053264), two unknowns from the current study (MGC2147 and MGC2353), and a *Fasciola hepatica* (KT893744) sequence as the out‐group. Minor gaps were present between base pairs 180 and 190 where *F. hepatica* has a couple base pair differences. Because there were so few sequences, ABGD was not used for confirmation.

##### *Neopetasiger*

The final nucleotide alignment (*nad1*) of 21 sequences was 313 bp in length, and minor gaps occurred between base pairs 108 and 116 due to *F. hepatica *(KT893744), which was used as an out‐group for this alignment.

##### *Echinostoma*

A final nucleotide alignment (*nad1*) of 73 sequences was 386 bp long and included 31 unknown sequences from this study and all available species with significant overlap in the same region from GenBank. As in Soldánová et al. (2017), among others, *Isthmiophora melis *(AY168948) was used as an out‐group.

##### *Echinoparyphium/Hypoderaeum*

The final nucleotide alignment for *nad1* was 304 bp, with some minor gaps at position 81, 84, and 298, due to the out‐group, and included 262 sequences. Once again, *I. melis *was used as an out‐group. Both *Hypoderaeum* and *Echinoparyphium* sequences were included in this alignment, because previous phylogenies have shown them as paraphyletic (Detwiler et al., 2016).

#### 2.3.6 Superfamily Diplostomoidea

##### Family Diplostomidae

Based on recent phylogenies by Hernández‐Mena et al. (2017), and to reduce the overall size of the analysis, the Diplostomidae were divided into two groups for phylogenetic analyses utilized for identifications. Diplostomidae‐I included the genera *Austrodiplostomum*, *Tylodelphys*, and *Diplostomum* and resulted in a final nucleotide alignment of 197 sequences at 347 bp, using *Ornithodiplostomum scardinii* (KX931425) as out‐group. Diplostomidae‐II included the genera *Bolbophorus*, *Posthodiplostomum*, *Ornithodiplostomum*, *Neodiplostomum*, and *Alaria*, with a final alignment of 104 sequences at 317 bp and using *Crocodillicola pseudostoma* (MF398317–MF398318) as the out‐group. For both groups, GTR + G + I was the best substitution model and used for phylogenetic analyses.

##### Family Strigeidae

Like the Diplostomidae, the Strigeidae were divided into two groups for analyses and named after the ordering found in Hernández‐Mena et al. (2017). Strigeidae‐I included the genera *Cardiocephaloides*, *Cotylurus*, and *Ichthyocotylurus*. The final alignment was 356 bp long and included 152 sequences. *Tylodelphys scheuringi* (FJ477223) was used as the out‐group for phylogenetic analyses. Strigeidae‐II included genera from *Apatemon* and *Australapatemon*, with *Apharyngostrigea *spp. as the out‐group (HM064884–HM064885, JX977777, & JF769451). The final nucleotide alignment was 392 bp and included 313 sequences. The best nucleotide substitution model for the Strigeidae was HKY + G + I and used in all phylogenetic analyses.

### 2.4 Richness and recovery calculations

The following packages were utilized in R version 3.4.3 (R Core Team, 1997) to calculate richness and diversity metrics and plot them: *vegan *(Oksanen et al., 2010a) and *dplyr *(Wickham, François, Henry, & Müller, 2016). Species richness was derived using the diversityresult (*vegan*) command to add unique species by site as well as pooled species richness for all sites, by snail species, and to view how they were represented by lake. Species accumulation and rarefaction were analyzed using the specaccum (*vegan*) command, utilizing the “collector” method to derive site richness in the order the data were collected and the “rarefaction” method to view an individual‐based, rather than site‐based, method for species accumulation, respectively. An Arrhenius nonlinear model was fit to a species accumulation curve to view the species–area relationship utilizing the specaccum with “random” method and fitspecaccum (*vegan*) commands. If we assume that morphological identification of larval trematodes gives the greatest confidence at the taxonomic scale of family, we predict that accumulation curves will plateau faster than with information derived from molecular phylogenetic identifications that can provide confidence to the species‐level. To show this, we repeated the same accumulation and rarefaction analyses at the level of trematode family. This process was repeated for snail species, with exception of the Arrhenius nonlinear model, which would not converge.

## 3 RESULTS

A total of 17,447 snails were collected over the 3‐year period across all 11 sites (Figure 1 and Table 1). Snail species abundances are as follows: *Stagnicola elodes* = 9,505 (54.48%), *Lymnaea stagnalis* = 516 (2.96%), unidentified lymnaeid = 2,252 (12.91%), *Helisoma trivolvis* = 1,166 (6.68%), *Planorbula armigera* = 1 (0.01%), unidentified planorbid = 143 (0.82%), and *Physa gyrina* = 3,864 (22.15%). Of these collections, only 2,452 (14%) snails carried patent trematode infections, meaning cercariae were actively emerging from the snail. Unidentified lymnaeids and planorbids mentioned above were all uninfected. Most infections were found among *S. elodes *snails (1,892/77.16%), followed by *P. gyrina *(354/14.44%), *L. stagnalis* (123/5.02%), *H. trivolvis *(82/3.34%), and finally *P. armigera *(1/0.04%). Of these infections, 1,149 (46.8%) were classified as xiphidiocercariae by morphology (by having a clearly defined stylet in the anterior rim of the oral sucker (Schell, 2009)).

**Table 1:** Counts of snail species by collection site

	Buffalo Lake—Pelican Point	Buffalo Lake—Rochon Sands	Buffalo Lake—The Narrows	Gull Lake—Aspen Beach	Isle Lake	Lac La Nonne	Lac La Nonne site #2	Pigeon Lake—Provincial Park	Wabamun Lake—Provincial Park Beach	Grand total
*Helisoma trivolvis*	–	–	145	4	202	123	23	–	669	1,166
*Lymnaea stagnalis*	1	–	462	28	1	–	–	–	24	516
*Physa gyrina*	209	257	329	195	831	138	324	4	1,577	3,864
*Planorbula armigera*	–	–	–	–	–	–	–	–	1	1
*Stagnicola elodes*	3,567	368	36	399	3,457	1,179	370	–	129	9,505
Unidentified lymnaeid	–	–	–	1,192	–	–	–	–	1,060	2,252
Unidenitifed planorbid	–	–	78	7	1	–	–	–	57	143
Grand Total	3,777	625	1,050	1,825	4,492	1,440	717	4	3,517	17,447

A total of 1,091 trematode cercariae samples were successfully extracted and sequenced for downstream molecular phylogenetic analyses. Less than 200 cercariae samples, excluding xiphidiocercariae, were not included in the final diversity analyses, either because of low quantities of cercariae, low quantity or quality of DNA, or bad sequencing results. Phylogeny results will be discussed in the same order as above, by family, in the sections below.

Several new lineage and singletons have emerged from these phylogenies, and we refer to them below as “species.” We acknowledge the limitations of using molecular phylogenies for species identifications, without further supporting evidence (e.g., sequences from adult specimens) and that others would refer to them as operational taxonomic units (OTUs). However, we prefer to use the term species to remain consistent with our previous publications and sequence names.

### 3.1 Family Notocotylidae

Despite there being 19 different species represented in GenBank from the superfamily Pronocephaloidea, only five species had *cox1* sequences available at the time of this analysis, and one of those sequences was from a region other than the typical barcoding region (Folmer). Two of the sequences, Notocotylidae gen. sp. 1 NZ and sp. 2 NZ, were eventually removed from analyses because they did not align well. Therefore, the only sequences from GenBank left for phylogenetic comparisons with our sequences were *Ogmocotyle sikae *(mitochondrion, complete genome: NC_027112.1:6904–8460), *Notocotylus *sp. BOLD (KM538104), and Notocotylidae sp. MSB (KX670216).

#### 3.1.1 The former Gorgoderidae

From BLAST results, several sequences in our dataset matched most closely to the sequence for *Gorgoderina *sp. (FJ477202) in GenBank. When attempting to find other sequences for use in downstream analyses, we found that nearly every species in this family was only represented by 28S or ITS. A *cox1* sequence was available from *Pseudophyllodistomum macrobranchiola *(LC002523); however, the sequence was downstream of the Folmer region and did not overlap with our sequences. Upon further investigation, we found that these sequences matched very close to our other sequences for Notocotylidae sp., despite no BLAST matches from GenBank to Notocotylids. We therefore dissolved the Gorgoderidae sequence group, merging these sequences with the other Notocotylidae sequences, and have updated our previously published sequence (KT831348).

**Figure 2:**
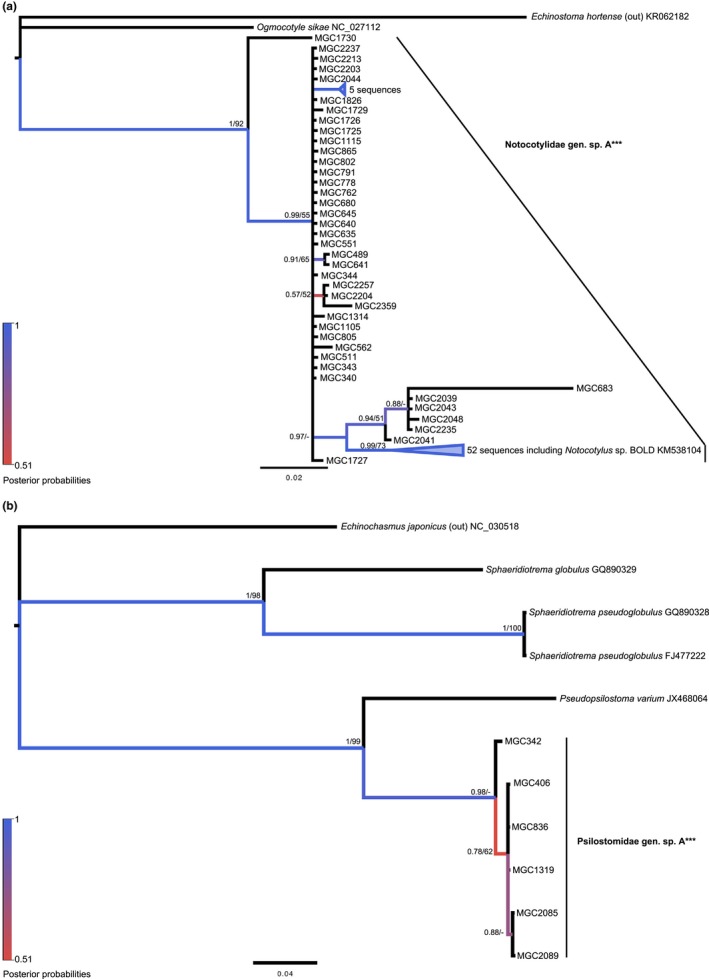
Molecular phylogeny of the Notocotylidae and Psilostomidae based on *cox1*. Bayesian inference phylogenies are given. Branches are colored by support values from phylogenetic analyses, with blue having the highest support. Posterior probabilities >0.50 and bootstrap values >50 are reported near the nodes, respectively. Accession numbers are given after species names. Emboldened taxa with three asterisks represent novel species from molecular analyses. (a) Notocotylidae. (b) Psilostomidae

Our phylogenetic analyses have revealed four Notocotylid species from our samples. Both ML and BI trees agreed on topology with strong statistical support (Figure 2a). Though all ABGD methods agreed, they only split the groups into three (JC *p*_max_ = 0.0215; K2 and simple *p*_max_ = 0.0129): *E. hortense*, *O. sikae,* and *Notocotylus *sp. The only GenBank sequence to group with our sequences was *Notocotylus *sp. BOLD (KM538104).

Based on pairwise distances, however, *Notocotylus *sp. as a single group determined by ABGD was not supported based on the 5% cutoff, as several sequences within the group were more than 5% different from others, despite the average intraspecific divergence being 3.0% for all. Two sequences, isolates MGC683 and MGC1730, expressed 6.8%–10.2% and 5.0%–10.2% intraspecific divergence, respectively. Without including these sequences, the range of intraspecific divergence was 0.0%–5.6%, which is more reasonable for a single lineage, however, still beyond the cutoff. We suspected further division within the tree topology, as some sequences continued to be closer or above the 5% divergence cutoff. Those that grouped outside of the primary clade (identified as *Notocotylus *sp. A) and closer to MGC683 were then separated further and support by intraspecific divergence was then within the cutoff range. In doing this, the average interspecific divergence between *Notocotylus *sp. A and D is 3.8% with a range of 2.8%–5.6% (Appendix: Table A1).

In considering the snail host species, *Notocotylus *sp. B (MGC1730) and C (MGC683), utilized *P. gyrina *and *H. trivolvis*, respectively, clearly supporting differentiation. However, the other isolates within *Notocotylus *sp. A and D used both *P. gyrina* and *S. elodes* as hosts, but curiously, *Notocotylus *sp. A was a primary *Physa* infecting species (36 *P. gyrina*/three *S. elodes*), while *Notocotylus *sp. D was a primary *Stagnicola *infecting species (49 *S. elodes*/five *P. gyrina*).

### 3.2 Family Psilostomidae

Only a few species within the Psilostomidae had representation by *cox1* in GenBank and significant overlap with our sequences. In molecular phylogenies, none of the sequences from this study grouped with any of the GenBank species representing the Psilostomidae family, but created their own monophyletic group, sister to *P. varium*. Both BI and ML trees agreed on topology (Figure 2b). The six sequences from this study were 0%–0.8% divergent from each other, with an average intraspecific divergence of 0.4%, and interspecific divergence of 14.3%–24.6% (Appendix: Table A2). Because of the low identity to any of the available genera from this family, the sequences from this study have therefore been identified broadly as Psilostomidae gen. sp. A. All six samples were derived from cercariae emerging from *H. trivolvis *snails.

### 3.3 Family Haematoloechidae

Despite there being 18 *Haematoloechus *spp. with *cox1* sequences available in GenBank at the time, only two sequences from this database overlapped with our sequences because of different choices in sequenced *cox1* regions. In addition, no other genera within the Haematoloechidae were currently represented in GenBank.

**Figure 3:**
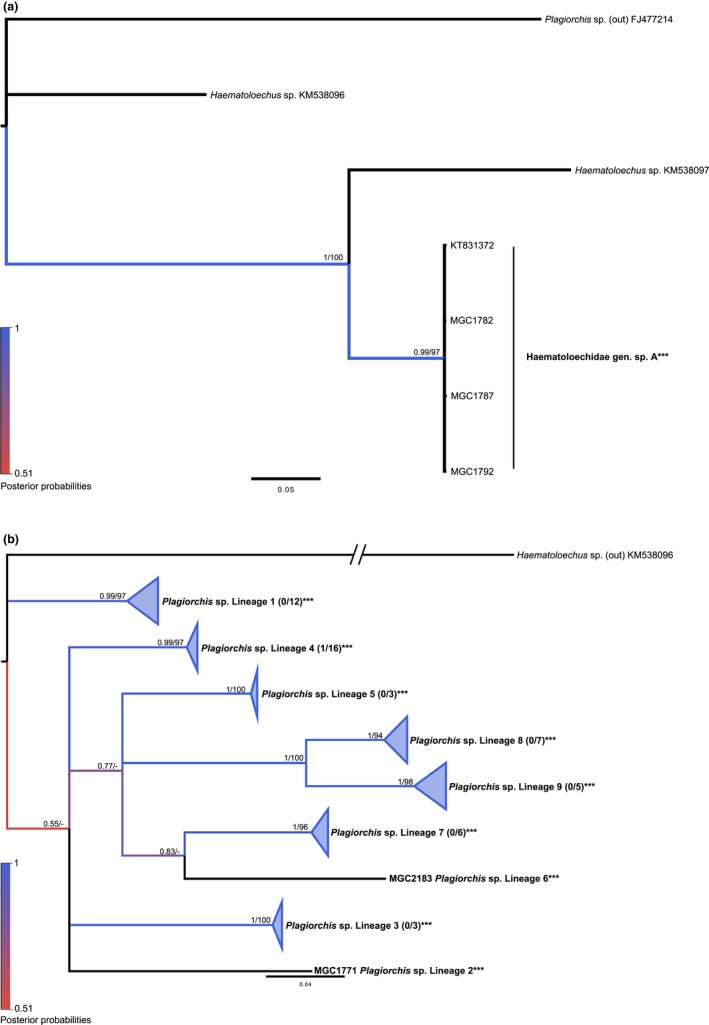
Molecular phylogeny of the Haematoloechidae and Plagiorchiidae based on *cox1*. Bayesian inference phylogenies are given. Clades representing a single species have been condensed for space. Branches are colored by support values from phylogenetic analyses, with blue having the highest support. Posterior probabilities >0.50 and bootstrap values >50 are reported near the nodes, respectively. Accession numbers are given after species names. Numbers in parentheses after taxon names correspond to the number of sequences within the clade. The first number is number of GenBank sequences and the second number, if given, represents number of sequences from this study. Emboldened taxa with three asterisks represent novel species from molecular analyses. (a) Haematoloechidae. (b) Plagiorchiidae

The four Haematoloechidae sequences from this study were 100% identical to each other, but 13.4%–25.8% divergent from GenBank sequences (Figure 3a and Appendix: Table A3). These four sequences were generalized to Haematoloechidae gen. sp. A, because there were no specific species within GenBank or other evidence that could provide more specificity at this time. Both BI and ML trees agreed with strong support for topology, as suspected for such little information. All four sequences were derived from samples that came from *S. elodes* snails collected at Pelican Point at Buffalo Lake.

### 3.4 Family Plagiorchiidae

Most Plagiorchiidae sequences in GenBank use a different region of the *cox1* gene, downstream from the Folmer region. The only sequence that aligned with ours was one *Plagiorchis *sp. (FJ477214). Phylogenetic analyses of Plagiorchiid sequences resulted in both ML and BI trees agreeing on topology with strong statistical support for external nodes and moderate support for internal nodes (Figure 3b). All methods within ABGD supported the differentiation of lineages within the tree to nine groups other than the out‐group (*p*_max (All)_ = 0.004–0.0599). Pairwise and averaged intraspecific divergence values were supported by the 5% cutoff, and the highest value was 2.1% within Lineage 1. The average interspecific divergence had a range from 8.9% to 18.8% (Appendix: Table A4). Further support for the differentiation of some lineages was found among intermediate host use, as Lineage 6 utilized *H. trivolvis*, Lineage 7 used *L. stagnalis*, and all other lineages were found emerging from *S. elodes*. Because this diversity was greater than we had expected by morphology (indicating possibly two species based on relative size) and prior BLAST results, we were unable to assign the unsequenced samples to these nine different lineages. Therefore, in downstream diversity analyses that require abundance information, these lineages have been conservatively lumped into one species, called *Plagiorchis *sp.

### 3.5 Family Echinostomatidae

For each separate alignment, ML and BI phylogenies were compared and found to agree on major topology. In instances where external node topology disagreed between the two methods, this was identified as a separate tree.

#### 3.5.1 *Drepanocephalus*

**Figure 4:**
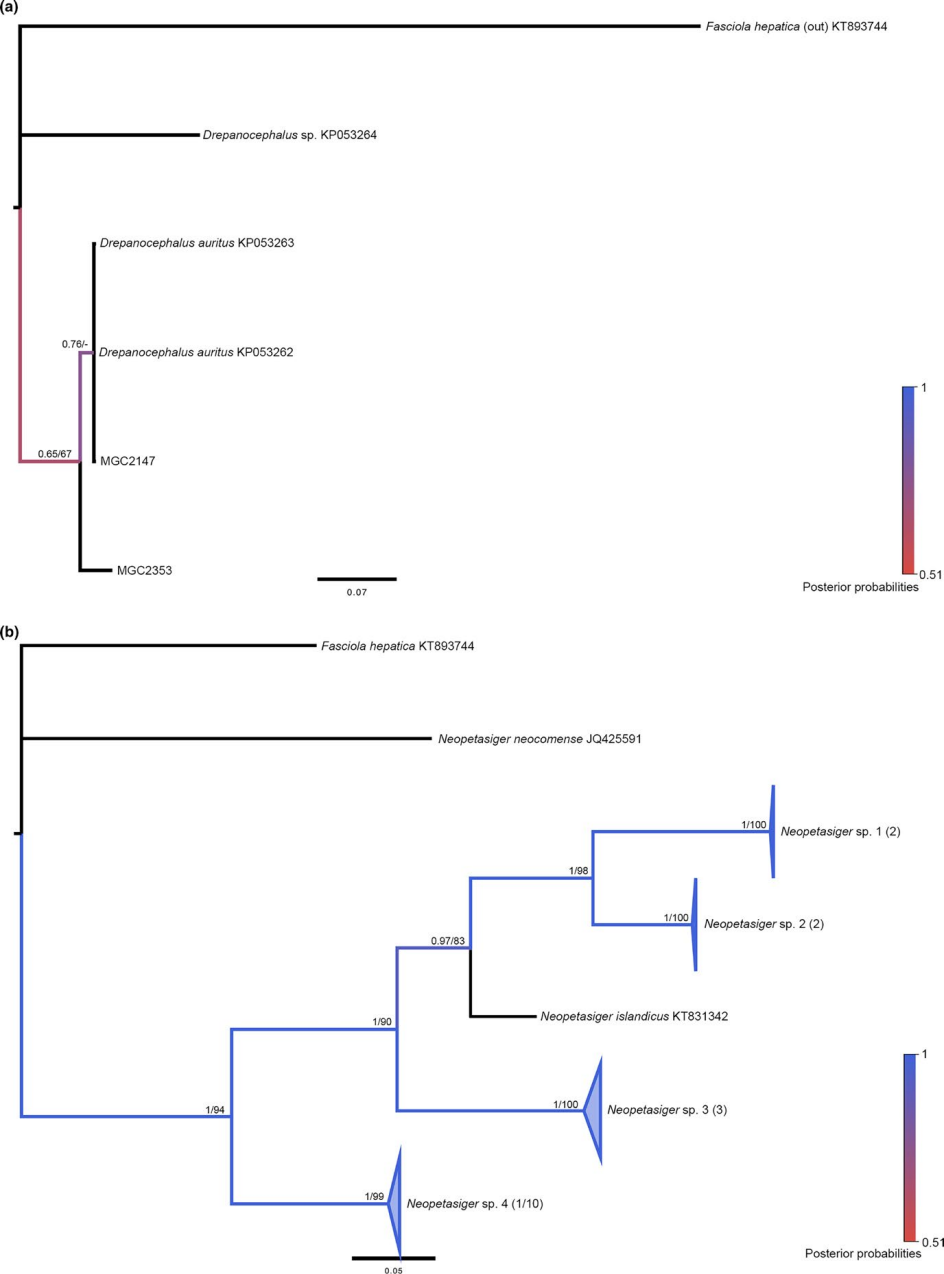
Molecular phylogeny of the Echinostomatidae: *Drepanocephalus* and *Neopetasiger* genera based on *nad1*. Bayesian inference phylogenies are given. Branches are colored by support values from phylogenetic analyses, with blue having the highest support. Posterior probabilities >0.50 and bootstrap values >50 from BI and ML analyses are reported near the nodes, respectively. Accession numbers are given after species names. Clades representing a single species have been condensed for space. Numbers in parentheses after taxon names correspond to the number of sequences within the clade. The first number is number of GenBank sequences and the second number, if given, represents number of sequences from this study. (a) *Drepanocephalus*. (b) *Neopetasiger*

Both *nad1* sequences from this study grouped monophyletically with *D. auritus* sequences. *Drepanocephalus *sp. was paraphyletic to the *D. auritus* group and displayed a nucleotide divergence range to the *D. auritus *group of 14.4%–15.5% (Figure 4a). The intraspecific divergence within the *auritus *group ranged from 0.0% to 4.4%, with an average of 2.2% (Appendix: Table A5). Both samples from this study came from *H. trivolvis* snails, which match with other records of specimens derived from planorbid snails in different geographical regions, specifically the United States and Brazil (Table 3). Recent work has revealed the synonymy of *D. auritus* with *Drepanocephalus spathans*, with *spathans *as the chosen name (Hernández‐Cruz, Hernández‐Orts, Sereno‐Uribe, Pérez‐Ponce de León, & García‐Varela, 2016). Therefore, we have identified our sequences according to this.

#### 3.5.2 *Neopetasiger*

The 10 sequences from this study all grouped within *Neopetasiger *sp. 4 and were <1% different in nucleotide identity from *Neopetasiger *sp. 4 (KM191817), with an average intraspecific divergence of 0.2% and an interspecific divergence of 21.1%–28.2% (Figure 4b and Appendix: Table A6). All *Neopetasiger *sp. samples from this study were derived from *H. trivolvis *snails, further indicating their specialization for planorbid snails, as indicated by other studies (Table 3).

#### 3.5.3 *Echinoparyphium/Hypoderaeum*

**Figure 5:**
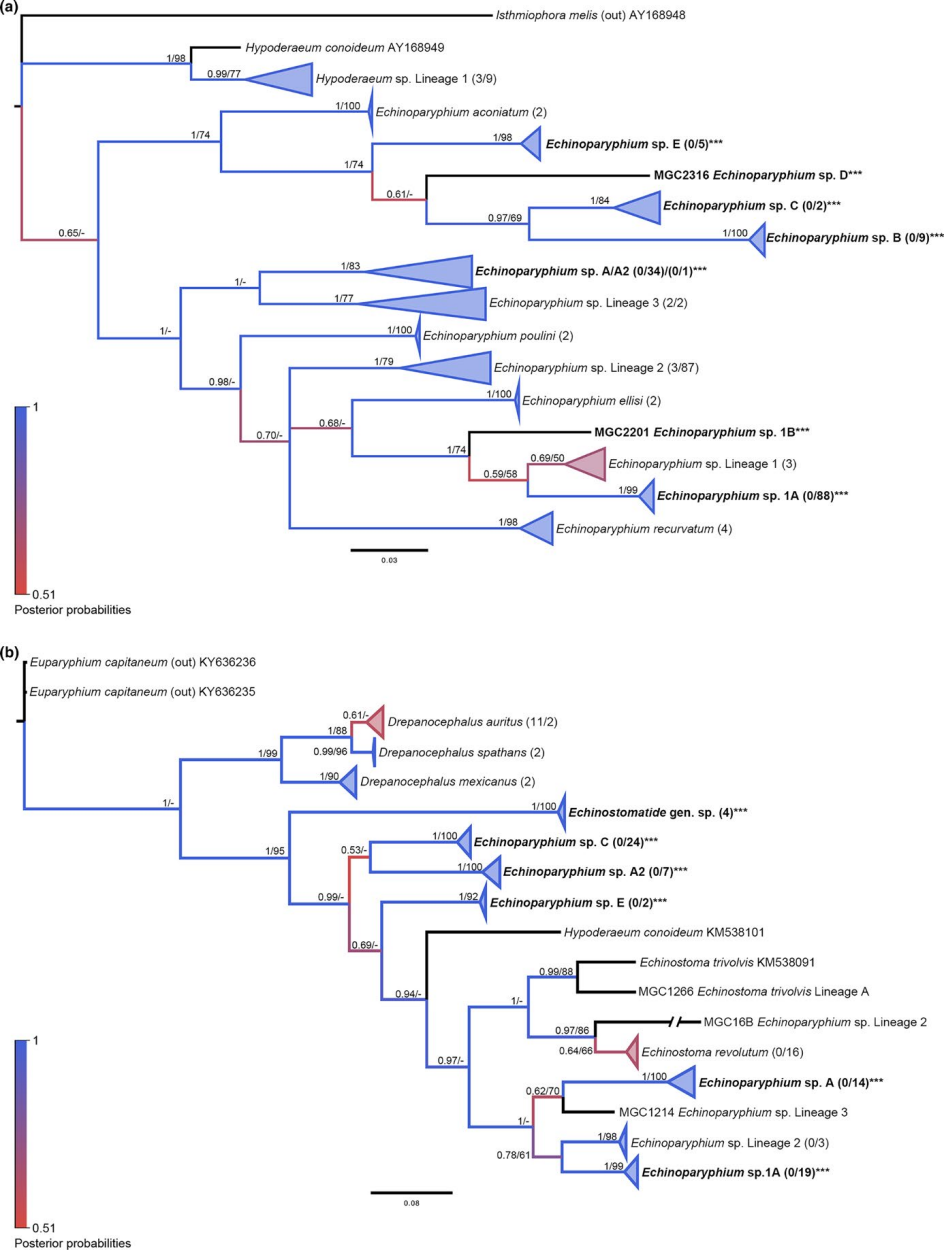
Molecular phylogeny of the Echinostomatidae: *Echinoparyphium/Hypoderaeum* genera based on *nad1* and *cox1*. Bayesian inference phylogenies are given. Branches are colored by support values from phylogenetic analyses, with blue having the highest support. Posterior probabilities >0.50 and bootstrap values >50 from BI and ML analyses are reported near the nodes, respectively. Accession numbers are given after species names. Clades representing a single species have been condensed for space. Numbers in parentheses after taxon names correspond to the number of sequences within the clade. The first number is number of GenBank sequences and the second number, if given, represents number of sequences from this study. Emboldened taxa with three asterisks represent novel species from molecular analysis. (a) *nad1*. (b) *cox1*

All methods in ABGD agreed on separation of the alignment into 17 groups (*p*_max (all)_ = 0.0129; Figure 5a). This separation was further supported by considering the range of intraspecific divergence values reported previously for several of these same lineages (Detwiler et al., 2016). Furthermore, most groups supported a clear separation of lineages by first‐intermediate host use, confirmed from both Indiana and Alberta. For most lineages, the average within‐group nucleotide divergence was <5%. Despite ABGD results, some lineages with >5% divergence, upon further inspection, revealed evidence for further splitting, including *Echinoparyphium *sp. Lineage 3 and *Hypoderaeum *sp. Lineage 1. For example, though ABGD showed *Echinoparyphium *sp. Lineage 3 to be one group made of four sequences, their *p *distance values were very divergent. The two sequences from GenBank previously identified as Lineage 3 were 2.7% divergent from each other, but 9.7%–11% divergent from the two sequence from our study that were 3.7% divergent from each other. To us, this was a clear split and was also highly supported by posterior probabilities and bootstrap values in phylogenetic analyses as well. We therefore derived a new lineage, *Echinoparyphium *sp. Lineage 4.

Within the *Hypoderaeum *sp. Lineage 1 clade, there was an obvious split occurring with three sequences forming their own clade (MGC577, MGC650, and MGC824). This split was not supported by ABGD or by host use, as all utilized *S. elodes* snails. The nucleotide divergence, however, ranged between 0.3% and 5.4%. The small clade that was found diverging from the rest was 0.3%–0.7% different from each other and 5.0%–5.4% different from the others. The split was obvious and well supported within the phylogenies. We have therefore split this lineage into two groups, now including *Hypoderaeum *sp. Lineage 2 (Figure 5a).

Several additional new lineages have been added to the genus *Echinoparyphium* because of our sequencing efforts. We have labeled these as *Echinoparyphium *sp. A–E*,* and for the two that are close to the previously identified *Echinoparyphium *sp. Lineage 1, we have labeled as *Echinoparyphium *sp. Lineage 1 A–B (Figure 5a).

One group we could not clearly delineate further, despite divergence higher than the cutoff. *Echinoparyphium *sp. Lineage 2 displayed above 5% intraspecific divergence, with an intraspecific range of 0.0%–5.7% and an average of 1.2%. The one isolate responsible for this greater divergence value is MGC369 that ranges from 1.7% to 5.7% from all other isolates within this lineage. All other isolates in this lineage have an intraspecific divergence range from 0.0% to 4.3% without MGC369. While this difference would seem a clear divergence, the phylogeny does not support the placement of MGC369 outside of this lineage. From host use, we find that MGC369 utilized *L. stagnalis*, whereas the majority of Lineage 2 isolates used *S. elodes*. While this would also seem to support differentiation, one other member MGC16B also utilized *L. stagnalis*, with very close sequence homology to other Lineage 2 members (0.3%–4.3%). Because neither the phylogenies nor host use supports further differentiation for this group, MGC369 remains in this lineage.

The *cox1* phylogenies for the Echinostomatidae (Figure 5b), for the most part, were well supported and matched patterns seen within the *nad1* phylogenies for this family. Because a few samples had both *cox1* and *nad1 *sequences available, the lineage identities were informed by *nad1 *because there were not many GenBank *cox1* sequences that matched. Overall, there was only one lineage within the *cox1 *phylogeny that had no overlapping sequences, and these have been labeled broadly as Echinostomatidae gen. sp.

There were two unexpected patterns found within the *cox1 *phylogeny as compared to the *nad1*. The lineage we identified as *Echinoparyphium *sp. Lineage 2 by *nad1* had a split, with very large divergence from isolate MGC16B to the other members of the lineage, upwards of 22.7%. Because there were no clear trends to help us understand this difference between the two genes, we have chosen to continue to include it within this same lineage, with the noted caveat.

The other unexpected pattern was within the lineage *Echinoparyphium *sp. A. Like the previous example, the lineage has split based on the *cox1 *gene. The range of pairwise distance within this group, including members of both split lineages, was 0.0%–21.4%, with an average intraspecific divergence of 10.5%. Without further evidence, one might conclude that this could be due to oversaturation in *cox1*, as previously noted for the echinostomes. We did see that one defining feature also separating these clades was intermediate host use. The clade that includes the isolate MGC1143 utilized *S. elodes* snails, whereas members of the clade with isolate MGC658 all used *P. gyrina* snails. By *nad1*, MGC1143 diverged from the other members of this clade by 1.0%–4.7%. MGC658 diverged by 0.3%–3.3%. Both could be considered within an acceptable range, leaving the decision of lump or split nearly impossible based on sequences alone. Host use, especially for the first‐intermediate snail host, is strong evidence that these are more likely to be two different species. In considering that these snails are members of different families and that the only other examples of different snail species being used within other genera of this family utilize species within the same snail family, namely, *S. elodes *or *L. stagnalis, *both members of the Lymnaeidae, our best judgment is to split this into two species, based on host use (Appendix: Tables A7 and A8).

#### 3.5.4 *Echinostoma*

**Figure 6:**
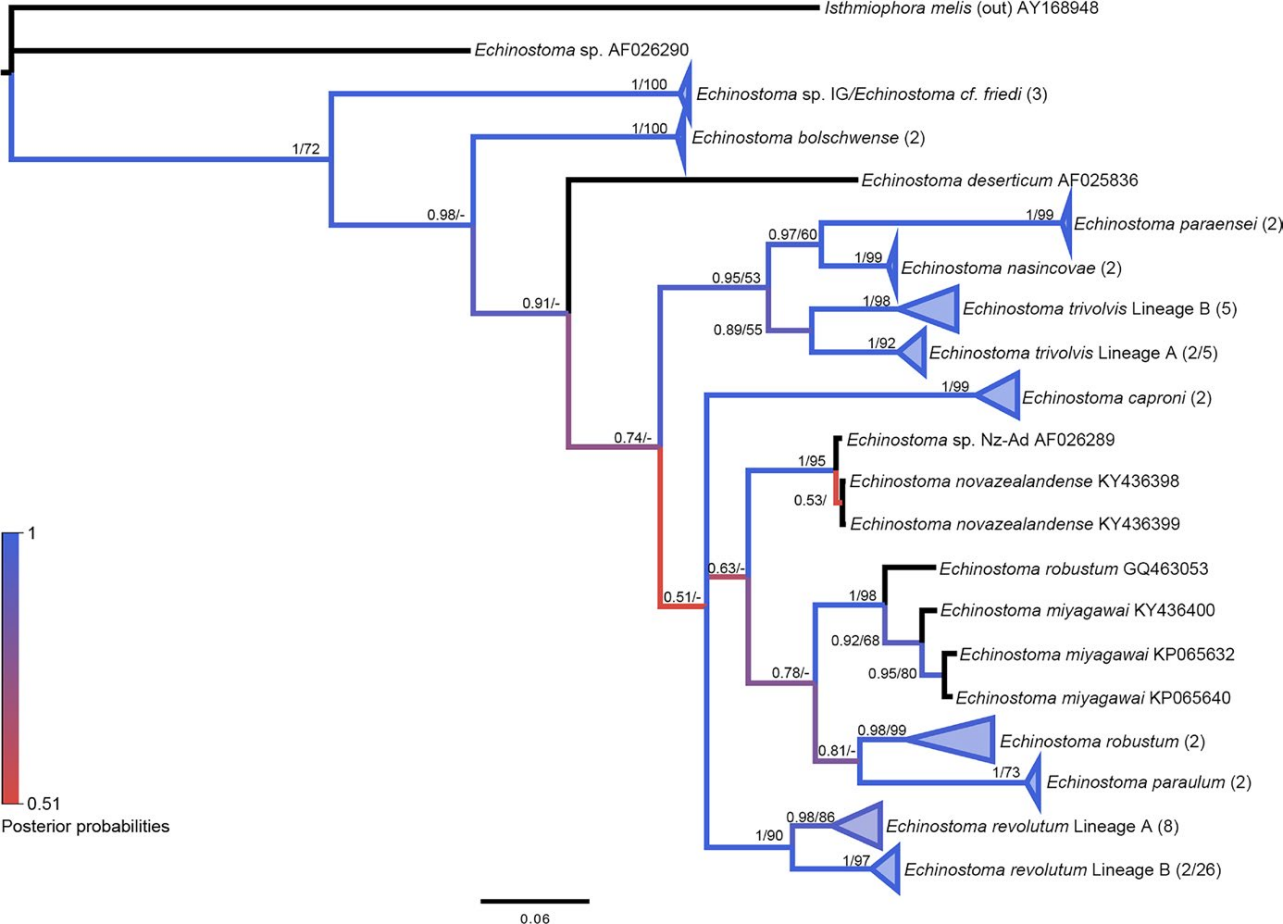
Molecular phylogeny of the Echinostomatidae based on *nad1*. Bayesian inference phylogenies are given. Branches are colored by support values from phylogenetic analyses, with blue having the highest support. Posterior probabilities >0.50 and bootstrap values >50 from BI and ML analyses are reported near the nodes, respectively. Accession numbers are given after species names. Clades representing a single species have been condensed for space. Numbers in parentheses after taxon names correspond to the number of sequences within the clade. The first number is number of GenBank sequences and the second number, if given, represents number of sequences from this study

There was strong nodal support by both BI and ML trees for the topology of the *Echinostoma* species (Figure 6). All ABGD distance methods supported the separation of the alignment into 15 groups (*p*_max_ (JC & K2) = 0.0359; *p*_max_ (simple dist.) = 0.02154). Intraspecific divergence values, based on the delineation cutoff, did not always support the same groups. For instance, *Echinostoma miyagawai, Echinostoma robustum, *and *Echinostoma revolutum* all exhibited ranges >5%, despite the average being lower, except for *E. robustum *whose average was 5.4%. Placement of one sequence within the tree did not match expectations but had high statistical support; *E. robustum *(GQ463053) grouped within a clade of *E. miyagawai*. The inclusion of the *robustum *sequence did explain the greater intraspecific divergence within this clade, but there was not support for its placement with the other *robustum* sequences that also exhibit high intraspecific divergence. Further inspection of this particular *robustum* sequence has shown previous assessments that have identified this same trend, indeed showing it to be *E. miyagawai* (Georgieva et al., 2012).

Sequences labeled/identified as *Echinostoma trivolvis *from GenBank resulted in two paraphyletic groups within the tree, the separation of which was confirmed by ABGD and within‐group divergence values of <5%. These observations confirmed previous lineage separation by Detwiler et al. (2016).

The *Echinostoma* sequences from the present study all fit within two clades, either *E. revolutum *or *E. trivolvis *Lineage A. The *revolutum *group exhibited higher than expected intraspecific divergence that ranged from 0.0% to 6.0%. Though not supported by ABGD, there did appear to be two separate groups emerging, one that has been found among *S. elodes *snails (Lineage B) and the other among *Lymnaea *spp. and ducks (Lineage A). By splitting these lineages, we saw more reasonable intraspecific divergence values within Lineage B (0.0%–1.6%), yet Lineage A continued to exhibit divergence higher than the cutoff (0.0%–5.7%; Appendix: Table A9). Because Lineage B isolates all utilized the same snail host, we were more confident in the grouping of this lineage, but believe that further sampling will likely show greater differentiation within Lineage A.

### 3.6 Family Diplostomidae

**Figure 7:**
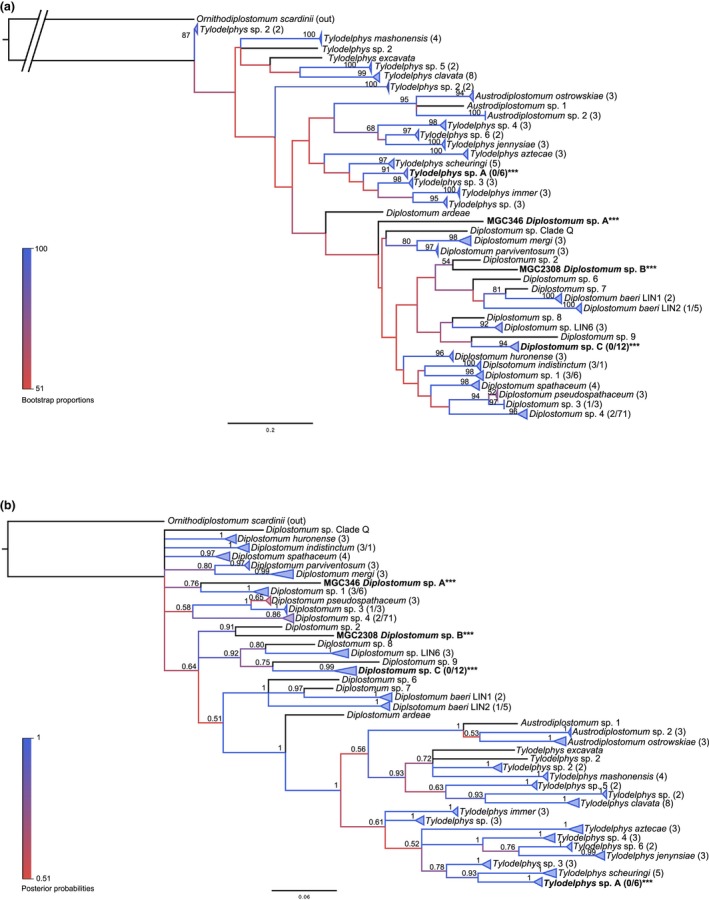
Molecular phylogenies of the Diplostomidae‐I Group based on *cox1*. Clades representing a single species have been condensed for space. Branches are colored by support values from phylogenetic analyses, with blue having the highest support. Bootstrap values >50 and posterior probabilities >0.50 are reported near the nodes. Numbers in brackets after taxon names correspond to the number of sequences within the clade. The first number is number of GenBank sequences and the second number, if given, represents number of sequences from this study. Emboldened taxa with three asterisks represent novel species from molecular analyses. (a) Maximum‐likelihood tree. (b) Bayesian inference tree

For both Diplostomidae‐I and Diplostomidae‐II groups, BI and ML phylogenies agreed on minor topologies, with greater support for external nodes and less support and agreement between the two methods for internal nodes (Figure 7). For Diplostomidae‐I, all distance methods in ABGD agreed on 41 total groups (*p*_max_ = 0.059), further supported by the 5% cutoff. A result worth noting from this phylogeny is that a sequence we previously identified as *T. scheuringi* (KT831356) has now split from this group into a separate, new lineage we are now calling *Tylodelphys *sp. A. Several sequences from this study did not group specifically with any available GenBank sequences and have formed distinct lineages among the *Diplostomum* species. These have been identified as *Diplostomum *spp. A–C (Figure 7 and Appendix: Table A10). Other lineage splits seen within *Diplostomum baeri *and *Tylodelphys *sp. 2 have previously been described (Soldánová et al., 2014) and are further supported with our phylogeny.

**Figure 8:**
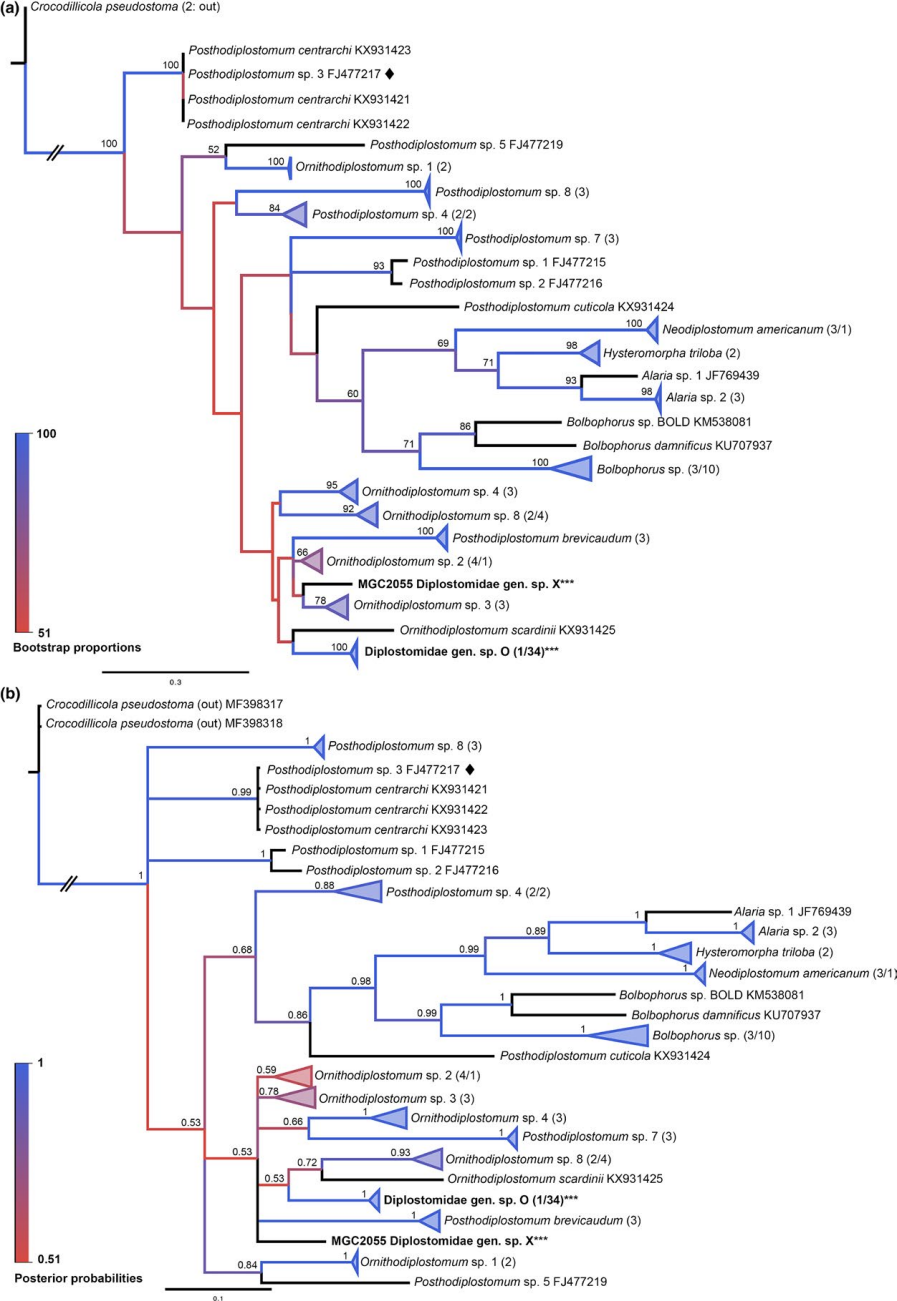
Molecular phylogenies of the Diplostomidae‐II Group based on *cox1*. Clades representing a single species have been condensed for space. Branches are colored by support values from phylogenetic analyses, with blue having the highest support. Bootstrap values >50 and posterior probabilities >0.50 are reported near the nodes. Numbers in parentheses after taxon names correspond to the number of sequences within the clade. The first number is number of GenBank sequences and the second number, if given, represents number of sequences from this study. Emboldened taxa with three asterisks represent novel species from molecular analyses. Black diamonds represent sequences identified uniquely in GenBank that have high similarity and likelihood of being the same as a different species. (a) Maximum‐likelihood tree. (b) Bayesian inference tree

Twenty‐three groups were identified for Diplostomidae‐II, supported by all distance methods of ABGD (*p*_max_ = 0.059) and the 5% cutoff. Two lineages made up of sequences from this study did not group within a specific clade of previously identified sequences and have thus been identified generally as Diplostomidae gen. sp. O and sp. X. One such sequence was previously identified as being most like *Ornithodiplostomum *sp. 4 (KT831363); however, in this phylogeny, it grouped far from the other *Ornithodiplostomum *sequences. Of note is that a sequence from GenBank previously identified as *Posthodiplostomum *sp. 3 (FJ477217) grouped with high statistical support with sequences of *Posthodiplostomum centrarchi *(KX931421–KX931423), supporting a very recent report of this same identification (Stoyanov et al., 2018; Figure 8 and Appendix: Table A11).

### 3.7 Family Strigeidae

**Figure 9:**
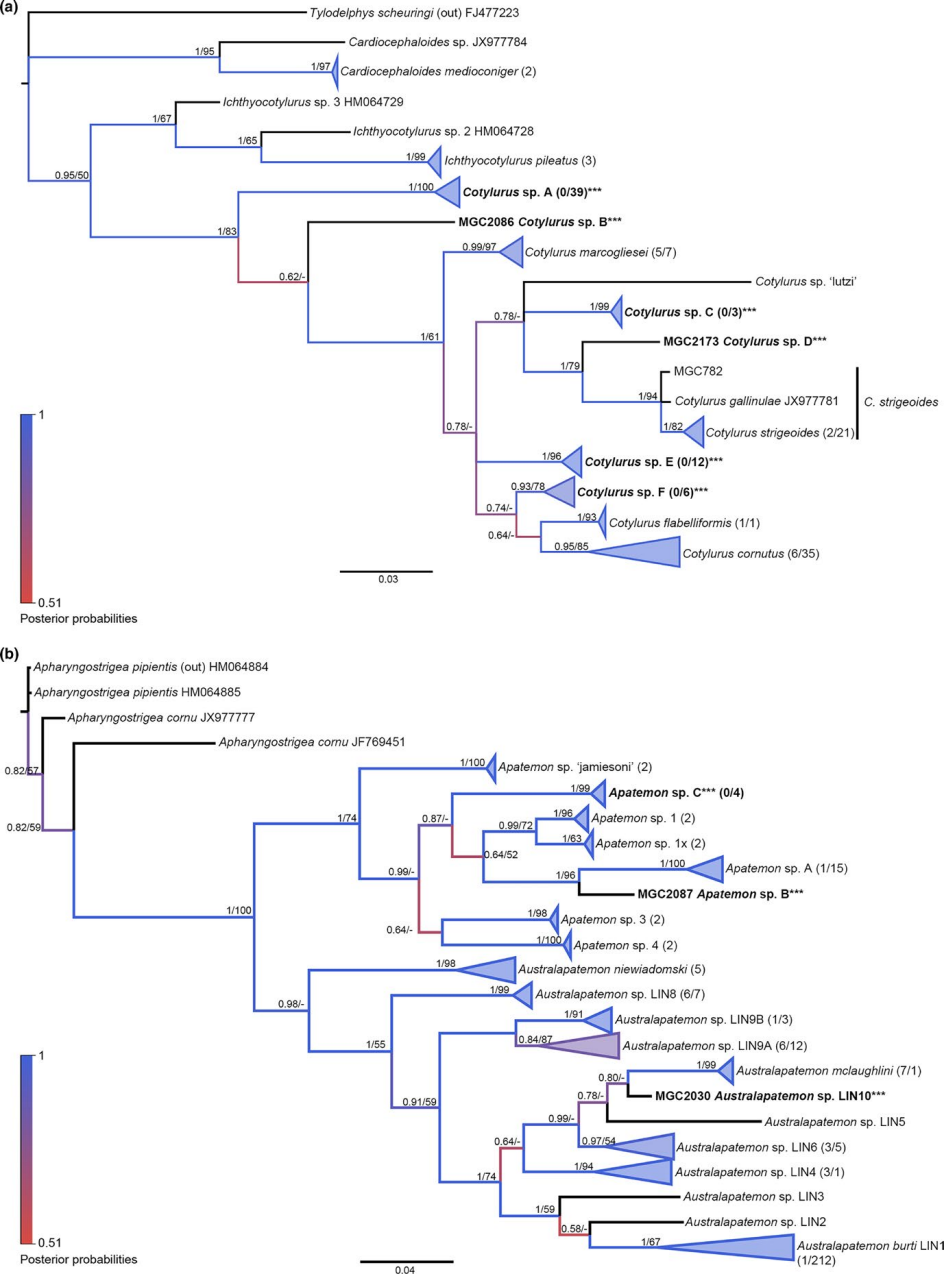
Molecular phylogenies of the Strigeidae based on *cox1*. Bayesian inference phylogenies are given. Clades representing a single species have been condensed for space. Branches are colored by support values from phylogenetic analyses, with blue having the highest support. Posterior probabilities >0.50 and bootstrap values >50 are reported near the nodes, respectively. Accession numbers are given after species names. Numbers in parentheses after taxon names correspond to the number of sequences within the clade. The first number is number of GenBank sequences and the second number, if given, represents number of sequences from this study. Emboldened taxa with three asterisks represent novel species from molecular analyses. (a) Strigeidae‐I. (b) Strigeidae‐II

Few species with sequences across the *cox1* barcoding region were available from GenBank for comparison within the Strigeidae‐I group. At the start of our analyses, only two species had matched with some of our sequences, *Cotylurus cornutus *and *Cotylurus gallinulae*. More recently, more *Cotylurus* species have been added to GenBank (Locke et al., 2018), and these additions helped define three previously unidentifiable lineages from phylogenies. Both ML and BI trees agreed with strong statistical support for the division of all aligned sequences into 16 groups, which was further supported by ABGD (*p*_max (all)_ = 0.0077–0.0129). Sequences from the present study were all more closely related to *Cotylurus *as opposed to *Ichthyocotylurus*, based on *p* distances. Five could be identified to previously named species (*C. cornutus, C. gallinulae, Cotylurus flabelliformis, Cotylurus marcogliesei, *and *Cotylurus strigeoides*), and six other lineages did not match to any GenBank sequences and have been identified as *Cotylurus *sp. A–F. Clade division is further supported by intermediate host use. While intraspecific divergence was within the cutoff for all species, there was lower than expected interspecific divergence between *C. cornutus *and *C. flabelliformis* (4.2%; Figure 9a and Appendix: Table A12).

The Strigeidae‐II group utilized the previously published phylogenies of Blasco‐Costa et al. (1977) and Gordy et al. (2013b) as a starting point, with new sequence additions. Unfortunately, there were still no additional species in GenBank to add that would help inform this phylogeny further. However, the addition of new sequences from the present study has revealed even greater diversity than found previously and has supported the previously derived lineages. While both ML and BI trees agreed on topology and provided medium to strong node support, ABGD methods did not agree with the number of groups informed by previous phylogenies or across methods (JC: 26 groups [*p*_max_ = 0.0077], 21 groups [*p*_max_ = 0.0129–0.0215], 14 groups [*p*_max_ = 0.0359]; K2: 26 groups [*p*_max_ = 0.0129], 21 groups [*p*_max_ = 0.0215–0.0359], 13 groups [*p*_max_ = 0.059]; and Simple: 29 groups [*p*_max_ = 0.007], 28 groups [*p*_max_ = 0.0129], 18 groups [*p*_max_ = 0.0215], 13 groups [*p*_max_ = 0.0359]). Examining divergence based on *p* distances better supported the phylogenetic results, with 23 groups (including out‐group sequences from *Apharyngostrigea *spp.) having been within the 5% intraspecific cutoff and having >5% interspecific divergence, all except for *Australapatemon burti LIN1*, which had an intraspecific divergence range of 0.0%–6.4% and an average of 1.1%. There were only a few sequences that reached the highest part of that range, one new sequence, MGC1629 that came from *S. elodes, *and five previously published sequences: four from Gordy et al. (2013b) (KY587401, HM385485, KY587400, and HM385486), all cercariae derived from *Planorbis *sp. snails in California, and JX977727, an adult from Mexico. Though they differed from some other LIN1 sequences >5%, they were more similar to other LIN1 sequences with divergence <5%, which made it difficult to clearly delineate whether there was one monophyletic clade or more. Currently, there is not enough evidence to clearly support more than one clade within Lineage 1.

Therefore, with the best supported information, there appeared to be 23 groups within the Strigeidae‐II, which revealed three new species of *Apatemon*: species A, which included our previously published *Apatemon *sp. (KT831859), and species B and C. Though these three species all utilized *S. elodes, *they were molecularly divergent.

Within the *Australapatemon *clade, a new lineage appeared from isolate MGC2030 that utilized *P. gyrina* identified herein as Lineage 10. Lineage 9, with the addition of more sequences, as predicted in Gordy et al. (2013b), has revealed the greater likelihood and separation of this lineage into two, which we have called Lineage 9A and Lineage 9B, both of which were hosted by *S. elodes *snails (Figure 9b and Appendix: Table A13).

### 3.8 Species richness and rarefaction

Based on our estimates of species, as described above and evidenced from molecular phylogenies, we have recovered 79 trematode species from five snail host species across six lakes in central Alberta. Richness recoveries were greatest at Isle Lake (38 trematode species/four snail species), followed by Wabamun Lake (27/5), Gull Lake (24/3), Lac La Nonne Site #1 (18/3), Lac La Nonne Site #2 (16/3), Buffalo Lake—Pelican Point (16/3), Buffalo Lake—Rochon Sands (13/2), Buffalo Lake—The Narrows (13/4), and finally, Pigeon Lake Provincial Park (3/1) (Tables 1, 2, 3, 4).

**Table 2:** Trematode species counts by collection site

	Buffalo Lake—Pelican Point	Buffalo Lake—Rochon Sands	Buffalo Lake—The Narrows	Gull Lake—Aspen Beach	Isle Lake	Lac La Nonne	Lac La Nonne site #2	Pigeon Lake—Provincial Park	Wabamun Lake—Provincial Park Beach	Grand total
*Apatemon* sp. A	–	–	–	–	16	–	–	–	–	16
*Apatemon* sp. B	–	–	–	–	1	–	–	–	–	1
*Apatemon* sp. C	–	–	–	–	4	–	–	–	–	4
*Australapatemon burti* LIN1	–	–	2	24	165	2	5	–	6	204
*Australapatemon mclaughlini*	–	2	–	–	–	–	–	–	–	2
*Australapatemon *sp. LIN10	–	–	–	1	–	–	–	–	–	1
*Australapatemon *sp. LIN3	–	–	–	1	–	–	–	–	–	1
*Australapatemon *sp. LIN4	–	–	–	1	–	–	–	–	–	2
*Australapatemon *sp. LIN5	–	1	–	–	–	–	–	–	–	1
*Australapatemon *sp. LIN6	–	–	1	–	4	–	1	1	–	7
*Australapatemon *sp. LIN8	1	4	–	1	3	–	–	–	–	9
*Australapatemon *sp. LIN9A	–	2	–	7	6	2	–	–	–	17
*Australapatemon *sp. LIN9B	3	1	–	–	–	–	–	–	–	4
Avian schistosomatid sp. A	–	3	2	–	2	–	–	–	–	7
Avian schistosomatid sp. B	–	–	–	–	–	–	1	–	–	1
Avian schistosomatid sp. C	–	–	–	–	–	–	–	–	1	1
*Bolbophorus* sp.	–	–	6	–	2	–	–	–	2	10
*Cotylurus cornutus*	–	–	–	2	32	–	1	–	–	35
*Cotylurus flabelliformis*	–	–	–	–	1	–	–	–	–	1
*Cotylurus marcogliesei*	1	–	–	3	2	1	–	–	–	7
*Cotylurus *sp. A	–	–	–	–	34	1	1	–	3	39
*Cotylurus* sp. B	–	–	–	–	1	–	–	–	–	1
*Cotylurus *sp. C	–	–	3	–	–	–	–	–	–	3
*Cotylurus* sp. D	–	1	–	–	–	–	–	–	–	1
*Cotylurus* sp. E	2	1	–	2	5	2	–	–	–	12
*Cotylurus* sp. F	–	–	4	–	–	–	–	–	2	6
*Cotylurus strigeoides*	–	1	1	–	11	1	6	–	2	22
Diplostomidae gen. sp. O	6	19	–	6	2	–	–	–	1	34
Diplostomidae gen. sp. X	–	–	–	–	1	–	–	–	–	1
*Diplostomum baeri *LIN2	–	–	–	–	3	–	–	–	2	5
*Diplostomum indistinctum*	–	–	–	1	–	–	–	–	–	1
*Diplostomum* sp. 1	–	–	–	–	1	–	–	–	5	6
*Diplostomum* sp. 3	–	–	–	–	–	–	–	–	3	3
*Diplostomum* sp. 4	9	–	–	7	24	–	1	–	30	71
*Diplostomum* sp. A	1	–	–	–	–	–	–	–	–	1
*Diplostomum *sp. B	–	–	–	–	1	–	–	–	–	1
*Diplostomum *sp. C	–	–	–	1	10	–	–	–	1	12
*Drepanocephalus spathans*	–	–	3	–	1	–	–	–	–	4
*Echinoparyphium* sp. A	2	–	–	4	22	2	7	2	7	46
*Echinoparyphium* sp. A2	–	–	–	4	–	2	1	–	–	7
*Echinoparyphium* sp. B	–	–	–	–	–	9	–	–	–	9
*Echinoparyphium* sp. C	–	–	–	8	–	16	1	–	–	25
*Echinoparyphium* sp. D	1	–	–	–	–	–	–	–	–	1
*Echinoparyphium* sp. E	–	–	–	6	–	–	–	–	–	6
*Echinoparyphium* sp. Lineage 1A	–	–	–	–	10	18	46	–	23	97
*Echinoparyphium* sp. Lineage 1B	–	–	–	–	1	–	–	–	–	1
*Echinoparyphium* sp. Lineage 2	12	1	–	29	13	27	4	–	3	89
*Echinoparyphium* sp. Lineage 4	–	–	1	–	–	–	–	–	1	2
*Echinostoma revolutum* B	11	–	–	11	2	7	1	–	1	33
*Echinostoma trivolvis *Lineage A	–	–	–	–	2	2	–	–	1	5
Echinostomatidae gen. sp.	4	–	–	–	–	–	–	–	–	4
Haematoloechidae gen. sp. A	4	–	–	–	–	–	–	–	–	4
*Hypoderaeum* sp. Lineage 1	–	–	–	5	–	–	–	–	1	6
*Hypoderaeum* sp. Lineage 2	–	–	–	–	1	2	–	–	–	3
*Neodiplostomum americanum*	*1*	–	–	–	–	–	–	–	–	1
*Neopetasiger islandicus*	–	–	–	–	–	–	–	–	1	1
*Neopetasiger* sp. 4	–	–	1	–	2	–	–	–	7	10
*Notocotylus* sp. A	1	1	–	1	12	9	6	–	9	39
*Notocotylus* sp. B	–	–	–	–	–	–	–	–	1	1
*Notocotylus* sp. C	––	–	–	–	–	1	–	–	–	1
*Notocotylus* sp. D	4	1	–	21	23	4	1	–	–	54
*Ornithodiplostomum *sp. 2	–	–	––	–	–	–	–	–	1	1
*Ornithodiplostomum* sp. 8	–	–	–	–	3	–	–	1	–	4
*Plagiorchis* sp.^a^	343	78	58	257	173	132	26	–	78	1,145
*Posthodiplostomum* sp. 4	–	–	–	–	2	–	–	–	–	2
Psilostomidae gen. sp. A	–	––	–	–	4	–	–	–	2	6
*Schistosomatium douthitti*	–	–	6	3	–	–	–	–	1	10
*Trichobilharzia physellae*	–	–	–	–	–	1	–	–	–	1
*Trichobilharzia stagnicolae*	–	–	–	–	8	–	–	–	–	8
*Trichobilharzia szidati*	–	–	1	1	–	–	–	–	–	2
*Tylodelphys* sp. A	–	–	–	–	–	–	–	–	5	5
Grand total	406	116	89	407	610	241	110	4	200	2,183

a Includes all lineages.

**Table 3:** Host associations, geographical origins, and life stages of specimen sequences used in phylogenies

Family	Species	Host	Host Type	Location	GenBank Accession Number(s)		Reference
					*cox*1	*nad*1	
Diplostomidae	*Alaria* sp. 1	*Lithobates pipiens*	2	Canada: Quebec, Saint Lawrence River	JF769439		(Locke, McLaughlin, Lapierre, Johnson, & Marcogliese, 2011)
Diplostomidae	*Alaria* sp. 2	*Pseudacris regilla, Anaxyrus boreas*	2	USA: California, Bart's Pond; San Martin, Weed Pond	JF904535, JF904534, JF904536		(Locke et al., 2011)
Diplostomidae	*Austrodiplostomum ostrowskiae*	*Biomphalaria obstructa, Dorosoma cepedianum*	1, 2	USA: Noxubee County, MS;USA: Dallas County, AL	KT728795, KT728798, KT728799		(Rosser et al., 2016)
Diplostomidae	*Austrodiplostomum* sp. 1	*Pomoxis nigromaculatus*	2	USA: Florida, Tampa, Lake Seminole	KR271029		(Locke et al., 2015)
Diplostomidae	*Austrodiplostomum* sp. 2	*Mugil cephalus*	2	USA: Florida, Tampa, Lake Seminole	KR271032		(Locke et al., 2015)
Diplostomidae	*Austrodiplostomum* sp. 2	*Menidia beryllina, Ictalurus punctatus*	2	USA: Mississippi	KU707943, KU707945		(Rosser et al., 2016)
Diplostomidae	*Bolbophorus damnificus*	*Menidia beryllina*	2	USA: Mississippi	KU707937		(Rosser et al., 2016)
Diplostomidae	*Bolbophorus* sp.	*Pimephales promelas*	2	Canada: Alberta, Coaldale, McQuillan Lake	KM538081		(Van Steenkiste, Locke, Castelin, Marcogliese, & Abbott, 2014)
Diplostomidae	*Bolbophorus* sp.	*Menidia beryllina*	2	USA: Mississippi	KU707938, KU707939		(Rosser et al., 2016)
Diplostomidae	*Bolbophorus* sp.	*Helisoma trivolvis*	1	Canada: Alberta, Buffalo Lake	KT831373		(Gordy, Kish, Tarrabain, & Hanington, 2016)
Diplostomidae	*Bolbophorus* sp.	*Helisoma trivolvis*	1	Canada: Alberta, Buffalo Lake, Isle Lake, Wabamun Lake	MH368843, MH368847, MH368850, MH368862, MH368871, MH368892, MH368918, MH368919		Present study
Diplostomidae	*Crocodillicola pseudostoma* (out)	*Rhamdia guatemalensis*	2	Mexico: Veracruz, Catemaco Lake	MF398317, MF398318		(Hernández‐Mena, García‐Varela, & Pérez‐Ponce de León, 2017)
Diplostomidae	*Diplostomidae* gen. sp. O ^***^	*Physa gyrina*	1	Canada: Alberta, Buffalo Lake	KT831363^§^		(Gordy et al., 2016)
Diplostomidae	*Diplostomidae* gen. sp. O^***^	*Physa gyrina*	1	Canada: Alberta, Buffalo Lake, Wabamun Lake, Gull Lake, Isle Lake	MH368825, MH368851, MH368854, MH368855, MH368879, MH368880, MH368881, MH368882, MH368883, MH368884, MH368885, MH368886, MH368887, MH368888, MH368889, MH368890, MH368893, MH368903, MH368904, MH368905, MH368906, MH368915, MH368916, MH368917, MH368934, MH368935, MH368936, MH368937, MH368938, MH368939, MH368940, MH368941, MH368942		Present study
Diplostomidae	*Diplostomidae* gen. sp. X^***^	*Physa gyrina*	1	Canada: Alberta, Isle Lake	MH368907		Present study
Diplostomidae	*Diplostomum ardeae*	*Ardea herodias*	3	Canada: Quebec, Montreal	KR271033		(Locke et al., 2015)
Diplostomidae	*Diplostomum baeri* LIN1	*Perca fluviatilis*	2	Germany: Lake Constance	JQ639181, JQ639182		(Behrmann‐Godel, 2013)
Diplostomidae	*Diplostomum baeri* LIN2	Not given	3	Canada: Quebec, Montreal	GQ292501		(Locke, McLaughlin, Dayanandan, & Marcogliese, 2010)
Diplostomidae	*Diplostomum baeri* LIN2	*Stagnicola elodes*	1	Canada: Alberta, Wabamun Lake, Isle Lake	MH368863, MH368874, MH368875, MH368928		Present study
Diplostomidae	*Diplostomum huronense*	*Notemigonus crysoleuca, Larus delawarensis*	2, 3	Canada: Ontario	FJ477197		(Moszczynska, Locke, McLaughlin, Marcogliese, & Crease, 2009)
Diplostomidae	*Diplostomum huronense*	*Perca flavescens, Notemigonus crysoleucas*	2	Canada: Quebec, St. Lawrence River, Lake Saint Louis, Beauharnois, Dorval Island	HM064671, HM064672		(Locke, McLaughlin, Dayanandan, et al., 2010)
Diplostomidae	*Diplostomum indistinctum*	*Catostomidae*	3	Canada: Quebec	FJ477196		(Moszczynska et al., 2009)
Diplostomidae	*Diplostomum indistinctum*	*Neogobius melanostomus*	2	Canada: Quebec	GQ292482		(Locke, McLaughlin, Dayanandan, et al., 2010)
Diplostomidae	*Diplostomum indistinctum*	*Catostomus commersoni*	2	Canada: Quebec, St. Lawrence River, Lake Saint	HM064673		(Locke, McLaughlin, & Marcogliese, 2010)
Diplostomidae	*Diplostomum indistinctum*	*Stagnicola elodes*	1	Canada: Alberta, Gull Lake	KT831379		(Gordy et al., 2016)
Diplostomidae	*Diplostomum mergi*	*Radix auricularia*	1	Germany: Hengsteysee	KR149526, KR149527, KR149528		(Selbach, Soldánová, Georgieva, Kostadinova, & Sures, 2015)
Diplostomidae	*Diplostomum parviventosum*	*Radix auricularia*	1	Germany: Hengsteysee	KR149510, KR149511, KR149512		(Selbach et al., 2015)
Diplostomidae	*Diplostomum pseudospathaceum*	*Stagnicola palustris*	1	Germany: Hengsteysee	KR149544, KR149545, KR149546		(Selbach et al., 2015)
Diplostomidae	*Diplostomum* sp. 1	*Larus delawarensis*	3	Canada: Quebec, Laurentides	GQ292479, GQ292480, GQ292481		(Locke, McLaughlin, Dayanandan, et al., 2010)
Diplostomidae	*Diplostomum* sp. 1	*Stagnicola elodes*	1	Canada: Alberta, Wabamun Lake, Isle Lake	MH368857, MH368896, MH368932, MH368943, MH368945		Present study
Diplostomidae	*Diplostomum* sp. 2	*Pimephales notatus*	2	Canada: Quebec, St. Lawrence River	GQ292486		(Locke, McLaughlin, Dayanandan, et al., 2010)
Diplostomidae	*Diplostomum* sp. 3	*Micropterus salmoides*	2	Canada: Quebec, St. Lawrence River	GQ292487		(Locke, McLaughlin, Dayanandan, et al., 2010)
Diplostomidae	*Diplostomum* sp. 3	*Lymnaea stagnalis*	1	Canada: Alberta, Wabamun Lake	KT831358		(Gordy et al., 2016)
Diplostomidae	*Diplostomum* sp. 3	*Lymnaea stagnalis*	1	Canada: Alberta, Wabamun Lake	MH368837, MH368858		Present study
Diplostomidae	*Diplostomum* sp. 4	*Larus delawarensis*	3	Canada: Quebec, Laurentides	GQ292494, GQ292495		(Locke, McLaughlin, Dayanandan, et al., 2010)
Diplostomidae	*Diplostomum* sp. 4	*Stagnicola elodes*	1	Canada: Alberta, Isle Lake	KT831354		(Gordy et al., 2016)
Diplostomidae	Diplostomum sp. 4	Stagnicola elodes	1	Canada: Alberta, Wabamun Lake, Isle Lake, Gull Lake, Buffalo Lake, Lac La Nonne	MH368808, MH368809, MH368813, MH368814, MH368815, MH368816, MH368818, MH368819, MH368820, MH368821, MH368822, MH368823, MH368824, MH368826, MH368827, MH368828, MH368829, MH368830, MH368831, MH368832, MH368833, MH368834, MH368835, MH368836, MH368838, MH368839, MH368840, MH368841, MH368844, MH368845, MH368846, MH368848, MH368849, MH368853, MH368856, MH368859, MH368860, MH368861, MH368864, MH368865, MH368866, MH368867, MH368868, MH368869, MH368870, MH368872, MH368873, MH368876, MH368877, MH368891, MH368898, MH368899, MH368900, MH368901, MH368911, MH368913, MH368914, MH368924, MH368925, MH368926, MH368927, MH368929, MH368930, MH368931, MH368944, MH368946, MH368947, MH368948, MH368949, MH368950		Present study
Diplostomidae	*Diplostomum* sp. 6	*Pimephales notatus*	2	Canada: Quebec, St. Lawrence River	GQ292499		(Locke, McLaughlin, Dayanandan, et al., 2010)
Diplostomidae	*Diplostomum* sp. 7	*Pimephales notatus*	2	Canada: Quebec, St. Lawrence River	GQ292500		(Locke, McLaughlin, Dayanandan, et al., 2010)
Diplostomidae	*Diplostomum* sp. 8	*Rana pipiens*	2	Canada: Quebec, Monteregie	GQ292497		(Locke, McLaughlin, Dayanandan, et al., 2010)
Diplostomidae	*Diplostomum* sp. 9	*Percina caprodes*	2	Canada: Quebec, St. Lawrence River	GQ292496		(Locke, McLaughlin, Dayanandan, et al., 2010)
Diplostomidae	*Diplostomum* sp. A^***^	*Stagnicola elodes*	1	Canada: Alberta, Buffalo Lake	MH368817		Present study
Diplostomidae	*Diplostomum* sp. B^***^	*Stagnicola elodes*	1	Canada: Alberta, Isle Lake	MH368933		Present study
Diplostomidae	*Diplostomum* sp. C^***^	*Stagnicola elodes*	1	Canada: Alberta, Gull Lake, Wabamun Lake, Isle Lake	KT831360^§^, KT831378^§^, KT831382^§^		(Gordy et al., 2016)
Diplostomidae	*Diplostomum* sp. C^***^	*Stagnicola elodes, Helisoma trivolvis* (MGC208)	1	Canada: Alberta, Gull Lake, Wabamun Lake, Isle Lake	MH368810, MH368811, MH368812, MH368852, MH368895, MH368902, MH368921, MH368922, MH368923		Present study
Diplostomidae	*Diplostomum* sp. clade Q	*Radix auricularia*	1	Germany: Hengsteysee	KR149554		(Selbach et al., 2015)
Diplostomidae	*Diplostomum* sp. LIN6	*Gasterosteus aculeatus*	2	Norway: Troms, Takvatnet	KM212051, KM212052, KM212053		(Kuhn et al., 2015)
Diplostomidae	*Diplostomum spathaceum*	*Acanthobrama marmid, Perca fluviatilis, Barbus luteus*	2	Iraq: Saladin, Tikreet, Tigris River; Italy: Lecco, Lake Como, Oliveto Lario	KR271467, KR271468, KR271469		(Locke, McLaughlin, Dayanandan, et al., 2010)
Diplostomidae	*Diplostomum spathaceum*	*unknown*		unknown, likely China	KT736038		Dang, R., et al., 2015, Unpublished
Diplostomidae	*Hysteromorpha triloba*	*Catostomus, Notemigonus crysoleucas*	2	Canada: Nova Scotia, Sackville, Feely Lake; Canada: Quebec, Outaouais, Ottawa River, Wendover	JF769475, JF769476		(Locke et al., 2011)
Diplostomidae	*Neodiplostomum americanum*	*Lithobates aurora*	2	USA: California, HMB 05	JF904537, JF904538, JF769455		(Locke et al., 2011)
Diplostomidae	*Neodiplostomum americanum*	*Stagnicola elodes*	1	Canada: Alberta, Buffalo Lake	KT831357^§^		(Gordy et al., 2016)
Diplostomidae	*Ornithodiplostomum scardinii*	*Scardinius erythrophthalmus*	2	Czech Republic: Lake Macha	KX931425		(Stoyanov et al., 2017)
Diplostomidae	*Ornithodiplostomum scardinii (out)*	*Scardinius erythrophthalmus*	2	Czech Republic: Lake Macha	KX931425		(Stoyanov et al., 2017)
Diplostomidae	*Ornithodiplostomum* sp. 1	*Etheostoma nigrum*	2	Canada: Ontario, St. Lawrence River	FJ477208		(Moszczynska et al., 2009)
Diplostomidae	*Ornithodiplostomum* sp. 1	*Etheostoma nigrum*	2	Canada: Quebec, St. Lawrence River, Lake Saint Francois, Pointe Dupuis (LSF‐2)	HM064742		(Locke, McLaughlin, & Marcogliese, 2010)
Diplostomidae	*Ornithodiplostomum* sp. 2	*Physa gyrina*	1	Canada: Alberta, Wabamun Lake	KT831368		(Gordy et al., 2016)
Diplostomidae	*Ornithodiplostomum* sp. 2	*Notemigonus crysoleucas*	2	Canada: Quebec, St. Lawrence River, Lake Saint Louis, Beauharnois	HM064766, HM064768		(Locke, McLaughlin, & Marcogliese, 2010)
Diplostomidae	*Ornithodiplostomum* sp. 2	*Notemigonus crysoleucas*	2	Canada: Quebec, St. Lawrence River, Lake Saint Louis	FJ477210		(Moszczynska et al., 2009)
Diplostomidae	*Ornithodiplostomum* sp. 2	*Physa gyrina*	1	Canada: Alberta, Wabamun Lake	KT831368		(Gordy et al., 2016)
Diplostomidae	*Ornithodiplostomum* sp. 3	*Pimephales notatus*	2	Canada: Quebec, St. Lawrence River, Lake Saint Francois, Pointe Dupuis (LSF‐2), Beauharnois	HM064782, HM064780		(Locke, McLaughlin, & Marcogliese, 2010)
Diplostomidae	*Ornithodiplostomum* sp. 3	*Pimephales notatus*	2	Canada: Quebec, St. Lawrence River, Lake Saint Francois, Pointe Dupuis (LSF‐2), Beauharnois	FJ477211		(Moszczynska et al., 2009)
Diplostomidae	*Ornithodiplostomum* sp. 4	*Pimephales notatus*	2	Canada: Quebec, St. Lawrence River, Lake Saint Francois, Pointe Dupuis (LSF‐2), Beauharnois	HM064786, HM064788		(Locke, McLaughlin, & Marcogliese, 2010)
Diplostomidae	*Ornithodiplostomum* sp. 4	*Pimephales notatus*	2	Canada: Quebec, St. Lawrence River, Lake Saint Francois, Pointe Dupuis (LSF‐2), Beauharnois	FJ477212		(Moszczynska et al., 2009)
Diplostomidae	*Ornithodiplostomum* sp. 8	*Pimephales notatus*	2	Canada: Quebec, St. Lawrence River, Lake Saint Pierre, Ile aux Ours	HM064789		(Locke, McLaughlin, & Marcogliese, 2010)
Diplostomidae	*Ornithodiplostomum* sp. 8	*Physa gyrina*	1	Canada: Alberta, Pigeon Lake	KT831383		(Gordy et al., 2016)
Diplostomidae	*Ornithodiplostomum* sp. 8	*Physa gyrina*	1	Canada: Alberta, Isle Lake	MH368908, MH368910, MH368920		Present study
Diplostomidae	*Posthodiplostomum brevicaudatum*	*Perca fluviatilis, Gasterosteus aculeatus*	2	Czech Republic: Lake Macha; Bulgaria: Lake Atanasovsko	KX931418, KX931419, KX931420		(Stoyanov et al., 2017)
Diplostomidae	*Posthodiplostomum centrarchi*	*Lepomis gibbosus, Ardea cinerea*	2, 3	Bulgaria: Lake Atanasovsko; Spain: Lagoon Bassa de les Olles, Ebro Delta; Slovakia: River Danube near Sturovo	KX931421, KX931422, KX931423		(Stoyanov et al., 2017)
Diplostomidae	*Posthodiplostomum cuticola*	*Planorbis planorbis*	1	Lithuania: Curonian Bay near Juodkrante	KX931424		(Stoyanov et al., 2017)
Diplostomidae	*Posthodiplostomum* sp. 1	*Ambloplites rupestris*	2	Canada: Ontario, St. Lawrence River	FJ477215		(Moszczynska et al., 2009)
Diplostomidae	*Posthodiplostomum* sp. 2	*Lepomis gibbosus*	2	Canada: Quebec, St. Lawrence River, Lake Saint Pierre, Ile aux Ours	FJ477216		(Moszczynska et al., 2009)
Diplostomidae	*Posthodiplostomum* sp. 3^*^	*Lepomis gibbosus*	2	Canada: Quebec, St. Lawrence River, Beauharnois	FJ477217		(Moszczynska et al., 2009)
Diplostomidae	*Posthodiplostomum* sp. 4	*Lepomis gibbosus*	2	Canada: Quebec, St. Lawrence River, Lake Saint Pierre, Ile aux Ours	FJ477218		(Moszczynska et al., 2009)
Diplostomidae	*Posthodiplostomum* sp. 4	*Ardea herodias*	3	Canada: Quebec, Lac Saint‐Pierre, Grand Ile	HM064844		(Locke, McLaughlin, & Marcogliese, 2010)
Diplostomidae	*Posthodiplostomum* sp. 4	*Physa gyrina*	1	Canada: Alberta, Isle Lake	MH368909, MH368912		Present study
Diplostomidae	*Posthodiplostomum* sp. 5	*Lepomis gibbosus*	2	Canada: Quebec, St. Lawrence River, Lake Saint Pierre, Iles aux Sables	FJ477219		(Moszczynska et al., 2009)
Diplostomidae	*Posthodiplostomum* sp. 7	*Perca flavescens*	2	Canada: Quebec, St. Lawrence River, Lake Saint Pierre, Iles aux Sables	FJ477221		(Moszczynska et al., 2009)
Diplostomidae	*Posthodiplostomum* sp. 7	*Perca flavescens*	2	Canada: Quebec, St. Lawrence River, Beauharnois	HM064865, HM064871		(Locke, McLaughlin, & Marcogliese, 2010)
Diplostomidae	*Posthodiplostomum* sp. 8	*Micropterus dolomieu*	2	Canada: Quebec, St. Lawrence River, Lake Saint Pierre, Iles aux Sables	HM064873, HM064874, HM064875		(Locke, McLaughlin, & Marcogliese, 2010)
Diplostomidae	*Tylodelphys aztecae*	*Skiffia lermae, Gila conspersa*	2	Mexico	KT175367, KT175368, KT175369		(García‐Varela, Sereno‐Uribe, Pinacho‐Pinacho, Domínguez‐Domínguez, & Pérez‐Ponce de León, 2016)
Diplostomidae	*Tylodelphys clavata*	*Perca fluviatilis*	2	Germany: Lake Constance	JQ639201, JQ639202, JQ639203, JQ639204		(Behrmann‐Godel, 2013)
Diplostomidae	*Tylodelphys clavata*	*Radix auricularia*	1	Germany: Hengsteysee	JX986908		(Georgieva, Soldánová, et al., 2013)
Diplostomidae	*Tylodelphys clavata*	*Perca fluviatilis*	2	Romania: Danube Delta;Italy: Lombardy, Brescia, Oglio River;Italy: Lecco, Lake Como, Oliveto Lario	KR271478, KR271479, KR271480		(Locke et al., 2015)
Diplostomidae	*Tylodelphys excavata*	*Planorbarius corneus*	1	Czech Republic: Pond Bohdanec	KC685344		(Chibwana et al., 2013)
Diplostomidae	*Tylodelphys immer*	*Salvelinus fontinalis, Gavia immer*	2, 3	Canada: Quebec, Bas‐Saint‐Laurent, Central, riviere Bic; Montreal	KR271491, KR271492, KR271493		(Locke et al., 2015)
Diplostomidae	*Tylodelphys jenynsiae*	*Cnesterodon decemmaculatus*	2	Argentina: Buenos Aires, La Plata, Urban canal	KR271494, KR271495, KR271496		(Locke et al., 2015)
Diplostomidae	*Tylodelphys mashonensis*	*Clarias gariepinus*	2	Tanzania: River Msimbazi, River Ruvu	KC685340, KC685341, KC685342, KC685343		(Chibwana et al., 2013)
Diplostomidae	*Tylodelphys scheuringi*	*Ambloplites rupestris*	2	Canada: Quebec, St. Lawrence River, Lake Saint Pierre, Iles aux Sables	FJ477223		(Moszczynska et al., 2009)
Diplostomidae	*Tylodelphys scheuringi*	*Perca flavescens, Ambloplites rupestris*	2	Canada: Ontario, St. Lawrence River, Lake Saint Francois, LSF‐1; Lake Saint Louis, Dorval Island	HM064914, HM064915		(Locke, McLaughlin, & Marcogliese, 2010)
Diplostomidae	*Tylodelphys scheuringi*	*Ambloplites rupestris, Perca flavescens*	2	Canada: Ontario, St. Lawrence River, Pointe Dupuis (LSF‐2)	KR271508, KR271509		(Chibwana et al., 2013)
Diplostomidae	*Tylodelphys* sp.	*Mystus tengara*	2	India	KU725888, KU725889		Chaudhary, A., et al., 2016, Unpublished
Diplostomidae	*Tylodelphys* sp.	*Gobiomorphus cotidianus*	2	New Zealand	KU588147, KU588148, KU588149		(Blasco‐Costa, Poulin, & Presswell, 2016)
Diplostomidae	*Tylodelphys* sp. 2 LIN1	*Clarias gariepinus*	2	Tanzania: Lake Victoria	KC685358		(Chibwana et al., 2013)
Diplostomidae	*Tylodelphys* sp. 2 LIN2	*Micropterus salmoides, Oreochromis leucostictus*	2	Kenya: Rift Valley, Nakuru District, Lake Naivasha	KF809488, KF809494		(Otachi, Locke, Jirsa, Fellner‐Frank, & Marcogliese, 2015)
Diplostomidae	*Tylodelphys* sp. 3	*Lepomis microlophus*	2	USA: Mississippi, Ascension Parish	KR271513, KR271514, KR271515		(Locke et al., 2015)
Diplostomidae	*Tylodelphys* sp. 4	*Gobiomorus maculatus*	2	Mexico: Oaxaca, Costa Chica, Playa Ventanilla, Laguna Ventanilla	KR271517, KR271518, KR271519		(Locke et al., 2015)
Diplostomidae	*Tylodelphys* sp. 5	*Dormitator latifrons, Gobiomorus maculatus*	2	Mexico: Oaxaca, Costa Chica, Playa Ventanilla, Laguna Ventanilla	KR271520, KR271521		(Locke et al., 2015)
Diplostomidae	*Tylodelphys* sp. 6	*Poecilia latipinna*	2	USA: Mississippi, Ascension Parish	KR271522, KR271523		(Locke et al., 2015)
Diplostomidae	*Tylodelphys* sp. A^***^	*Helisoma trivolvis*	1	Canada: Alberta, Wabamun Lake	KT831356^§^		(Gordy et al., 2016)
Diplostomidae	*Tylodelphys* sp. A^***^	*Helisoma trivolvis*	1	Canada: Alberta, Wabamun Lake	MH368842, MH368878, MH368894, MH368897		Present study
Echinostomatidae	*Drepanocephalus auritus*	*Planorbella trivolvis, Biomphalaria straminea*	1	USA; Brazil		KP053262, KP053263	(Pinto, Griffin, Quiniou, Ware, & Melo, 2016)
Echinostomatidae	*Drepanocephalus auritus*	*Helisoma trivolvis*	1	Canada: Alberta, Isle Lake, Buffalo Lake		MH368951, MH368952	Present Study
Echinostomatidae	*Drepanocephalus auritus*	*Phalacrocorax auritus*	3	Canada: Ontario, Lake Erie	KM538090		(Van Steenkiste et al., 2014)
Echinostomatidae	*Drepanocephalus auritus*	*Phalacrocorax auritus*	3	USA: lake Near Lakota, Nelson County, North Dakota; Lower Red Lake, Beltrami County, Minnesota; George County, Mississippi	KP683125, KP683126, KP683127, KP638128, KP683129, K638130, KP638131, KP638132		(Kudlai, Kostadinova, Pulis, & Tkach, 2015)
Echinostomatidae	*Drepanocephalus auritus*	*Planorbella trivolvis*	1	USA	KR259644		(Pinto et al., 2016)
Echinostomatidae	*Drepanocephalus auritus*	*Helisoma trivolvis*	1	Canada: Alberta, Buffalo Lake	KT831381		(Gordy et al., 2016)
Echinostomatidae	*Drepanocephalus auritus*	*Helisoma trivolvis*	1	Canada: Alberta, Buffalo Lake, Isle Lake	MH369294		Present study
Echinostomatidae	*Drepanocephalus mexicanus*	*Phalacrocorax brasilianus*	3	Mexico: Tobasco, Teapa	KY636228, KY636229		(Hernández‐Cruz, Hernández‐Orts, Sereno‐Uribe, Pérez‐Ponce de León, & García‐Varela, 2018)
Echinostomatidae	*Drepanocephalus* sp.	*Biomphalaria straminea*	1	Brazil		KP05264	(Pinto et al., 2016)
Echinostomatidae	*Drepanocephalus spathans*	*Phalacrocorax brasilianus*	3	Mexico: Durango, Rio Guatimape; Oaxaca, Presa Rio Verde	KY636233, KY636234		(Hernández‐Cruz et al., 2018)
Echinostomatidae	*Echinoparyphium aconiatum*	*Lymnaea stagnalis*	1, 2	Finland: Lake Pyykosjarvi		AY168946, AY168947	(Kostadinova & Herniou, 2003)
Echinostomatidae	*Echinoparyphium ellisi*	*Anas platyrhynchos*	3	New Zealand: Clutha River System, Central Otago District, South Island		KY436405, KY436406	(Stoyanov et al., 2017)
Echinostomatidae	*Echinoparyphium poulini*	*Cygnus atratus*	3	New Zealand: Pauerau, Central Otago District, South Island		KY436403, KY436404	(Stoyanov et al., 2017)
Echinostomatidae	*Echinoparyphium recurvatum*	*Lymnaea peregra*	1	UK: Wales, Lake Ceunant		AY168943, AY168944	(Kostadinova & Herniou, 2003)
Echinostomatidae	*Echinoparyphium recurvatum*	*Pisidium casertanum, Sphaerium sp*.	2	Norway: Lake Takvatn		KY513267, KY513269	(Soldánová et al., 2017)
Echinostomatidae	*Echinoparyphium* sp. 1A^***^	*Physa gyrina, Stagnicola elodes (MGC1954, MGC2104), Helisoma trivolvis (MGC2090)*	1	Canada: Alberta, Lac La Nonne, Wabamun Lake, Isle Lake		MH368998, MH368999, MH369001, MH369002, MH369003, MH369004, MH369005, MH369006, MH369007, MH369008, MH369009, MH369010, MH369012, MH369013, MH369014, MH369015, MH369016, MH369017, MH369018, MH369019, MH369022, MH369023, MH369024, MH369025, MH369026, MH369028, MH369031, MH369032, MH369033, MH369034, MH369038, MH369042, MH369044, MH369045, MH369046, MH369047, MH369048, MH369049, MH369052, MH369053, MH369054, MH369055, MH369056, MH369059, MH369060, MH369062, MH369063, MH369065, MH369066, MH369068, MH369070, MH369075, MH369076, MH369087, MH369089, MH369090, MH369091, MH369093, MH369094, MH369095, MH369096, MH369097, MH369098, MH369099, MH369100, MH369101, MH369102, MH369121, MH369122, MH369123, MH369125, MH369131, MH369132, MH369133, MH369136, MH369147, MH369155, MH369156, MH369162, MH369163, MH369164, MH369165, MH369166, MH369167, MH369168, MH369178, MH369188, MH369191	Present study
Echinostomatidae	*Echinoparyphium* sp. 1A^***^	*Physa gyrina*	1	Canada: Alberta, Lac La Nonne	KT831361^§^		(Gordy et al., 2016)
Echinostomatidae	*Echinoparyphium* sp. 1A^***^	*Physa gyrina, Stagnicola elodes (MGC1954, MGC2104), Helisoma trivolvis (MGC2090)*	1	Canada: Alberta, Lac La Nonne, Wabamun Lake, Isle Lake	MH369243, MH369245, MH369246, MH369249, MH369250, MH369253, MH369255, MH369272, MH369273, MH369274, MH369277, MH369299, MH369300, MH369301, MH369302, MH369303, MH369304, MH369305		Present study
Echinostomatidae	*Echinoparyphium* sp. 1B^***^	*Physa gyrina*	1	Canada: Alberta, Isle Lake		MH369181	Present study
Echinostomatidae	*Echinoparyphium* sp. A^***^	*Physa gyrina, Stagnicola elodes (MGC1932)*	1	Canada: Alberta, Buffalo Lake, Wabamun Lake, Isle Lake, Lac La Nonne, Gull Lake, Pigeon Lake		MH369011, MH369035, MH369043, MH369051, MH369058, MH369061, MH369064, MH369069, MH369081, MH369082, MH369083, MH369084, MH369085, MH369113, MH369120, MH369128, MH369161, MH369169, MH369170, MH369171, MH369172, MH369173, MH369174, MH369175, MH369176, MH369177, MH369179, MH369180, MH369182, MH369183, MH369184, MH369185, MH369187, MH369190	Present study
Echinostomatidae	*Echinoparyphium* sp. A^***^	*Physa gyrina, Stagnicola elodes (MGC1932)*	1	Canada: Alberta, Buffalo Lake, Wabamun Lake, Isle Lake, Lac La Nonne, Gull Lake, Pigeon Lake	MH369223, MH369247, MH369254, MH369257, MH369266, MH369289, MH369290, MH369291, MH369298, MH369306, MH369307, MH369308, MH369309, MH369310		Present study
Echinostomatidae	*Echinoparyphium* sp. A2^***^	*Stagnicola elodes*	1	Canada: Alberta, Gull Lake, Lac La Nonne		MH369181	Present study
Echinostomatidae	*Echinoparyphium* sp. A2^***^	*Stagnicola elodes*	1	Canada: Alberta, Lac La Nonne	KT831367^§^		(Gordy et al., 2016)
Echinostomatidae	*Echinoparyphium* sp. A2^***^	*Stagnicola elodes*	1	Canada: Alberta, Gull Lake, Lac La Nonne	MH369232, MH369251, MH369258, MH369260, MH369265, MH369288		Present study
Echinostomatidae	*Echinoparyphium* sp. B^***^	*Stagnicola elodes*	1	Canada: Alberta, Lac La Nonne		MH368969, MH368970, MH368971, MH368987, MH368988, MH369041, MH369074, MH369086, MH369092	Present study
Echinostomatidae	*Echinoparyphium* sp. C^***^	*Stagnicola elodes*	1	Canada: Alberta, Gull Lake, Lac La Nonne		MH369088, MH369152	Present study
Echinostomatidae	*Echinoparyphium* sp. C^***^	*Stagnicola elodes*	1	Canada: Alberta, Gull Lake, Lac La Nonne	MH369226, MH369228, MH369233, MH369234, MH369236, MH369237, MH369238, MH369239, MH369240, MH369241, MH369244, MH369252, MH369256, MH369259, MH369261, MH369262, MH369263, MH369264, MH369267, MH369278, MH369280, MH369282, MH369285, MH369296		Present study
Echinostomatidae	*Echinoparyphium* sp. D^***^	*Stagnicola elodes*	1	Canada: Alberta, Buffalo Lake		MH369189	Present study
Echinostomatidae	*Echinoparyphium* sp. E^***^	*Stagnicola elodes, Lymnaea stagnalis (MGC1878)*	1	Canada: Alberta, Gull Lake		MH369109, MH369129, MH369134, MH369135, MH369159	Present study
Echinostomatidae	*Echinoparyphium* sp. E^***^	*Stagnicola elodes, Lymnaea stagnalis (MGC1878)*	1	Canada: Alberta, Gull Lake	MH369275, MH369276		Present study
Echinostomatidae	*Echinoparyphium* sp. Lineage 1	*Ondatra zibethicus*	3	USA: Wisconsin		GQ463103, GQ463104, GQ463105	(Detwiler, Bos, & Minchella, 2010)
Echinostomatidae	*Echinoparyphium* sp. Lineage 2	*Stagnicola elodes, Lymnaea stagnalis (MGC16A/B, MGC369), Helisoma trivolvis (MGC219)*	1	Canada: Alberta, Gull Lake, Isle Lake, Buffalo Lake, Wabamun Lake, Lac La Nonne		MH368953, MH368954, MH368955, MH368956, MH368957, MH368959, MH368960, MH368961, MH368962, MH368963, MH368964, MH368965, MH368966, MH368967, MH368968, MH368972, MH368973, MH368974, MH368975, MH368976, MH368977, MH368978, MH368979, MH368980, MH368981, MH368982, MH368983, MH368984, MH368985, MH368986, MH368989, MH368990, MH368991, MH368992, MH368993, MH368994, MH368995, MH368996, MH368997, MH369000, MH369021, MH369027, MH369029, MH369036, MH369037, MH369039, MH369050, MH369057, MH369067, MH369071, MH369072, MH369073, MH369077, MH369078, MH369079, MH369103, MH369104, MH369105, MH369106, MH369107, MH369111, MH369112, MH369114, MH369115, MH369116, MH369117, MH369118, MH369119, MH369124, MH369126, MH369137, MH369138, MH369139, MH369140, MH369141, MH369142, MH369143, MH369144, MH369146, MH369148, MH369149, MH369150, MH369151, MH369153, MH369154, MH369160, MH369186	Present study
Echinostomatidae	*Echinoparyphium* sp. Lineage 2	*Lymnaea elodes*	1	USA: Indiana, Pond A		GQ463119, GQ463120, GQ463121	(Detwiler et al., 2010)
Echinostomatidae	*Echinoparyphium* sp. Lineage 2	*Stagnicola elodes, Lymnaea stagnalis (MGC16A/B, MGC369), Helisoma trivolvis (MGC219)*	1	Canada: Alberta, Gull Lake, Isle Lake, Buffalo Lake, Wabamun Lake, Lac La Nonne	MH369224, MH369225, MH369283, MH369293		Present study
Echinostomatidae	*Echinoparyphium* sp. Lineage 2	*Stagnicola elodes*	1	Canada: Alberta, Lac La Nonne	KT831350^§^		(Gordy et al., 2016)
Echinostomatidae	*Echinoparyphium* sp. Lineage 3	*Helisoma trivolvis*	1	Canada: Alberta, Wabamun Lake, Buffalo Lake		MH369130, MH369158	Present study
Echinostomatidae	*Echinoparyphium* sp. Lineage 3	*Helisoma trivolvis*	1, 2	USA: Indiana, Pond A		GQ463122, GQ463123	(Detwiler et al., 2010)
Echinostomatidae	*Echinoparyphium* sp. Lineage 3	*Helisoma trivolvis*	1	Canada: Alberta, Wabamun Lake, Buffalo Lake	MH369270		Present study
Echinostomatidae	*Echinostoma bolschewense*	*Viviparus acerosus*	1	Slovakia: Danube at Gabcikovo		KP065608, KP065621	(Georgieva et al., 2014)
Echinostomatidae	*Echinostoma caproni*	*unknown*		Madagascar; Egypt; Cameroon		AF025837, AF025838	(Morgan & Blair, 1998)
Echinostomatidae	*Echinostoma* cf. friedi	*Planorbis* sp.	1	UK: Wales, Pwll Penarth		AY168937	(Kostadinova & Herniou, 2003)
Echinostomatidae	*Echinostoma deserticum*	unknown		Nigeria: Niger		AF025836	(Morgan & Blair, 1998)
Echinostomatidae	*Echinostoma hortense* (out)	Dog	3	China	KR062182		(Liu et al., 2016)
Echinostomatidae	*Echinostoma miyagawai*	*Anas platyrhynchos*	3	New Zealand: Clutha River System, Central Otago District, South Island		KY436400	(Stoyanov et al., 2017)
Echinostomatidae	*Echinostoma miyagawai*	*Planorbis planorbis, Aythya fuligula*	1, 3	Czech Republic: Pond Louzek; vicinities of Tovacov		KP065632, KP065640	(Georgieva et al., 2014)
Echinostomatidae	*Echinostoma nasincovae*	*Planorbarius corneus*	1	Slovakia: Danube at Gabcikovo; Czech Republic: Pond Hluboky u Hamru		KP065659, KP065674	(Georgieva et al., 2014)
Echinostomatidae	*Echinostoma novazealandense*	*Anas platyrhynchos*	3	New Zealand: Clutha River System, Central Otago District, South Island		KY436398, KY436399	(Stoyanov et al., 2017)
Echinostomatidae	*Echinostoma paraensei*	*Glyptophysa*	1	Brazil; Australia: North Queensland, Townsville		AF025834, AF026282	(Morgan & Blair, 1998)
Echinostomatidae	*Echinostoma paraulum*	*Lymnaea stagnalis, Aythya fuligula*	1, 3	Germany: pond near Poppenwind; Czech Republic: vicinities of Tovacov		KP065677, KP065680	(Georgieva et al., 2014)
Echinostomatidae	*Echinostoma revolutum Lineage A*	*Lymnaea peregra*	1	Bulgaria: Grigorevo		AY168934	(Kostadinova & Herniou, 2003)
Echinostomatidae	*Echinostoma revolutum Lineage A*	*Lymnaea stagnalis, Aythya fuligula*	1, 3	Czech Republic: Pond Vlkovsky; vicinities of Tovacov; Pond Hluboky u Hamru		KP065646, KP065653, KP065658	(Georgieva et al., 2014)
Echinostomatidae	*Echinostoma revolutum Lineage A*	*Domestic duck*	3	Thailand		KP455631, KP455632, KP455633	(Nagataki et al., 2015)
Echinostomatidae	*Echinostoma revolutum Lineage A*	*Columba livia f. domestica*	3	Poland		KT726380	Ledwon, A., et al., 2015, Unpublished
Echinostomatidae	*Echinostoma revolutum Lineage B*	*Stagnicola elodes*	1	Canada: Alberta, Buffalo Lake, Gull Lake, Wabamun Lake, Isle Lake, Lac La Nonne		MH369192, MH369193, MH369194, MH369195, MH369196, MH369197, MH369200, MH369201, MH369202, MH369204, MH369206, MH369207, MH369208, MH369209, MH369210, MH369211, MH369213, MH369214, MH369215, MH369216, MH369217, MH369218, MH369219, MH369220, MH369221, MH369222	Present study
Echinostomatidae	*Echinostoma revolutum Lineage B*	*Lymnaea elodes*	1	USA: Indiana, Pond A		GQ463056, GQ463057	(Detwiler et al., 2010)
Echinostomatidae	*Echinostoma revolutum Lineage B*	*Stagnicola elodes*	1	Canada: Alberta, Buffalo Lake, Gull Lake, Wabamun Lake, Isle Lake, Lac La Nonne	MH369227, MH369229, MH369230, MH369231, MH369235, MH369242, MH369248, MH369268, MH369279, MH369281, MH369284, MH369286, MH369287, MH369292		Present study
Echinostomatidae	*Echinostoma robustum*	*Lymnaea elodes, Biomphalaria glabrata, Gallus gallus*	1, 1, 3	USA: Indiana, Pond A; Minnesota; Brazil		GQ463053^*^, GQ463054, GQ463055	(Detwiler et al., 2010)
Echinostomatidae	*Echinostoma* sp.	*Hydromys chrysogaster*	3	Australia: North Queensland, Townsville		AF026290	(Morgan & Blair, 1998)
Echinostomatidae	*Echinostoma* sp. IG	*Radix auricularia*	1	Germany: Hengsteysee		KC618449, KC618450	(Georgieva, Selbach, et al., 2013)
Echinostomatidae	*Echinostoma* sp. NZ‐Ad^*^	*Branta canadensis*	3	New Zealand		AF026289^*^	(Morgan & Blair, 1998)
Echinostomatidae	*Echinostoma trivolvis*	*Ondatra zibethicus*	3	Canada: Ontario, Lake Opinicon	KM538091		(Van Steenkiste et al., 2014)
Echinostomatidae	*Echinostoma trivolvis Lineage A*	*Helisoma trivolvis*	1	Canada: Alberta, Isle Lake, Wabamun Lake, Lac La Nonne		MH369198, MH369199, MH369203, MH369205, MH369212	Present study
Echinostomatidae	*Echinostoma trivolvis Lineage A*	*Helisoma trivolvis*	1	Canada: Alberta, Isle Lake, Wabamun Lake, Lac La Nonne	MH369271		Present study
Echinostomatidae	*Echinostoma trivolvis Lineage B*	*Ondatra zibethicus, Lymnaea elodes*	3, 1	USA: Wisconsin; Minnesota		GQ463051, GQ463052, GQ463113	(Detwiler et al., 2010)
Echinostomatidae	*Echinostoma trivolvis Lineage B*	*Ondontra zibethicus*	3	USA: Virginia		JQ670857, JQ670859, JQ670850	(Detwiler, Zajac, Minchella, & Belden, 2012)
Echinostomatidae	*Echinostoma trivolvis Lineage B*	*unknown*		North America		AF025831	(Morgan & Blair, 1998)
Echinostomatidae	*Echinostomatidae gen*. sp.^***^	*Stagnicola elodes*	1	Canada: Alberta, Buffalo Lake	MH369269, MH369295, MH369297		Present study
Echinostomatidae	*Euparyphium capitaneum (out)*	*Anhinga anhinga*	3	Mexico: Veracruz, Tecolutla; Nayarit, La Tovara	KY636235, KY636236		(Hernández‐Cruz et al., 2018)
Echinostomatidae	*Fasciola hepatica (out)*	*Cattle*	3	Iran		KT893744	Akhlaghi, E., et al., 2015, Unpublished
Echinostomatidae	*Hypoderaeum conoideum*	*Lymnaea peregra*	1	Bulgaria: Grigorevo		AY168949	(Kostadinova & Herniou, 2003)
Echinostomatidae	*Hypoderaeum conoideum*	*Anas discors*	3	Canada: Manitoba, Lake Manitoba, South shore, Delta Marsh	KM538101		(Van Steenkiste et al., 2014)
Echinostomatidae	*Hypoderaeum* sp. Lineage 1	*Lymnaea elodes*	1	USA: Indiana, Pond A		GQ463100, GQ463101, GQ463102	(Detwiler et al., 2010)
Echinostomatidae	*Hypoderaeum* sp. Lineage 1	*Stagnicola elodes*	1	Canada: Alberta, Gull Lake, Lac La Nonne, Wabamun Lake, Isle Lake		MH368958, MH369020, MH369030, MH369040, MH369080, MH369108, MH369110, MH369145, MH369157	Present study
Echinostomatidae	*Isthmiophora melis (out)*	*Planorbis* sp.	1	UK: Wales, Llyn Mawr		AY168948	(Kostadinova & Herniou, 2003)
Echinostomatidae	*Neopetasiger islandicus*	*Planorbula armigera*	1	Canada: Alberta, Wabamun Lake		KT831342	(Gordy et al., 2016)
Echinostomatidae	*Neopetasiger neocomense*	*Podiceps cristatus*	3	Czech Republic		JQ425591	(Georgieva, Kostadinova, & Skirnisson, 2012)
Echinostomatidae	*Neopetasiger* sp. 1	*Gyraulus albus*	1	Germany: Lake Hennetalsperre		KM191808, KM191809	(Selbach et al., 2014)
Echinostomatidae	*Neopetasiger* sp. 2	*Gyraulus albus*	1	Germany: Lake Hennetalsperre		KM191810, KM191811	(Selbach et al., 2014)
Echinostomatidae	*Neopetasiger* sp. 3	*Planorbis planorbis, Gyraulus albus*	1	Germany: Lake Kleiner Ploener See; Lake Hennetalsperre		KM191814, KM191815, KM191816	(Selbach et al., 2014)
Echinostomatidae	*Neopetasiger* sp. 4	*Gasterosteus aculeatus*	2	Canada: Lake Gosling		KM191817	(Selbach et al., 2014)
Echinostomatidae	*Neopetasiger* sp. 4	*Helisoma trivolvis*	1	Canada: Alberta, Wabamun Lake		KT831343, KT831345	(Gordy et al., 2016)
Echinostomatidae	*Neopetasiger* sp. 4	*Helisoma trivolvis*	1	Canada: Alberta, Wabamun Lake, Isle Lake, Buffalo Lake		MH369311, MH369312, MH369313, MH369314, MH369315, MH369316, MH369317, MH369318	Present study
Haematoloechidae	*Haematoloechidae gen*. sp. A^***^	*Stagnicola elodes*	1	Canada: Alberta, Buffalo Lake	KT831372^§^		(Gordy et al., 2016)
Haematoloechidae	*Haematoloechidae gen*. sp. A^***^	*Stagnicola elodes*	1	Canada: Alberta, Buffalo Lake	MH369319, MH369320, MH369321		Present study
Haematoloechidae	*Haematoloechus* sp.	*Rana pipiens*	3	Canada: Quebec, Outaouais, Ottawa River, Wendover; Ontario, Southern Ontario, Chatham‐Kent, East of Lake St.Clair and St. Clair National Wildlife Area	KM538096, KM538097		(Van Steenkiste et al., 2014)
Notocotylidae	*Notocotylidae gen*. sp. A^***^	*Stagnicola elodes*	1	Canada: Alberta, Gull Lake	KT831348^§^, KT831364		(Gordy et al., 2016)
Notocotylidae	*Notocotylidae gen*. sp. A^***^	*Physa gyrina, Stagnicola elodes*	1	Canada: Alberta, Wabamun Lake, Isle Lake, Gull Lake, Buffalo Lake, Lac La Nonne	MH369323, MH369405, MH369324, MH369406, MH369325, MH369326, MH369327, MH369407, MH369408, MH369328, MH369409, MH369329, MH369330, MH369331, MH369332, MH369333, MH369334, MH369335, MH369336, MH369337, MH369338, MH369339, MH369340, MH369341, MH369342, MH369343, MH369344, MH369345, MH369410, MH369346, MH369347, MH369348, MH369349, MH369350, MH369351, MH369352, MH369411, MH369353, MH369354, MH369355, MH369356, MH369357, MH369358, MH369359, MH369360, MH369361, MH369362, MH369363, MH369364, MH369365, MH369366, MH369367, MH369368, MH369412, MH369369, MH369370, MH369371, MH369372, MH369373, MH369374, MH369375, MH369376, MH369378, MH369413, MH369414, MH369379, MH369380, MH369381, MH369382, MH369383, MH369415, MH369416, MH369384, MH369385, MH369386, MH369387, MH369388, MH369389, MH369390, MH369391, MH369417, MH369392, MH369393, MH369394, MH369395, MH369396, MH369397, MH369398, MH369399, MH369400, MH369401, MH369402, MH369403, MH369404		Present study
Notocotylidae	*Notocotylus* sp.	*Mergus merganser*	3	Canada: Quebec, Hudson, Le Nichoir	KM538104		(Van Steenkiste et al., 2014)
Notocotylidae	*Ogmocotyle* sikae	Unknown		China: Hunan Province, Jishou City	KR006934(NC_027112:6904‐8460)		Ma, J., et al., 2015, Unpublished
Plagorchiidae	*Plagiorchis* sp. Lineage 1	*Stagnicola elodes*	1	Canada: Alberta, Gull Lake, Lac La Nonne, Buffalo Lake	MH369420, MH369421, MH369422, MH369433, MH369434, MH369435, MH369441, MH369460, MH369461, MH369463, MH369464		Present study
Plagorchiidae	*Plagiorchis* sp. Lineage 2	*Stagnicola elodes*	1	Canada: Alberta, Buffalo Lake	MH369467		Present study
Plagorchiidae	*Plagiorchis* sp. Lineage 3	*Stagnicola elodes*	1	Canada: Alberta, Buffalo Lake	MH369442, MH369454, MH369466		Present study
Plagorchiidae	*Plagiorchis* sp. Lineage 4	*Stagnicola elodes*	1	Canada: Alberta, Gull Lake, Lac La Nonne, Buffalo Lake, Wabamun Lake, Isle Lake	MH369418, MH369423, MH369425, MH369428, MH369429, MH369431, MH369432, MH369436, MH369437, MH369440, MH369447, MH369452, MH369453, MH369456, MH369462, MH369471		Present study
Plagorchiidae	*Plagiorchis* sp. Lineage 5	*Stagnicola elodes*	1	Canada: Alberta, Gull Lake, Lac La Nonne	MH369419, MH369426, MH369427		Present study
Plagorchiidae	*Plagiorchis* sp. Lineage 6	*Helisoma trivolvis*	1	Canada: Alberta, Buffalo Lake	MH369470		Present study
Plagorchiidae	*Plagiorchis* sp. Lineage 7	*Lymnaea stagnalis*	1	Canada: Alberta, Buffalo Lake, Gull Lake	MH369438, MH369448, MH369455, MH369458, MH369468, MH369469		Present study
Plagorchiidae	*Plagiorchis* sp. Lineage 8	*Stagnicola elodes*	1	Canada: Alberta, Buffalo Lake, Gull Lake	MH369449, MH369450, MH369451, MH369459, MH369465		Present study
Plagorchiidae	*Plagiorchis* sp. Lineage 9	*Stagnicola elodes*	1	Canada: Alberta, Lac La Nonne, Buffalo Lake	MH369424, MH369430, MH369439, MH369443, MH369444, MH369445, MH369446		Present study
Plagorchiidae	*Plagiorchis* sp.	*Larus delawarensis*	3	Canada: Quebec, St. Lawrence River	FJ477214		(Moszczynska et al., 2009)
Psilostomidae	*Echinochasmus japonicus (out)*	*Homo sapiens*	3	Viet Nam: Phu Tho	NC_030518		Le, T.H., et al., 2015, Unpublished
Psilostomidae	*Pseudopsilostoma varium*	*Phalacrocorax auritus*	3	USA: Mississippi	JX468064		(O'Hear et al., 2014)
Psilostomidae	*Psilostomatidae gen*. sp. A^***^	*Helisoma trivolvis*	1	Canada: Alberta, Wabamun Lake	MH369477^§^		(Gordy et al., 2016)
Psilostomidae	*Psilostomatidae gen*. sp. A^***^	*Helisoma trivolvis*	1	Canada: Alberta, Wabamun Lake, Isle Lake	MH369473, MH369472, MH369476, MH369474, MH369475		Present study
Psilostomidae	*Sphaeridiotrema globulus*	Duck experimentally infected with metacercariae from Elimia virginica	2, 3	USA: Lake Musconetcong, New Jersey	GQ890329		(Bergmame et al., 2011)
Psilostomidae	*Sphaeridiotrema pseudoglobulus*	Duck experimentally infected with metacercariae from Bithynia tentaculata	2, 3	Canada: Riviere du Sud, Quebec	GQ890328		(Bergmame et al., 2011)
Psilostomidae	*Sphaeridiotrema pseudoglobulus*	*Aythya affinis*	3	Canada: Quebec, St. Lawrence River	FJ477222		(Moszczynska et al., 2009)
Strigeidae	*Apatemon* sp. 1	*Etheostoma nigrum*	2	Canada: Quebec, St. Lawrence River, Lake St. Louis	FJ477183		(Moszczynska et al., 2009)
Strigeidae	*Apatemon* sp. 1	*Etheostoma nigrum*	2	Canada: Ontario, St. Lawrence River, Lake Saint Francois	HM064633		(Locke, McLaughlin, & Marcogliese, 2010)
Strigeidae	*Apatemon* sp. 1x	*Etheostoma nigrum*	2	Canada: Ontario, St. Lawrence River, Lake Saint Francois	HM064635, HM064636		(Locke, McLaughlin, & Marcogliese, 2010)
Strigeidae	*Apatemon* sp. 3	*Ambloplites rupestris*	2	Canada: Quebec, St. Lawrence River, Lake St. Pierre, Iles aux Sables	FJ477185		(Moszczynska et al., 2009)
Strigeidae	*Apatemon* sp. 3	*Ambloplites rupestris*	2	Canada: Quebec, St. Lawrence River, Lake St. Pierre, Iles aux Sables	HM064645		(Locke, McLaughlin, & Marcogliese, 2010)
Strigeidae	*Apatemon* sp. 4	*Ambloplites rupestris*	2	Canada: Quebec, St. Lawrence River, Lake Saint Francois	FJ477186		(Moszczynska et al., 2009)
Strigeidae	*Apatemon* sp. 4	*Ambloplites rupestris*	2	Canada: Quebec, St. Lawrence River, Lake St. Pierre, Iles aux Sables	HM064647		(Locke, McLaughlin, & Marcogliese, 2010)
Strigeidae	*Apatemon* sp. A^***^	*Stagnicola elodes*	1	Canada: Alberta, Isle Lake	MH369603, MH369604, MH369605, MH369606, MH369607, MH369608, MH369609, MH369610, MH369611, MH369612, MH369613, MH369614, MH369615, MH369616, MH369617		Present study
Strigeidae	*Apatemon* sp. B^***^	*Stagnicola elodes*	1	Canada: Alberta, Isle Lake	MH369618		Present study
Strigeidae	*Apatemon* sp. C^***^	*Stagnicola elodes*	1	Canada: Alberta, Isle Lake	MH369619, MH369620, MH369621, MH369622		Present study
Strigeidae	*Apatemon* sp. ‘jamiesoni’	*Potamopyrgus antipodarum, Gobiomorphus cotidianus*	1, 2	New Zealand	KT334181, KT334182		(Blasco‐Costa et al., 2016)
Strigeidae	*Apharyngostrigea pipientis* (out)	*Lithobates pipiens*	2	Canada: Quebec, Monteregie, Boucherville, Etang Saulaie	HM064884, HM064885		(Locke et al., 2011)
Strigeidae	*Apharynogstrigea cornu*	*Ardea alba*	3	Mexico: Veracruz, Panuco	JX977777		(Hernández‐Mena, García‐Prieto, & García‐Varela, 2014)
Strigeidae	*Apharynogstrigea cornu*	*Ardea herodias*	3	Canada: Quebec, St. Lawrence River, Lake St. Louis, Ile aux Herons	JF769451		(Locke et al., 2011)
Strigeidae	*Australapatemon burti LIN1*	*Stagnicola elodes*	1	Canada: Alberta, Isle Lake	KT831346, KT831351		(Gordy et al., 2016)
Strigeidae	*Australapatemon burti LIN1*	*Stagnicola elodes, Physa gyrina, Helisoma trivolvis, Helisoma campanulatum, Planorbis sp., Lymnaea stagnalis*	1	Canada: Alberta, Isle Lake, Wabamun Lake, Lac La Nonne, Gull Lake, Buffalo Lake	KY207548, KY207549, KY207551, KY207552, KY207553, KY207554, KY207555, KY207556, KY207559, KY207560, KY207561, KY207562, KY207563, KY207564, KY207565, KY207566, KY207567, KY207568, KY207570, KY207571, KY207572, KY207573, KY207574, KY207575, KY207576, KY207578, KY207579, KY207580, KY207581, KY207584, KY207585, KY207586, KY207588, KY207589, KY207590, KY207591, KY207592, KY207593, KY207594, KY207595, KY207598, KY207599, KY207600, KY207601, KY207602, KY207603, KY207604, KY207605, KY207606, KY207607, KY207608, KY207609, KY207610, KY207611, KY207612, KY207614, KY207617, KY207618, KY207619, KY207620, KY207621, KY207623, KY207624, KY587399, KY587398, KY587394, KY587401, HM385485, KY587400, HM385486		(Gordy, Locke, Rawlings, Lapierre, & Hanington, 2017)
Strigeidae	*Australapatemon burti LIN1*	*Stagnicola elodes, Physa gyrina, Helisoma trivolvis*	1	Canada: Alberta, Isle Lake, Wabamun Lake, Lac La Nonne, Gull Lake, Buffalo Lake	MH369623, MH369624, MH369625, MH369626, MH369627, MH369628, MH369629, MH369630, MH369631, MH369632, MH369633, MH369634, MH369635, MH369636, MH369637, MH369638, MH369639, MH369640, MH369641, MH369642, MH369643, MH369644, MH369645, MH369646, MH369647, MH369648, MH369649, MH369650, MH369651, MH369652, MH369653, MH369654, MH369655, MH369656, MH369657, MH369658, MH369659, MH369660, MH369661, MH369662, MH369663, MH369664, MH369665, MH369666, MH369667, MH369668, MH369669, MH369670, MH369671, MH369672, MH369673, MH369674, MH369675, MH369676, MH369677, MH369678, MH369679, MH369680, MH369681, MH369682, MH369683, MH369684, MH369686, MH369687, MH369688, MH369689, MH369690, MH369691, MH369692, MH369693, MH369694, MH369695, MH369696, MH369697, MH369698, MH369699, MH369700, MH369701, MH369702, MH369703, MH369704, MH369705, MH369706, MH369707, MH369708, MH369709, MH369710, MH369711, MH369712, MH369713, MH369714, MH369715, MH369716, MH369717, MH369718, MH369719, MH369720, MH369721, MH369722, MH369723, MH369724, MH369725, MH369726, MH369727, MH369728, MH369729, MH369730, MH369731, MH369732, MH369733, MH369734, MH369735, MH369736, MH369737, MH369738, MH369739, MH369740, MH369741, MH369742, MH369743, MH369744, MH369745, MH369746, MH369747, MH369748, MH369749, MH369750, MH369751, MH369752, MH369753, MH369754, MH369755, MH369756, MH369757, MH369758, MH369759, MH369760, MH369761, MH369762, MH369763, MH369685		Present study
Strigeidae	*Australapatemon mclaughlini*	*Anas americana*	3	Mexico: Baja California Sur, Guerrero Negro	JX977725		(Hernández‐Mena et al., 2014)
Strigeidae	*Australapatemon mclaughlini*	*Physa gyrina, Anas acuta*	1, 3	Canada: Alberta, Buffalo Lake; Ontario	KY207615, KY207627, KY207628		(Gordy et al., 2017)
Strigeidae	*Australapatemon mclaughlini*	*Physa gyrina*	1	Canada: Alberta, Buffalo Lake	MH369764		Present study
Strigeidae	*Australapatemon niewiadomski*	*Barbronia weberi, Anas platyrhynchos*	2, 3	New Zealand	KT334176, KT334177, KT334178, KT334179, KT334180		(Blasco‐Costa et al., 2016)
Strigeidae	*Australapatemon* sp. LIN2	*Bucephala albeola*	3	Canada: Ontario	HM385535		(Gordy et al., 2017)
Strigeidae	*Australapatemon* sp. LIN3	*Stagnicola elodes*	1	Canada: Alberta, Gull Lake	KY207577		(Gordy et al., 2017)
Strigeidae	*Australapatemon* sp. LIN4	*Physa gyrina, Aythya collaris*	1, 3	Canada: Alberta, Lac La Nonne; Ontario	KY207569, KY587397, KY587396		(Gordy et al., 2017)
Strigeidae	*Australapatemon* sp. LIN4	*Physa gyrina*	1	Canada: Alberta, Gull Lake	MH369765		Present study
Strigeidae	*Australapatemon* sp. LIN5	*Stagnicola elodes*	1	Canada: Alberta, Buffalo Lake	KY207597		(Gordy et al., 2017)
Strigeidae	*Australapatemon* sp. LIN6	*Anas cyanoptera*	3	Mexico: Estado de Mexico	JX977726		(Hernández‐Mena et al., 2014)
Strigeidae	*Australapatemon* sp. LIN6	*Physa gyrina*	1	Canada: Alberta, Pigeon Lake, Isle Lake	KY207613, KY207616		(Gordy et al., 2017)
Strigeidae	*Australapatemon* sp. LIN6	*Physa gyrina*	1	Canada: Alberta, Isle Lake, Buffalo Lake, Lac La Nonne	MH369766, MH369767, MH369768, MH369769, MH369770		Present study
Strigeidae	*Australapatemon* sp. LIN8	*Oxyura jamaicensis*	3	Mexico: Durango, Guatimape	JX977728		(Hernández‐Mena et al., 2014)
Strigeidae	*Australapatemon* sp. LIN8	*Physa gyrina, Oxyura jamaicensis*	1, 3	Canada: Alberta, Isle Lake, Buffalo Lake; Ontario	KY207587, KY207622, HM385538, HM385537, HM385536		(Gordy et al., 2017)
Strigeidae	*Australapatemon* sp. LIN8	*Physa gyrina*	1	Canada: Alberta, Isle Lake, Buffalo Lake, Gull Lake	MH369771, MH369772, MH369773, MH369774, MH369775, MH369776, MH369777		Present study
Strigeidae	*Australapatemon* sp. LIN9A	*Stagnicola elodes, Anas acuta*	1, 3	Canada: Alberta, Gull Lake, Isle Lake, Buffalo Lake; Ontario	KY207550^§^, KY207557^§^, KY207558^§^, KY207582^§^, KY207596^§^, HM385534^§^		(Gordy et al., 2017)
Strigeidae	*Australapatemon* sp. LIN9A	*Stagnicola elodes, Lymnaea stagnalis (MGC176B)*	1	Canada: Alberta, Lac La None, Gull Lake, Buffalo Lake, Isle Lake	MH369779, MH369780, MH369781, MH369782, MH369783, MH369784, MH369785, MH369786, MH369787, MH369788, MH369789, MH369778		Present study
Strigeidae	*Australapatemon* sp. LIN9B	*Stagnicola elodes*	1	Canada: Alberta, Buffalo Lake	KY207583^§^		(Gordy et al., 2017)
Strigeidae	*Australapatemon* sp. LIN9B	*Stagnicola elodes*	1	Canada: Alberta, Buffalo Lake	MH369790, MH369791, MH369792		Present study
Strigeidae	*Australapatemon* sp. LIN10^***^	*Stagnicola elodes*	1	Canada: Alberta, Gull Lake	MH369793		Present study
Strigeidae	*Cardiocephaloides medioconiger*	*Larus* sp.	3	Mexico: Laguna de Términos, Campeche	JX977782, JX977783		(Hernández‐Mena et al., 2014)
Strigeidae	*Cardiocephaloides* sp.	*Larus occidentalis*	3	Mexico: Baja California Sur, Guerrero Negro	JX977784		(Hernández‐Mena et al., 2014)
Strigeidae	*Cotylurus cornutus*	*Radix balthica, Gyraulus acronicus*	1	Norway: Lake Takvatn	KY513231, KY513232, KY513233, KY513234, KY513235, KY513236		(Soldánová et al., 2017)
Strigeidae	*Cotylurus cornutus*	*Stagnicola elodes*	1	Canada: Alberta, Gull Lake	KT831347^§^		(Gordy et al., 2017)
Strigeidae	*Cotylurus cornutus*	*Stagnicola elodes, Helisoma trivolvis (MGC205)*	1	Canada: Alberta, Gull Lake, Isle Lake, Lac La Nonne	MH369478, MH369480, MH369484, MH369485, MH369486, MH369487, MH369488, MH369489, MH369490, MH369491, MH369492, MH369493, MH369494, MH369495, MH369496, MH369497, MH369498, MH369500, MH369501, MH369502, MH369503, MH369504, MH369505, MH369509, MH369510, MH369511, MH369516, MH369532, MH369538, MH369539, MH369544, MH369557, MH369597, MH369601		Present study
Strigeidae	*Cotylurus gallinulae*	*Aythya affinis*	3	Mexico: Sonora, La esperanza	JX977781		(Hernández‐Mena et al., 2014)
Strigeidae	*Cotylurus gallinulae*	*Physa gyrina*	1	Canada: Alberta, Buffalo Lake, Wabamun Lake, Isle Lake, Lac La Nonne	MH369517, MH369518, MH369525, MH369526, MH369527, MH369528, MH369529, MH369560, MH369571, MH369572, MH369574, MH369575, MH369577, MH369583, MH369584, MH369587, MH369588, MH369590, MH369595, MH369596, MH369599, MH369600		Present study
Strigeidae	*Cotylurus* sp. A^***^	*Stagnicola elodes*	1	Canada: Alberta, Isle Lake	KT831371^§^		(Gordy et al., 2016)
Strigeidae	*Cotylurus* sp. A^***^	*Stagnicola elodes, Physa gyrina (MGC1962)*	1	Canada: Alberta, Isle Lake, Lac La Nonne, Wabamun	MH369513, MH369520, MH369521, MH369522, MH369523, MH369524, MH369533, MH369537, MH369541, MH369542, MH369543, MH369545, MH369546, MH369547, MH369548, MH369549, MH369550, MH369551, MH369552, MH369554, MH369555, MH369556, MH369558, MH369559, MH369561, MH369562, MH369573, MH369578, MH369579, MH369580, MH369581, MH369582, MH369585, MH369589, MH369591, MH369594, MH369598, MH369602		Present study
Strigeidae	*Cotylurus* sp. B^***^	*Physa gyrina*	1	Canada: Alberta, Isle Lake	MH369586		Present study
Strigeidae	*Cotylurus* sp. C^***^	*Stagnicola elodes*	1	Canada: Alberta, Buffalo Lake, Gull Lake, Isle Lake, Lac La Nonne	MH369479, MH369481, MH369515, MH369530, MH369531, MH369553, MH369564		Present study
Strigeidae	*Cotylurus* sp. D^***^	*Stagnicola elodes*	1	Canada: Alberta, Buffalo Lake, Gull Lake, Isle Lake, Lac La Nonne	MH369482, MH369483, MH369499, MH369506, MH369507, MH369508, MH369534, MH369535, MH369536, MH369540, MH369565, MH369593		Present study
Strigeidae	*Cotylurus* sp. E^***^	*Lymnaea stagnalis*	1	Canada: Alberta, Buffalo Lake, Wabamun Lake	MH369512, MH369514, MH369567, MH369568, MH369569, MH369570		Present study
Strigeidae	*Cotylurus* sp. F^***^	*Stagnicola elodes*	1	Canada: Alberta, Isle Lake	MH369519		Present study
Strigeidae	*Cotylurus* sp. G^***^	*Lymnaea stagnalis*	1	Canada: Alberta, Buffalo Lake	MH369563, MH369566, MH369576		Present study
Strigeidae	*Cotylurus* sp. H^***^	*Physa gyrina*	1	Canada: Alberta, Buffalo Lake	MH369592		Present study
Strigeidae	*Ichthyocotylurus pileatus*	*Perca flavescens, Etheostoma nigrum*	2	Canada: Quebec, St. Lawrence River, Lake Saint Louis, Beauharnois	HM064721, HM064726		(Locke, McLaughlin, & Marcogliese, 2010)
Strigeidae	*Ichthyocotylurus pileatus*	*Perca flavescens*	2	Canada: Ontario, St. Lawrence River, Lake Saint Francois	FJ477204		(Locke, McLaughlin, & Marcogliese, 2010)
Strigeidae	*Ichthyocotylurus* sp. 2	*Perca flavescens*	2	Canada: Quebec, St. Lawrence River, Lake Saint Louis, Beauharnois	HM064728		(Locke, McLaughlin, & Marcogliese, 2010)
Strigeidae	*Ichthyocotylurus* sp. 3	*Notropis hudsonius*	2	Canada: Ontario, St. Lawrence River, Lake Saint Francois	HM064729		(Locke, McLaughlin, & Marcogliese, 2010)
Strigeidae	*Tylodelphys scheuringi* (out)	*Ambloplites rupestris*	2	Canada: Quebec, St. Lawrence River, Lake Saint Pierre, Iles aux Sables	FJ477223		(Moszczynska et al., 2009)

*** Novel by molecular phylogeny; § Record updated in present study; Host Type: 1= First Intermediate, 2 = Second Intermediate, 3 = Definitive

* Most likely Posthodiplostomum centrarchi

Rows highlighted in gray represent sequences from the present study

**Table 4:** Snail host–trematode parasite relationships from this study

	*Helisoma trivolvis*	*Lymnaea stagnalis*	*Physa gyrina*	*Planorbula armigera*	*Stagnicola elodes*	Grand total
*Apatemon* sp. A	–	–	–	–	16	16
*Apatemon* sp. B	–	–	–	–	1	1
*Apatemon *sp. C	–	–	–	–	4	4
*Australapatemon burti* LIN1	2	1	2	–	199	204
*Australapatemon mclaughlini*	–	–	2	–	–	2
*Australapatemon* sp. LIN10	–	–	1	–	–	1
*Australapatemon* sp. LIN3	–	–	–	–	1	1
*Australapatemon* sp. LIN4	–	–	2	–	–	2
*Australapatemon* sp. LIN5	–	–	–	–	1	1
*Australapatemon* sp. LIN6	–	–	7	–	–	7
*Australapatemon* sp. LIN8	–	–	9	–	–	9
*Australapatemon* sp. LIN9A	–	1	–	–	16	17
*Australapatemon* sp. LIN9B	–	–	–	–	4	4
Avian schistosomatid sp. A	–	–	7	–	–	7
Avian schistosomatid sp. B	–	–	1	–	–	1
Avian schistosomatid sp. C	1	–	–	–	–	1
*Bolbophorus* sp.	10	–	–	–	–	10
*Cotylurus cornutus*	1	–	–	–	32	33
*Cotylurus flabelliformis*	–	–	–	–	1	1
*Cotylurus marcogliesei*	–	–	–	–	5	5
*Cotylurus* sp. A	–	–	1	–	38	39
*Cotylurus* sp. B	–	–	1	–	–	1
*Cotylurus* sp. C	–	3	–	–	–	3
*Cotylurus* sp. D	–	–	1	–	–	1
*Cotylurus* sp. E	–	–	–	–	11	11
*Cotylurus* sp. F	–	6	–	–	–	6
*Cotylurus strigeoides*	–	–	21	–	1	22
Diplostomidae gen. sp. O	–	–	34	–	–	34
Diplostomidae gen. sp. X	–	–	1	–	–	1
*Diplostomum baeri* LIN2	–	–	–	–	5	5
*Diplostomum indistinctum*	–	–	–	–	1	1
*Diplostomum* sp. 1	–	–	–	–	6	6
*Diplostomum* sp. 3	–	3	–	–	–	3
*Diplostomum* sp. 4	–	–	–	–	71	71
*Diplostomum* sp. A	–	–	–	–	1	1
*Diplostomum* sp. B	–	–	–	–	1	1
*Diplostomum* sp. C	1	–	–	–	11	12
*Drepanocephalus spathans*	4	–	–	–	–	4
*Echinoparyphium* sp. A	–	–	44	–	2	46
*Echinoparyphium* sp. A2	–	–	1	–	6	7
*Echinoparyphium* sp. B	–	–	–	–	9	9
*Echinoparyphium* sp. C	–	–	–	–	25	25
*Echinoparyphium* sp. D	–	–	–	–	1	1
*Echinoparyphium* sp. E	–	1	–	–	5	6
*Echinoparyphium* sp. Lineage 1A	1	–	94	–	2	97
*Echinoparyphium* sp. Lineage 1B	–	–	1	–	–	1
*Echinoparyphium* sp. Lineage 2	1	3	–	–	80	84
*Echinoparyphium* sp. Lineage 4	2	–	–	–	–	2
*Echinostoma revolutum* B	–	–	–	–	33	33
*Echinostoma trivolvis* Lineage A	5	–	–	–	–	5
Echinostomatidae gen. sp.	–	–	–	–	4	4
Haematoloechidae gen. sp. A	–	–	–	–	4	4
*Hypoderaeum* sp. Lineage 1	–	–	–	–	5	5
*Hypoderaeum* sp. Lineage 2	–	–	–	–	3	3
*Neodiplostomum americanum*	–	–	–	–	1	1
*Neopetasiger islandicus*	–	–	–	1	–	1
*Neopetasiger* sp. 4	10	–	–	–	–	10
*Notocotylus* sp. A	–	–	36	–	3	39
*Notocotylus* sp. B	–	–	1	–	–	1
*Notocotylus* sp. C	1	–	–	–	–	1
*Notocotylus* sp. D	–	–	5	–	45	50
*Ornithodiplostomum* sp. 2	–	–	1	–	–	1
*Ornithodiplostomum *sp. 8	–	–	4	–	–	4
*Plagiorchis* sp.^a^	7	89	12	–	1,027	1,135
*Posthodiplostomum *sp. 4	–	–	2	–	–	2
Psilostomidae gen. sp. A	6	–	–	–	–	6
*Schistosomatium douthitti*	–	8	–	–	2	10
*Trichobilharzia physellae*	–	–	1	–	–	1
*Trichobilharzia stagnicolae*	–	–	–	–	8	8
*Trichobilharzia szidati*	–	2	–	–	–	2
*Tylodelphys* sp. A	5	–	–	–	–	5
Grand total	57	117	292	1	1,691	2,158

a Includes all lineages.

Of the 79 total trematode species reported here, 59 are newly identified species in this report that have resulted in 15 updated identifications for previously published sequences (Table 3). Thirty‐nine of the 59 new identifications represent novel lineages/singletons (represented by “a” in Table 3), with another two lineages that represent a recent split (*Australapatemon* sp. LIN9A/9B). The remaining 20 species were previously identified, and for 15 of them, we have added further sequenced specimens, confirming their previous identifications and adding to our understanding of species presence and abundances in Alberta lakes (Table 3 and Appendix: Table A14).

**Figure 10:**
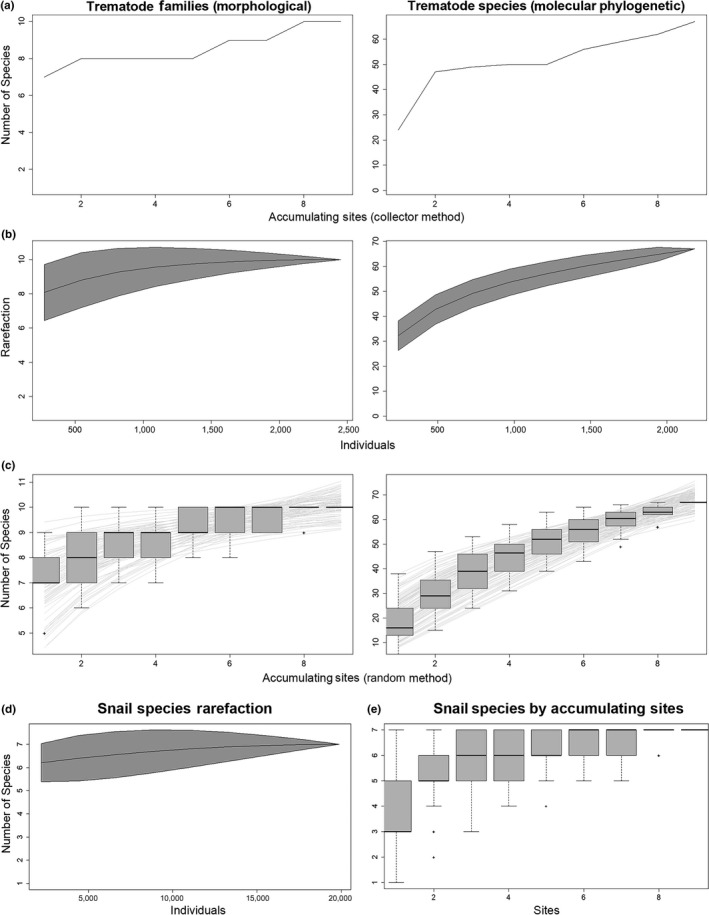
Species accumulation. Several methods confirm that if delineation confidence for species differences is based on morphological identification, a plateau is reached for the maximum number of species within the study area. Whereas if confidence is based on molecular phylogenetic methods, we have yet to attain the true diversity of trematode species within the study area. (a) Collector method for accumulating sites as in the dataset, (b) Rarefaction for number of individuals, (c) Random method for accumulating sites given as a boxplot, with nonlinear Arrhenius model results displayed as lines behind the boxplot, (d) Rarefaction for number of individuals by snail species, (e) Random method for accumulating sites given as a boxplot for snail species

Examining the relationship of trematode species richness and sample size (by sites/area and individuals) through rarefaction and nonlinear models revealed a stark contrast between whether confidence for delimitation was at a family‐level (morphological analysis) or a species‐level (molecular analysis; Figure 10a–c). Considering the accumulation of trematode families, the curves plateaued (individual‐based) or approached one (site‐based), suggesting we likely captured the available trematode families within our samples and sample region. However, when looking at the curves based on trematode species accumulation, there was no plateau, suggesting that there was potentially greater trematode species diversity than we captured from our sampling. Snails, on the other hand, plateaued in rarefaction analyses (Figure 10d,e). This was not at all surprising, considering that over 3 years of collections, we had yet to find more than seven species.

The greatest richness recoveries by snail host species were found among *S. elodes *(40 trematode species), followed by *P. gyrina *(26), *H. trivolvis *(15), *L. stagnalis *(10), and *P. armigera *(1), following the same trend as identified previously (Gordy et al., 2013a), but with more total species (Table 5). Specificity for snail host species was high (55 specialist trematode species) among all but 15 trematodes, of which were found to infect two or more snail species (generalists). Some trematodes were found infecting snails from completely different families, these were *A. burti *LIN1 (*S. elodes, L. stagnalis, H. trivolvis, P. gyrina*), *Echinoparyphium *sp. LIN1A (*S. elodes, H. trivolvis, P. gyrina*), and *Notocotylus *sp. A/D (*S. elodes, P. gyrina*).

**Table 5:** Summary of representation for digenean mitochondrial genes in GenBank

Order	Family	No. of *cox1* seq.	No. of spp.	Named spp.	No. of *nad1* seq.	No. of spp.	Named spp.
Strigeidida	Schistosomatidae	1,434	104	46	523	16	15
	Diplostomidae	1,079	110	20	3	3	
	Clinostimidae	390	29	11	1	1	
	Strigeidae	188	46	18	20	7	
	Bucephalidae	66	5				
	Leuchochloridae	31	6		11	4	
	Bolbophoridae	15	5				
	Aporocotylidae	10	10				
	Cyathocotylidae	9	1				
	Spirorchiidae	5	5				
	Fellodistomidae	5	3		12	11	
	Leuchochloridiomorphidae	1	1				
	Panopstidae	1	1				
Plagiorchiida	Fasciolidae	463	31	4	533	45	7
	Opecoelidae	416	24	7	203	2	2
	Troglotrematidae	333	24	7	35	6	
	Apocreadiidae	261	5	4			
	Echinostomatidae	228	23	10	344	51	28
	Microphallidae	158	6				
	Allocreadiiidae	143	13				
	Dicrocoeliidae	115	15		33	6	
	Himasthlidae	79	9				
	Monorchiidae	69	17				
	Plagiorchiidae	64	19	16			
	Gorgoderidae	61	17				
	Haematoloechidae	52	23	15			
	Paramphistomidae	49	14		34	5	
	Notocotylidae	42	8	2	2	2	
	Gymnophallidae	41	3				
	Brachycladiidae	39	3				
	Lepocreadiidae	39	1		46	40	
	Philophthalmidae	38	5		20	3	
	Collyriclidae	37	1		8	1	
	Gastrothylacidae	23	6		2	2	
	Renicolidae	21	8		6	4	
	Pleurogenidae	15	2				
	Prosthogonimidae	14	4		16	4	
	Gorgocephalidae	8	1				
	Psilostomidae	5	4	3			
	Callodistomidae	4	4				
	Paramphistomatidae	3	3				
	Telorchiidae	2	2				
	Alloglossidiidae	2	1		177	23	17
	Cephalogonimidae	2	1				
	Lissorchiidae	2	2				
	Echinochasmidae	1	1		1	1	
	Olveriidae	1	1				
Opisthorchiida	Heterophyidae	680	29	20	3	2	
	Opisthorchiidae	469	13	12	28	8	
	Cryptogonimidae	66	3		1	1	
	Acanthocolpidae	1	1		1	1	
Azygiida	Didymozoidae	82	2				
	Derogenidae	14	5				
	Accacoeliidae	5	2				
	Azygiidae	3	3				
	Hirudinellidae	2	2				
	Hemiuridae	1	1				
	Isoparorchiidae	1	1				

## 4 DISCUSSION

Fine‐scale molecular analyses of trematodes in central Alberta have revealed many new and important insights about their diversity. What is perhaps most surprising is that species accumulation curves would suggest we have yet to capture all the possible trematode species within our sample area. Comparing the species‐level to family‐level accumulation clearly demonstrates how important the molecular phylogenetic perspective is. Herein, we have used the family‐level as a proxy for the type of results achieved by morphological analysis only, in considering trematode larval stages. While morphological identification of trematode larvae can be less costly in terms of materials, it does not afford a very high level of confidence because of the issues surrounding cryptic species and underdeveloped, definable features. Family‐level accumulation based on individuals and sites is achieved at a much higher rate than species, as expected, and reaches a plateau earlier. If, for instance, this representation is true of the number of species attained by a typical survey, it is likely that trematode surveys are missing much of the actual diversity present. This is important to note because of the potential impact on how we might interpret community assembly and structure in natural environments, especially in consideration of cryptic species.

Overall, the trematode species richness found by this longitudinal survey exceeded expectations, and the number of snail species needed in a community to maintain a diverse set of trematodes was surprisingly small. In our original morphological assessments, we expected 29 trematode species. With the use of molecular assessments, based on BLAST identities and fewer sequenced samples, generated from the first 2 years of the study, we had expanded our view to 39 identified species (Gordy et al., 2013a). Now, with more available sequence information, and the use of more stringent methods, we have, in total, recovered double the species from previous assessments at a total of 79 trematode species, 55 of which are new records to Alberta from this study alone. This raises the recorded trematode species in Alberta to 114, representing 16 families (Appendix: Table A14).

For an ecoregion that has previously been considered species‐poor (Hoberg, Galbreath, Cook, Kutz, & Polley, 1993b), sub‐Arctic lake ecosystems have presented a surprising amount of trematode diversity from recent surveys. From one lake in Norway, on the 69th parallel, 24 different trematode species were recovered, representing seven different, common families from lakes in the Northern hemisphere (e.g., Strigeidae, Diplostomidae, Schistosomatidae, Echinostomatidae, Notocotylidae, Plagiorchiidae; Soldánová et al., 2014). Though further South, between the 54th and 52nd parallel, our study is still considered within the sub‐Arctic region and has uncovered a range of 3–38 trematode species representing 3–8 families, each, among six lakes (the lower end, from Pigeon Lake and Lac La Nonne, was only sampled in 1 year as opposed to 3 years for the other lakes). In between, sampling of fish from the Saint Lawrence River in Quebec (between the 49th and 44th parallel) has revealed 47 species of just diplostomoids (Locke, Mclaughlin et al., 2010). From these surveys, it is apparent that our perspective of what constitutes incredible or unexpected diversity is changing and will continue to change as we take a closer look with molecular data. In all three of these studies, the unveiling of cryptic diversity has been a large component. From a recent meta‐analysis of 110 studies, it has been noted that there is a trend, particularly among trematodes, that sequencing effort positively correlates with more cryptic species as opposed to any other group of helminths. This has been attributed to differences in trematode biology, and our ability as taxonomists to identify them by their morphological characters, or lack thereof (Pérez‐Ponce de León & Poulin, 2010b).

From a basic search of the GenBank database, we can see that trematodes are not a neglected group, as there are 877,472 molecular records specific to digeneans (as of August 2018). However, this is not to say that specific groups of digenean are not neglected nor that representation is not highly skewed to particular gene regions or to those species most important to human or veterinary health. Of the digenean sequences in GenBank, 15,185 were of mitochondrial origin. Considering the two most used mitochondrial genes for barcoding digeneans, we limited our search to *cox1 *and *nad1 *(ND1), finding that a few families were represented by more than 400 sequences, some having more *nad1* than *cox1* or vice versa, and this was not consistent with the estimated number of genera or species within the family. For instance, the family Fasciolidae was found to have 463 *cox1* sequences that represented 31 unnamed species (uniquely identified in GenBank) and four named species. This family was also represented by 533 *nad1* sequences representing 45 molecular species and seven named species. Considering that previous assessments have only identified eight potential species in this family (Cribb et al., 2016), this is incredible coverage. Other families, though, have nearly 900 species, like the Opecoelidae (Bray, Cribb, Littlewood, & Waeschenbach, 2017), and have a similar breadth of species and sequences as the Fasciolidae, showing them to be greatly underrepresented (Table 5).

In this study, the relevant trematode families with the best *cox1/nad1* coverage from GenBank were the Echinostomatidae, Strigeidae, and Diplostomidae. Despite many genera being represented within these families, there remain many gaps in species identifications. This was apparent through a large variety of unidentified species lineages. Unfortunately, our study has only widened this gap, by identifying even more novel, unidentified species lineages and singletons because we lack molecular evidence from adult worms. However, these efforts are not in vain, as they provide a foundation for further sampling that may create the missing life cycle links between larvae and adults in the future. For instance, the species *C. marcogliesei* was just described for the first time this past year (Locke et al., 2018), based on adult worms derived from a Hooded Merganser in Montreal, QC. The alignment of our sequences to that of Locke et al. have now added a new snail first‐intermediate host record, *S. elodes,* in addition to a new geographical record of being in Alberta. Considering that *Cotylurus *spp. have been described as having snails as a second‐intermediate host, it is possible for them to use the same species, although typically not the exact same snail individual (Graczyk & Shiff, 2014). Meaning that further sampling of *S. elodes* may uncover metacercariae of *C. marcogliesei*. Overall, there is further opportunity for this species’ second‐intermediate host to be discovered to complete our understanding of the life cycle and host use within.

The trematode families found in Alberta that need greater sampling and effort from both adult worms and molecular barcoding are the Notocotylidae, Psilostomidae, Haematoloechidae, and most importantly, the Plagiorchiidae. The Plagiorchiidae are the most abundant family found in central Alberta lakes, and there is statistical evidence, through phylogenetics presented herein, for the presence of at least nine species. This family is said to be composed of at least 100 species (Blankespoor, 2013). Furthermore, *Plagiorchis *spp. have been indicated as vectors for Potomac Horse Fever (Vaughan, Tkach, & Greiman, 2016), which has been diagnosed among several horses near Edmonton, Alberta (personal communication with horse owners, and positive sequence identifications of *Neorickettsia risticii*, unpublished).

In the Notocotylidae, we identified four species, but all were provisionally named species A–D because, as with many of our samples, there was no clear evidence to connect them to any previously identified species, and the evidence found was quite disparate. From the literature, only two named species have been identified in Canada, including *Notocotylus attenuatus *(Quebec and Manitoba) and *N. urbanensis* (previously *N. filamentis*) (British Columbia and Ontario), and three others have been identified in the Nearctic region, *N. linearis, N. pacifier, *and *N. stagnicolae*. Broadly, these species infect Anatids and aquatic mammals like muskrats (multiple references found in Gibson, Bray, & Harris, 2016). Prior evidence related to their snail hosts is limited to records from the United States: *N. attenuatus* has been identified from *Physa acuta* in the Eastern United States (Graczyk & Shiff, 2005), and *N. urbanensis* was identified from *Stagnicola emarginata* in Michigan (Keas & Blankespoor, 2017). No records to our knowledge have thus far indicated *P. gyrina, S. elodes, *or *H. trivolvis* as intermediate hosts for *Notocotylus *species. The only records of any *Notocotylus *spp. in Alberta previously have been unnamed species found in the shorebirds *Recurvirostra americana* and *Catoptrophorus semipalmatus *(Gibson et al., 2016). Considering that we cannot link these unknown species in shorebirds to our samples, the four species we have identified can be considered new geographical and host records for Notocotylids. A final note about this family is the need for further sampling among *Stagnicola* and *Physa* snail species in Alberta as an effort to further define *Notocotylus *sp. A and D. These two species have lower interspecific divergence between them than between the other species in the family and exhibit mixed host use, with preference for one host over the other and that happen to be opposite of each other. We speculate that this may be evidence of a current speciation event in which increased host preferences are leading to specialization and resulting in their division, at least on a molecular level.

Both Haematoloechidae and Psilostomidae species were difficult to identify for several reasons. The first reason was that either there were not very many *cox1* sequences available for comparison or the sequences available for that gene were from an upstream region and did not overlap. The other reason was that there have been no previous records of species from either family in Alberta or many records in general from snail hosts, and none from snails within Canada. While lymnaeid snails have previously been indicated as intermediate hosts for *Haematoloechus *spp. (Gibson et al., 2016), to our knowledge, none have been specifically identified from *S. elodes*. Several other snail families (Physidae and Planorbidae) are also hosts for different species of *Haematoloechus*, indicating they do not specialize by snail family, but could specialize for snail species, which may be regionally determined (Gibson et al., 2016). For both families, records within Canada have all come from the Eastern provinces (Quebec, Ontario, New Brunswick, and Nova Scotia) and from definitive hosts (Psilostomidae: Anatid birds and aquatic mammals; Haematoloechidae: frogs; Gibson et al., 2016). It is impossible at this point to know whether the presence of species from these families is from recent introductions or not, but they are rare in the fact that we only collected a few from each family over the course of 3 years. Considering their host species are quite prevalent across Alberta, it is possible that they have been here and remained undetected, but they could also have expanded their distributions westward into Alberta as well.

The gap between morphological and molecular species identities is growing larger, and the effort to find a solution is not growing at the same rate. Without the link between the two, we are missing important information about life cycle dynamics due to host associations and infection processes that could help inform wildlife managers and possibly influence control efforts for human and veterinary diseases caused by trematodes. One possible solution to this, aside from more molecular data from adult worm samples, is the development of methods to derive quality sequence information from historical, adult trematode specimens. As these vouchers have been our historical standard for species identifications, they are our ultimate source for generating molecular libraries by which to further our understanding of trematode diversity, speciation, and evolution with the added benefit of linking life cycles.

Furthermore, we urge the contribution of sequences that represent a broader diversity of digenean trematodes. One current issue is that novel lineages in molecular phylogenies could either represent cryptic species or they could represent described species for which we have no/limited molecular resources. Therefore, placing emphasis on capturing a broader diversity of trematodes might help bridge knowledge gaps.

## CONFLICT OF INTEREST

None declared.

## AUTHOR CONTRIBUTIONS

MAG designed and performed the research, completed all analyses, and wrote the paper. PCH was involved with conception and design of the research, reviewing the results, and contributed to writing the paper.

## Data Availability

DNA sequences: GenBank accessions MH368808–MH369793, KT831346–KT831348, KT831353, KT831356, KT831357, KT831359, KT831360, KT831363, KT831366, KT831371, KT831372, KT831377, KT831378, KT831382, KY207550, KY207557, KY207558, KY207582, KY207583, and KY207596. Sequence alignments and phylogenetic trees: TreeBASE (read only access until manuscript is approved) http://purl.org/phylo/treebase/phylows/study/TB2:S23234?x-access-code=c870e4e7ccd090a8c9a0ad25ac312dee&format=html. Host and location data: in Table 3.

## APPENDIX A

### A.1

**Table 6:** **Average** ***cox1*** **divergence within and between groups in the Notocotylidae.** The number of base differences per site from between sequences are shown. Standard error estimate(s) are shown above the diagonal. The rate variation among sites was modeled with a gamma distribution (shape parameter = 1). The analysis involved 98 nucleotide sequences. Codon positions included were 1st + 2nd + 3rd + Noncoding. All positions containing gaps and missing data were eliminated. There were a total of 322 positions in the final dataset. Standard error estimates are shown above the diagonal. Average within group divergence is given on the diagonal. Group intraspecific divergence ranges are given as percentages next to species names. Numbers in red are outside the delineation cut‐off

	*E. hortense*	*N*. sp. A	*N*. sp. B	*N*. sp. C	*N*. sp. D	*O. sikae*
*Echinostoma hortense* (out)	**‐**	0.023	0.023	0.023	0.023	0.023
*Notocotylus* sp. A (0.0–4.3%)	0.274	**0.013**	0.012	0.014	0.008	0.020
*Notocotylus* sp. B	0.270	0.059	**‐**	0.016	0.014	0.020
*Notocotylus* sp. C	0.261	0.083	0.102	**‐**	0.014	0.021
*Notocotylus* sp. D (0.0–4.3%)	0.273	0.038	0.084	0.088	**0.013**	0.020
*Ogmocotyle sikae*	0.258	0.188	0.196	0.208	0.198	**‐**

**Table 7:** **Pairwise distance between individual** ***cox1*** **sequences in the Psilostomidae.** The number of base differences per site from between sequences are shown. Standard error estimate(s) are shown above the diagonal. The rate variation among sites was modeled with a gamma distribution (shape parameter = 1). The analysis involved 11 nucleotide sequences. Codon positions included were 1st+2nd+3rd+Noncoding. All positions containing gaps and missing data were eliminated. There were a total of 496 positions in the final dataset

	MGC342	MGC2085	MGC2089	MGC406	KT831366	MGC1319	*E. japonicus*	*P. varium*	*S. globulus*	*S. pseudoglobulus*	*S. pseudoglobulus*
*Psilostomatidae* gen. sp. A*** MGC342		0.004	0.004	0.004	0.004	0.004	0.018	0.015	0.019	0.019	0.019
*Psilostomatidae* gen. sp. A*** MGC2085	0.010		0.000	0.002	0.002	0.002	0.018	0.015	0.019	0.019	0.019
*Psilostomatidae* gen. sp. A*** MGC2089	0.010	0.000		0.002	0.002	0.002	0.018	0.015	0.019	0.019	0.019
*Psilostomatidae* gen. sp. A*** MGC406	0.008	0.002	0.002		0.000	0.000	0.018	0.015	0.019	0.019	0.019
*Psilostomatidae* gen. sp. A *** KT831366	0.008	0.002	0.002	0.000		0.000	0.018	0.015	0.019	0.019	0.019
*Psilostomatidae* gen. sp. A*** MGC1319	0.008	0.002	0.002	0.000	0.000		0.018	0.015	0.019	0.019	0.019
*Echinochasmus japonicus* (out)	0.234	0.234	0.234	0.236	0.236	0.236		0.018	0.018	0.018	0.018
*Pseudopsilostoma varium*	0.143	0.145	0.145	0.147	0.147	0.147	0.230		0.019	0.018	0.018
*Sphaeridiotrema globulus*	0.242	0.244	0.244	0.246	0.246	0.246	0.228	0.258		0.018	0.018
*Sphaeridiotrema pseudoglobulus*	0.244	0.246	0.246	0.244	0.244	0.244	0.232	0.254	0.192		0.000
*Sphaeridiotrema pseudoglobulus*	0.244	0.246	0.246	0.244	0.244	0.244	0.232	0.254	0.192	0.000	

**Table 8:** **Pairwise distance between individual** ***cox1*** **sequences in the Haematoloechidae.** The number of base differences per site from between sequences are shown. Standard error estimate(s) are shown above the diagonal. The rate variation among sites was modeled with a gamma distribution (shape parameter = 1). The analysis involved 7 nucleotide sequences. Codon positions included were 1st+2nd+3rd+Noncoding. All positions containing gaps and missing data were eliminated. There were a total of 454 positions in the final dataset

	KT831372	MGC1782	MGC1787	MGC1792	*H*. sp. 9781	*H*.sp. 9782	*Plagiorchis* sp.
*Haematoloechidae* gen. sp. A***KT831372		0.000	0.000	0.000	0.016	0.018	0.020
*Haematoloechidae* gen. sp. A***MGC1782	0.000		0.000	0.000	0.016	0.018	0.020
*Haematoloechidae* gen. sp. A ***MGC1787	0.000	0.000		0.000	0.016	0.018	0.020
*Haematoloechidae* gen. sp. A***MGC1792	0.000	0.000	0.000		0.016	0.018	0.020
*Haematoloechus* sp. BOLD:ACK9781	0.134	0.134	0.134	0.134		0.019	0.020
*Haematoloechus* sp. BOLD:ACK9782	0.207	0.207	0.207	0.207	0.216		0.019
*Plagiorchis* sp. (out) FJ477214	0.240	0.240	0.240	0.240	0.258	0.218	

**Table 9:** **Average** ***cox1*** **divergence within and between groups of** ***Plagiorchis*** **sp.** The number of base differences per site from averaging over all sequence pairs between groups are shown. Standard error estimate(s) are shown above the diagonal. The average within group divergence is given on the diagonal. The range of pairwise distances within each group are given as percentages in the first column after group names. The rate variation among sites was modeled with a gamma distribution (shape parameter = 1). The analysis involved 55 nucleotide sequences. Codon positions included were 1st+2nd+3rd+Noncoding. All positions containing gaps and missing data were eliminated. There were a total of 435 positions in the final dataset

	*P*. sp. LIN1	*P*. sp. LIN2	*P*. sp. LIN3	*P*. sp. LIN4	*P*. sp. LIN5	*P*. sp. LIN6	*P*. sp. LIN7	*P*. sp. LIN8	*P*. sp. LIN9
*Plagiorchis* sp. LIN1 (0.0–2.3%)	**0.010**	0.017	0.017	0.015	0.016	0.017	0.017	0.017	0.016
*Plagiorchis* sp. LIN2	0.165	**‐**	0.017	0.017	0.016	0.017	0.016	0.018	0.017
*Plagiorchis* sp. LIN3 (0.2–0.7%)	0.148	0.178	**0.005**	0.016	0.017	0.017	0.017	0.017	0.018
*Plagiorchis* sp. LIN4 (0.0–0.7%)	0.123	0.145	0.140	**0.003**	0.015	0.016	0.016	0.016	0.016
*Plagiorchis* sp. LIN5 (0.2–0.5%)	0.148	0.152	0.173	0.121	**0.003**	0.016	0.015	0.017	0.017
*Plagiorchis* sp. LIN6	0.169	0.156	0.162	0.143	0.149	**‐**	0.016	0.016	0.016
*Plagiorchis* sp. LIN7 (0.0–0.7%)	0.157	0.158	0.162	0.149	0.125	0.143	**0.004**	0.017	0.017
*Plagiorchis* sp. LIN8 (0.2–1.8%)	0.166	0.188	0.180	0.140	0.159	0.151	0.154	**0.013**	0.012
*Plagiorchis* sp. LIN9 (0.0–1.1%)	0.151	0.167	0.171	0.142	0.150	0.155	0.155	0.089	**0.005**

**Table 10:** **Pairwise distance between individual** ***nad1*** **sequences in the genus** ***Drepanocephalus*** **.** The number of base differences per site from between sequences are shown. Standard error estimate(s) are shown above the diagonal. The rate variation among sites was modeled with a gamma distribution (shape parameter = 1). The analysis involved 6 nucleotide sequences. Codon positions included were 1st+2nd+3rd+Noncoding. All positions containing gaps and missing data were eliminated. There were a total of 388 positions in the final dataset

	*F. hepatica*	MGC2147	MGC2353	*D. auritus*	*D. auritus*	*Drep*. sp.
*Fasciola hepatica* (out)		0.021	0.021	0.021	0.022	0.021
MGC2147: MH368951	0.227		0.000	0.000	0.010	0.018
MGC2353: MH368952	0.227	0.000		0.000	0.010	0.018
*Drepanocephalus auritus* KP053262	0.227	0.000	0.000		0.010	0.018
*Drepanocephalus auritus* KP053263	0.235	0.044	0.044	0.044		0.018
*Drepanocephalus* sp. KP053264	0.245	0.144	0.144	0.144	0.155	

**Table 11:** **Average** ***nad1*** **divergence within and between genera of** ***Neopetasiger*** **.** The number of base differences per site from averaging over all sequence pairs between groups are shown. Standard error estimate(s) are shown above the diagonal. On the diagonal are the average within group divergence estimates. The range of pairwise distances within groups is given in parentheses next to the group names. The rate variation among sites was modeled with a gamma distribution (shape parameter = 1). The analysis involved 20 nucleotide sequences. Codon positions included were 1st+2nd+3rd+Noncoding. All positions containing gaps and missing data were eliminated. There were a total of 308 positions in the final dataset

	*N. islandic*.	*N. necome*.	*Neop*.sp. 1	*Neop*.sp. 2	*Neop*.sp. 3	*Neop*.sp. 4
*Neopetasiger islandicus*	**‐**	0.025	0.020	0.019	0.021	0.022
*Neopetasiger necomense*	0.286	**‐**	0.026	0.026	0.025	0.024
*Neopetasiger* sp. 1	0.172	0.325	**0.000**	0.020	0.023	0.025
*Neopetasiger* sp. 2	0.143	0.318	0.149	**0.000**	0.022	0.024
*Neopetasiger* sp. 3 (0.3–1.3%)	0.162	0.298	0.231	0.193	**0.900**	0.022
*Neopetasiger* sp. 4 (0.0–0.7%)	0.211	0.276	0.282	0.240	0.214	**0.200**

**Table 12:** **Average** ***nad1*** **divergence within and between genera of** ***Echinoparyphium/Hypoderaeum*** **.** The number of base differences per site from averaging over all sequence pairs between groups are shown. Standard error estimate(s) are shown above the diagonal. Intraspecific divergence values are given on the diagonal. The range of *p* distances is given for each group in parentheses as percentages after species names. Numbers highlighted in red are above the 5% cut‐off, while numbers in green represent average interspecific divergence below 5%. The rate variation among sites was modeled with a gamma distribution (shape parameter = 1). The analysis involved 261 nucleotide sequences. Codon positions included were 1st+2nd+3rd+Noncoding. All positions containing gaps and missing data were eliminated. There were a total of 299 positions in the final dataset

	*E. aconia*.	*Ec. ellisi*	*Ec. poulini*	*Ec. recurv*.	*Ec*. sp. A	*Ec*. sp. A2	*Ec*. sp. B	*Ec*. sp. C	*Ec*. sp. D	*Ec*. sp. E	*Ec*. sp. L1	*Ec*. sp. L1A	*Ec*. sp. L1B	*Ec*. sp. L2	*Ec*. sp. L3	*Ec*. sp. L4	*H. conoid*.	*H*. sp. LIN1	*H*. sp. LIN2
*Echinoparyphium aconiatum*	**0.000**	0.023	0.024	0.025	0.023	0.022	0.024	0.023	0.023	0.022	0.023	0.025	0.024	0.022	0.023	0.023	0.023	0.023	0.024
*Ec. ellisi*	0.204	**0.000**	0.022	0.022	0.021	0.021	0.025	0.024	0.024	0.025	0.019	0.021	0.021	0.020	0.022	0.022	0.024	0.023	0.024
*Ec. poulini*	0.207	0.171	**0.000**	0.023	0.022	0.023	0.027	0.025	0.024	0.025	0.022	0.022	0.021	0.020	0.023	0.022	0.024	0.024	0.025
*Ec. recurvatum* (0.7–1.7%)	0.239	0.196	0.202	**0.011**	0.024	0.023	0.026	0.025	0.025	0.025	0.022	0.023	0.022	0.021	0.023	0.022	0.024	0.023	0.024
*Echinoparyphium* sp. A (0.0–5.0%)	0.193	0.176	0.170	0.221	**0.019**	0.010	0.025	0.024	0.023	0.024	0.021	0.021	0.021	0.020	0.019	0.019	0.022	0.022	0.022
*Echinoparyphium* sp. A2	0.181	0.167	0.181	0.204	**0.044**	**‐**	0.025	0.023	0.023	0.024	0.021	0.022	0.022	0.021	0.020	0.019	0.023	0.023	0.023
*Echinoparyhium* sp. B (0.0–1.3%)	0.215	0.241	0.268	0.271	0.233	0.228	**0.004**	0.020	0.023	0.022	0.025	0.025	0.025	0.026	0.026	0.025	0.025	0.025	0.024
*Echinoparyphium* sp. C (3.7%)	0.194	0.221	0.227	0.242	0.220	0.204	0.161	**0.037**	0.021	0.022	0.024	0.026	0.024	0.024	0.024	0.023	0.024	0.024	0.024
*Echinoparyphium* sp. D	0.217	0.221	0.227	0.231	0.214	0.204	0.218	0.187	**‐**	0.021	0.023	0.023	0.023	0.023	0.023	0.023	0.024	0.024	0.025
*Echinoparyphium* sp. E (0.0–1.3%)	0.179	0.247	0.232	0.258	0.224	0.210	0.193	0.171	0.164	**0.008**	0.023	0.024	0.025	0.023	0.024	0.023	0.024	0.025	0.025
*Echinoparyphium* sp. LIN1 (2.0–3.0%)	0.202	0.144	0.173	0.200	0.178	0.172	0.250	0.241	0.224	0.223	**0.025**	0.014	0.017	0.019	0.021	0.021	0.023	0.022	0.023
*Echinoparyphium* sp. LIN1A (0.0–1.0%)	0.233	0.156	0.188	0.216	0.169	0.183	0.251	0.255	0.230	0.227	0.086	**0.002**	0.018	0.020	0.021	0.021	0.024	0.024	0.024
*Echinoparyphium* sp. LIN1B	0.214	0.171	0.171	0.201	0.184	0.181	0.255	0.239	0.227	0.235	0.111	0.120	**‐**	0.020	0.022	0.020	0.022	0.022	0.022
*Echinoparyphium* sp. LIN2 (0.0–**5.7**%)	0.184	0.145	0.143	0.174	0.164	0.171	0.262	0.222	0.210	0.216	0.154	0.157	0.161	**0.012**	0.022	0.020	0.022	0.022	0.023
*Echinoparyphium* sp. LIN3 (2.7%)	0.199	0.184	0.187	0.212	0.146	0.151	0.255	0.236	0.234	0.222	0.174	0.188	0.194	0.190	**0.027**	0.017	0.023	0.023	0.023
*Echinoparyphium* sp. LIN4 (3.7%)	0.196	0.174	0.157	0.181	0.152	0.145	0.242	0.217	0.212	0.209	0.173	0.176	0.174	0.158	0.105	**0.037**	0.024	0.024	0.024
*Hypoderaeum conoideum*	0.204	0.227	0.211	0.227	0.206	0.214	0.251	0.232	0.244	0.227	0.215	0.230	0.201	0.195	0.226	0.217	**‐**	0.014	0.015
*Hypoderaeum* sp. LIN1 (0.0–1.7%)	0.196	0.218	0.219	0.226	0.199	0.205	0.252	0.239	0.239	0.236	0.204	0.214	0.189	0.191	0.223	0.221	0.069	**0.008**	0.011
*Hypoderaeum* sp. LIN2 (0.3–0.7%)	0.221	0.227	0.227	0.222	0.198	0.204	0.229	0.237	0.241	0.247	0.217	0.223	0.196	0.208	0.229	0.224	0.077	**0.048**	**0.004**

**Table 13:** **Average** ***cox1*** **divergence within and between genera of Echinostomatidae.** The number of base differences per site from averaging over all sequence pairs between groups are shown. Standard error estimate(s) are shown above the diagonal. Intraspecific divergence is given on the diagonal. Numbers in red represent groups that exceed the cut‐off. The range of p‐distances is given for each group in parentheses as percentages after species names. The rate variation among sites was modeled with a gamma distribution (shape parameter = 1). The analysis involved 111 nucleotide sequences. Codon positions included were 1st+2nd+3rd+Noncoding. All positions containing gaps and missing data were eliminated. There were a total of 383 positions in the final dataset

	*D. auritus*	*D. mexican*.	*D. spathans*	*E. trivolvis*	*E*. gen. sp.	*E. revol*.B	*Ec*.gen.sp.2	*Ec*.sp.A2	*Ec*.sp.A	*Ec*.sp.C	*Ec*.sp.E	*Ec*.sp.L1A	*Ec*.sp.L2	*Ec*.sp.L3	*H. conoid*.
*Drepanocephalus auritus* (0.0–2.9%)	**0.006**	0.016	0.009	0.020	0.021	0.021	0.022	0.021	0.019	0.021	0.021	0.020	0.018	0.021	0.021
*Drepanocephalus mexicanus* (2.3%)	0.129	**0.023**	0.016	0.020	0.020	0.020	0.022	0.020	0.019	0.020	0.020	0.020	0.018	0.019	0.020
*Drepanocephalus spathans* (0.0%)	0.036	0.132	**0.000**	0.020	0.020	0.021	0.022	0.021	0.020	0.020	0.020	0.021	0.019	0.021	0.021
*Echinostoma trivolvis*	0.261	0.270	0.256	**‐**	0.014	0.017	0.022	0.019	0.019	0.019	0.018	0.018	0.017	0.018	0.018
*Echinostomatidae* gen. sp.	0.293	0.273	0.279	0.097	**‐**	0.016	0.022	0.019	0.018	0.018	0.017	0.018	0.017	0.018	0.018
*Echinostoma revolutum* Lineage B (0.0–1.8%)	0.265	0.273	0.261	0.150	0.140	**0.010**	0.022	0.019	0.018	0.019	0.018	0.018	0.015	0.018	0.018
*Echinostomatidae g*en. sp. 2 (0.0–0.5%)	0.309	0.294	0.305	0.275	0.272	0.265	**0.003**	0.021	0.021	0.020	0.021	0.022	0.020	0.021	0.022
*Echinoparyphium* sp. A2 (0.0–1.6%)	0.253	0.247	0.242	0.199	0.206	0.203	0.261	**0.006**	0.019	0.017	0.018	0.018	0.017	0.019	0.019
*Ecchinoparyphium* sp. A (0.0–3.7%)	0.240	0.245	0.249	0.188	0.191	0.187	0.260	0.206	**0.018**	0.019	0.019	0.016	0.014	0.016	0.019
*Echinoparyphium* sp. C (0.0–1.3%)	0.251	0.241	0.250	0.209	0.184	0.194	0.258	0.167	0.207	**0.007**	0.018	0.019	0.017	0.019	0.018
*Echinoparyphium* sp. E (0.8%)	0.253	0.255	0.256	0.176	0.158	0.187	0.243	0.185	0.190	0.155	**0.008**	0.018	0.017	0.018	0.019
*Echinoparyphium* sp. LIN1 A (0.0–1.6%)	0.247	0.247	0.251	0.162	0.170	0.179	0.269	0.186	0.142	0.204	0.182	**0.003**	0.014	0.015	0.019
*Echinoparyphium* sp. LIN2 (0.3–22.7%)	0.267	0.260	0.272	0.197	0.197	0.184	0.282	0.227	0.164	0.206	0.211	0.141	**0.114**	0.014	0.017
*Echinoparyphium* sp. LIN3	0.243	0.240	0.240	0.170	0.175	0.181	0.257	0.187	0.132	0.195	0.172	0.127	0.145	**‐**	0.019
*Hypoderaeum conoideum*	0.261	0.272	0.261	0.188	0.196	0.206	0.269	0.201	0.205	0.187	0.181	0.191	0.209	0.185	**‐**

**Table 14:** **Average** ***nad1*** **divergence within and between groups of** ***Echinostoma*** **spp.** The number of base differences per site from averaging over all sequence pairs between groups are shown. Standard error estimate(s) are shown above the diagonal. The average within group divergence is given on the diagonal. The range of pairwise distances within each group are given as percentages in the first column after group names. Numbers in red lie outside the delineation cut‐off. The rate variation among sites was modeled with a gamma distribution (shape parameter = 1). The analysis involved 72 nucleotide sequences. Codon positions included were 1st+2nd+3rd+Noncoding. All positions containing gaps and missing data were eliminated. There were a total of 386 positions in the final dataset

	*E. bolsch*.	*E. caproni*	*E. desert*.	*E. friedi/IG*	*E. miyag*.	*E. nasinc*.	*E. novaze*.	*E. paraen*.	*E. paraul*.	*E. rev*. A	*E. rev. B*	*E. robust*.	*Echino*. sp.	*E. triv*. A	*E. triv*. B
*Echinostoma bolschwense* (0.3%)	**0.003**	0.019	0.018	0.019	0.018	0.018	0.018	0.019	0.018	0.017	0.017	0.017	0.021	0.017	0.017
*Echinostoma caproni* (2.8%)	0.181	**0.028**	0.017	0.019	0.016	0.018	0.016	0.017	0.016	0.017	0.017	0.016	0.021	0.016	0.017
*Echinostoma deserticum*	0.152	0.162	**‐**	0.020	0.017	0.018	0.017	0.019	0.017	0.017	0.017	0.017	0.021	0.017	0.018
*Echinostoma friedi/IG* (0.3–0.5%)	0.191	0.201	0.217	**0.003**	0.019	0.020	0.020	0.020	0.020	0.020	0.020	0.019	0.021	0.020	0.020
*Echinostoma miyagawai* (0.8–5.2%)	0.164	0.136	0.149	0.194	**0.031**	0.017	0.014	0.017	0.015	0.014	0.014	0.012	0.021	0.016	0.016
*Echinostoma nasincovae* (0.3%)	0.161	0.154	0.166	0.201	0.148	**0.003**	0.017	0.016	0.018	0.016	0.016	0.016	0.021	0.015	0.014
*Echinostoma novazealandense* (0.0–0.3%)	0.143	0.120	0.139	0.205	0.092	0.120	**0.002**	0.017	0.015	0.014	0.015	0.014	0.022	0.016	0.016
*Echinostoma paraensei* (0.3%)	0.174	0.141	0.180	0.195	0.151	0.114	0.135	**0.003**	0.017	0.017	0.018	0.016	0.021	0.017	0.016
*Echinostoma paraulum* (0.5%)	0.159	0.141	0.145	0.196	0.106	0.150	0.111	0.141	**0.005**	0.015	0.016	0.014	0.020	0.016	0.017
*Echinostoma revolutum* Lineage A (0.0–5.7%)	0.137	0.139	0.133	0.197	0.107	0.123	0.086	0.148	0.125	**0.010**	0.010	0.014	0.021	0.015	0.016
*Echinostoma revolutum* Lineage B (0.0–1.6%)	0.147	0.144	0.144	0.196	0.102	0.126	0.098	0.154	0.125	0.052	**0.007**	0.014	0.022	0.015	0.015
*Echinostoma robustum* (5.4%)	0.154	0.145	0.155	0.200	0.079	0.131	0.098	0.139	0.096	0.106	0.109	**0.054**	0.021	0.015	0.015
*Echinostoma* sp.	0.237	0.245	0.246	0.262	0.251	0.255	0.261	0.250	0.233	0.250	0.256	0.255	**‐**	0.021	0.021
*Echinostoma trivolvis* Lineage A (0.0–1.3%)	0.148	0.140	0.152	0.190	0.134	0.097	0.123	0.120	0.136	0.115	0.120	0.126	0.237	**0.006**	0.012
*Echinostoma trivolvis* Lineage B (0.3–2.8%)	0.140	0.164	0.180	0.207	0.143	0.098	0.125	0.129	0.148	0.127	0.127	0.125	0.250	0.080	**0.018**

**Table 15:** **Average** ***cox1*** **divergence within and between genera of Diplostomidae-I.** The number of base differences per site from averaging over all sequence pairs between groups are shown below the diagonal. Standard error estimate(s) are shown above the diagonal. On the diagonal are the average within group divergence values. Numbers within parentheses after species names represent the range of percent divergence within groups. Species with three asterisks represent novel species by molecular phylogeny. The analysis involved 196 nucleotide sequences. Codon positions included were 1st+2nd+3rd+Noncoding. All positions containing gaps and missing data were eliminated. There were a total of 334 positions in the final dataset

	*A. ostrow*.	*Alar*. sp. 1	*Alar*. sp. 2	*D. ardeae*	*D. b*. LIN1	*D. b* LIN2	*D. huron*.	*D. indist*.	*D. mergi*	*D. parvi*.	*D. pseudo*.	*Dipl*. sp. 1	*Dipl*. sp. 2	*Dipl*. sp. 4	*Dipl*. sp. 6	*Dipl*. sp. 7	*Dipl*. sp. 8	*Dipl*. sp. 9	*Dipl*. sp. A	*Dipl*. sp. B	*Dipl*. sp. C	*Dipl*. cladeQ	*D*. sp.LIN6	*D. spath*.	*T. aztecae*	*T.clavata*	*T. excav*.	*T. immer*	*T. jenyns*.	*T. masho*.	*T. scheur*.	*T*. sp. 2LIN1	*T*. sp. 2LIN2	*T*. sp. 3	*T*. sp. 4	*T*. sp. 5	*T*. sp. 6	*T*. sp. A	*T*. sp. IBC	*T*. sp. IND
*Austrodiplostomum ostrowskiae* (0.3–0.9%)	**0.006**	0.017	0.016	0.018	0.020	0.020	0.019	0.020	0.020	0.019	0.018	0.020	0.020	0.020	0.019	0.019	0.020	0.021	0.020	0.020	0.020	0.020	0.020	0.019	0.020	0.020	0.018	0.018	0.017	0.020	0.018	0.020	0.018	0.019	0.019	0.019	0.017	0.018	0.016	0.017
*Alaria* sp. 1	0.100	**‐**	0.017	0.018	0.020	0.019	0.019	0.020	0.021	0.020	0.019	0.020	0.020	0.020	0.020	0.021	0.020	0.019	0.019	0.021	0.019	0.021	0.020	0.021	0.018	0.020	0.019	0.017	0.019	0.020	0.019	0.020	0.018	0.018	0.019	0.020	0.019	0.017	0.018	0.018
*Alaria* sp. 2 (0.0%)	0.098	0.099	0.000	0.021	0.021	0.021	0.020	0.021	0.020	0.020	0.020	0.020	0.019	0.021	0.021	0.021	0.021	0.021	0.021	0.020	0.022	0.021	0.021	0.021	0.020	0.020	0.019	0.019	0.018	0.019	0.019	0.020	0.019	0.019	0.019	0.020	0.018	0.019	0.017	0.019
*Diplostomum ardeae*	0.137	0.144	0.162	**‐**	0.019	0.019	0.018	0.017	0.016	0.017	0.017	0.018	0.018	0.017	0.017	0.017	0.016	0.018	0.017	0.018	0.017	0.018	0.019	0.016	0.018	0.018	0.017	0.017	0.019	0.018	0.018	0.020	0.017	0.016	0.018	0.018	0.018	0.016	0.015	0.017
*Diplostomum* baeri LIN1 (0.9%)	0.146	0.169	0.184	0.136	**0.009**	0.018	0.018	0.019	0.019	0.018	0.017	0.019	0.017	0.018	0.016	0.015	0.019	0.019	0.019	0.018	0.019	0.018	0.017	0.018	0.020	0.020	0.020	0.020	0.020	0.020	0.019	0.022	0.020	0.019	0.020	0.020	0.020	0.020	0.019	0.020
*Diplostomum* baeri LIN2 (0.0–0.9%)	0.152	0.150	0.170	0.137	0.125	**0.004**	0.017	0.019	0.018	0.018	0.017	0.018	0.019	0.016	0.017	0.017	0.018	0.019	0.018	0.019	0.016	0.018	0.019	0.018	0.019	0.020	0.018	0.020	0.020	0.019	0.020	0.020	0.018	0.020	0.019	0.019	0.019	0.019	0.020	0.020
*Diplostomum huronense* (0.3–0.6%)	0.147	0.145	0.157	0.128	0.117	0.102	**0.004**	0.016	0.016	0.014	0.017	0.017	0.017	0.017	0.015	0.017	0.019	0.018	0.018	0.017	0.016	0.016	0.016	0.015	0.019	0.019	0.017	0.017	0.019	0.019	0.019	0.018	0.018	0.017	0.017	0.017	0.019	0.018	0.018	0.018
*Diplostomum indistinctum* (0.3–0.9%)	0.171	0.167	0.176	0.132	0.147	0.148	0.096	**0.005**	0.017	0.016	0.016	0.016	0.017	0.017	0.018	0.019	0.019	0.018	0.018	0.019	0.017	0.018	0.019	0.014	0.019	0.020	0.019	0.018	0.019	0.020	0.019	0.021	0.019	0.019	0.019	0.019	0.020	0.018	0.019	0.019
*Diplostomum mergi* (0.0–2.4%)	0.158	0.170	0.157	0.113	0.149	0.134	0.101	0.121	**0.016**	0.013	0.016	0.016	0.017	0.016	0.018	0.018	0.018	0.018	0.016	0.018	0.017	0.016	0.017	0.016	0.019	0.018	0.018	0.018	0.017	0.019	0.017	0.020	0.017	0.018	0.018	0.018	0.017	0.018	0.017	0.018
*Diplostomum parviventosum* (0.6%)	0.151	0.165	0.164	0.107	0.134	0.133	0.086	0.114	0.069	**0.006**	0.017	0.017	0.015	0.016	0.017	0.018	0.018	0.018	0.016	0.017	0.017	0.016	0.016	0.015	0.019	0.019	0.018	0.017	0.017	0.019	0.018	0.018	0.018	0.017	0.017	0.017	0.018	0.017	0.017	0.018
*Diplostomum pseudospathaceum* (0.0–3.3%)	0.138	0.157	0.163	0.128	0.126	0.119	0.109	0.114	0.117	0.124	**0.018**	0.015	0.017	0.015	0.018	0.017	0.017	0.017	0.017	0.018	0.016	0.018	0.018	0.013	0.018	0.020	0.017	0.019	0.019	0.019	0.018	0.020	0.018	0.018	0.018	0.019	0.016	0.017	0.018	0.020
*Diplostomum* sp. 1 (0.0–1.5%)	0.160	0.150	0.151	0.139	0.146	0.138	0.102	0.096	0.112	0.116	0.095	**0.007**	0.017	0.018	0.018	0.018	0.018	0.019	0.017	0.018	0.017	0.018	0.018	0.015	0.018	0.019	0.017	0.019	0.020	0.019	0.019	0.021	0.018	0.019	0.019	0.018	0.019	0.019	0.018	0.020
*Diplostomum* sp. 2	0.146	0.156	0.150	0.123	0.114	0.133	0.109	0.125	0.114	0.092	0.119	0.110	**‐**	0.018	0.016	0.016	0.018	0.018	0.017	0.016	0.018	0.017	0.017	0.017	0.019	0.019	0.019	0.019	0.019	0.019	0.019	0.021	0.019	0.018	0.020	0.019	0.018	0.018	0.018	0.020
*Diplostomum* sp. 4 (0.0–1.5%)	0.156	0.153	0.166	0.120	0.126	0.098	0.097	0.119	0.106	0.107	0.093	0.120	0.122	**0.004**	0.018	0.018	0.017	0.016	0.017	0.018	0.017	0.018	0.017	0.015	0.018	0.019	0.018	0.018	0.019	0.018	0.018	0.019	0.017	0.017	0.018	0.018	0.019	0.017	0.019	0.020
*Diplostomum* sp. 6	0.137	0.156	0.177	0.111	0.102	0.104	0.094	0.131	0.138	0.119	0.131	0.133	0.102	0.131	**‐**	0.015	0.017	0.020	0.019	0.018	0.018	0.019	0.017	0.017	0.020	0.020	0.019	0.019	0.019	0.020	0.020	0.020	0.018	0.018	0.019	0.020	0.019	0.019	0.018	0.019
*Diplostomum* sp. 7	0.135	0.162	0.174	0.117	0.084	0.110	0.115	0.158	0.137	0.140	0.120	0.139	0.099	0.129	0.078	**‐**	0.018	0.019	0.019	0.017	0.018	0.019	0.017	0.017	0.019	0.019	0.019	0.020	0.021	0.020	0.020	0.020	0.019	0.020	0.020	0.020	0.019	0.019	0.019	0.020
*Diplostomum* sp. 8	0.161	0.156	0.171	0.099	0.141	0.119	0.124	0.137	0.123	0.125	0.112	0.121	0.108	0.114	0.111	0.126	**‐**	0.017	0.018	0.018	0.016	0.018	0.015	0.017	0.018	0.019	0.018	0.019	0.019	0.019	0.018	0.020	0.018	0.018	0.018	0.020	0.018	0.019	0.018	0.020
*Diplostomum* sp. 9	0.170	0.147	0.174	0.135	0.138	0.149	0.128	0.138	0.130	0.124	0.124	0.136	0.123	0.109	0.150	0.138	0.114	**‐**	0.019	0.019	0.016	0.018	0.016	0.017	0.019	0.021	0.020	0.019	0.019	0.020	0.019	0.021	0.020	0.019	0.020	0.020	0.019	0.019	0.019	0.020
*Diplostomum* sp. A***	0.149	0.144	0.165	0.123	0.141	0.137	0.126	0.132	0.118	0.114	0.127	0.109	0.117	0.123	0.141	0.138	0.132	0.132	**‐**	0.018	0.017	0.017	0.019	0.017	0.020	0.019	0.019	0.019	0.020	0.019	0.019	0.021	0.018	0.019	0.020	0.019	0.020	0.017	0.019	0.019
*Diplostomum* sp. B***	0.153	0.168	0.159	0.138	0.120	0.135	0.112	0.143	0.125	0.111	0.122	0.125	0.090	0.135	0.129	0.111	0.120	0.135	0.132	**‐**	0.017	0.018	0.017	0.017	0.020	0.018	0.019	0.020	0.020	0.020	0.020	0.021	0.020	0.020	0.020	0.019	0.019	0.019	0.019	0.020
*Diplostomum* sp. C ***(0.0–2.1%)	0.157	0.149	0.179	0.114	0.141	0.103	0.111	0.125	0.120	0.113	0.107	0.112	0.117	0.111	0.125	0.132	0.092	0.101	0.108	0.112	**0.005**	0.017	0.017	0.017	0.019	0.019	0.018	0.019	0.019	0.019	0.020	0.021	0.019	0.019	0.020	0.019	0.019	0.019	0.019	0.019
*Diplostomum* sp. clade Q	0.163	0.180	0.189	0.123	0.130	0.128	0.097	0.129	0.111	0.107	0.123	0.130	0.114	0.117	0.138	0.144	0.129	0.129	0.123	0.132	0.112	**‐**	0.017	0.017	0.019	0.019	0.018	0.018	0.019	0.020	0.019	0.019	0.019	0.018	0.018	0.018	0.019	0.017	0.018	0.019
*Diplostomum* sp. LIN6 (0.3–1.8%)	0.171	0.171	0.180	0.150	0.124	0.136	0.106	0.139	0.123	0.109	0.129	0.121	0.111	0.120	0.120	0.128	0.085	0.108	0.148	0.116	0.107	0.118	**0.012**	0.018	0.018	0.020	0.019	0.018	0.019	0.020	0.019	0.020	0.020	0.018	0.017	0.019	0.018	0.019	0.019	0.020
*Diplostomum spathaceum* (0.3–2.1%)	0.155	0.171	0.189	0.115	0.128	0.139	0.089	0.092	0.112	0.105	0.088	0.095	0.115	0.091	0.122	0.127	0.117	0.111	0.110	0.128	0.120	0.120	0.128	**0.011**	0.018	0.019	0.018	0.018	0.019	0.019	0.018	0.021	0.019	0.018	0.018	0.017	0.019	0.018	0.017	0.018
*Tylodelphys aztecae* (0.6–1.5%)	0.159	0.138	0.163	0.129	0.157	0.137	0.145	0.159	0.160	0.156	0.131	0.141	0.147	0.132	0.152	0.140	0.132	0.152	0.166	0.164	0.142	0.158	0.141	0.143	**0.010**	0.020	0.018	0.017	0.020	0.019	0.017	0.020	0.019	0.017	0.018	0.018	0.018	0.018	0.017	0.020
*Tylodelphys clavata* (0.6–1.2%)	0.156	0.146	0.157	0.131	0.161	0.167	0.145	0.173	0.145	0.163	0.158	0.155	0.149	0.160	0.160	0.144	0.145	0.178	0.149	0.146	0.165	0.156	0.170	0.157	0.154	**0.008**	0.017	0.020	0.019	0.018	0.018	0.018	0.018	0.019	0.019	0.016	0.019	0.019	0.018	0.018
*Tylodelphys excavata*	0.123	0.138	0.135	0.120	0.156	0.132	0.109	0.155	0.136	0.137	0.123	0.114	0.144	0.135	0.141	0.144	0.129	0.159	0.150	0.141	0.135	0.138	0.139	0.132	0.132	0.111	**‐**	0.018	0.018	0.017	0.018	0.018	0.017	0.016	0.018	0.015	0.017	0.018	0.015	0.019
*Tylodelphys immer* (0.3–0.6%)	0.132	0.121	0.151	0.127	0.168	0.143	0.113	0.133	0.138	0.133	0.153	0.143	0.144	0.126	0.151	0.157	0.147	0.151	0.139	0.162	0.143	0.128	0.132	0.136	0.125	0.147	0.126	**0.004**	0.016	0.017	0.016	0.018	0.018	0.014	0.017	0.016	0.018	0.015	0.014	0.016
*Tylodelphys jenynsiae* (0.0–0.9%)	0.112	0.137	0.135	0.135	0.168	0.158	0.142	0.159	0.140	0.138	0.149	0.165	0.158	0.143	0.158	0.167	0.146	0.160	0.156	0.166	0.149	0.141	0.148	0.144	0.146	0.152	0.132	0.104	**0.006**	0.018	0.017	0.018	0.018	0.017	0.015	0.018	0.015	0.018	0.016	0.018
*Tylodelphys mashonensis* (0.0–0.3%)	0.146	0.143	0.138	0.121	0.149	0.152	0.141	0.157	0.149	0.144	0.156	0.148	0.152	0.136	0.149	0.149	0.135	0.146	0.146	0.164	0.144	0.152	0.159	0.158	0.147	0.126	0.116	0.120	0.132	**0.001**	0.017	0.018	0.015	0.017	0.018	0.018	0.017	0.017	0.017	0.018
*Tylodelphys scheuringi* (0.0–1.2%)	0.129	0.135	0.134	0.120	0.158	0.159	0.145	0.158	0.132	0.141	0.136	0.148	0.146	0.138	0.156	0.165	0.131	0.144	0.146	0.167	0.152	0.146	0.155	0.145	0.118	0.140	0.129	0.118	0.116	0.126	**0.005**	0.018	0.018	0.014	0.017	0.017	0.018	0.014	0.015	0.018
*Tylodelphys* sp. 2 LIN1	0.144	0.147	0.156	0.147	0.183	0.165	0.133	0.168	0.161	0.144	0.152	0.170	0.174	0.143	0.165	0.183	0.153	0.183	0.171	0.186	0.163	0.162	0.171	0.162	0.161	0.134	0.114	0.127	0.120	0.124	0.126	**‐**	0.017	0.018	0.018	0.017	0.019	0.018	0.018	0.018
*Tylodelphys* sp. 2 LIN2 (0.9%)	0.132	0.126	0.145	0.112	0.156	0.129	0.128	0.153	0.124	0.133	0.127	0.140	0.153	0.124	0.133	0.147	0.135	0.175	0.136	0.156	0.150	0.144	0.162	0.144	0.147	0.123	0.108	0.124	0.125	0.097	0.130	0.097	**0.009**	0.016	0.018	0.017	0.017	0.015	0.017	0.019
*Tylodelphys* sp. 3 (0.3–0.9%)	0.132	0.123	0.134	0.108	0.150	0.147	0.123	0.139	0.134	0.124	0.132	0.135	0.125	0.115	0.132	0.154	0.119	0.131	0.141	0.154	0.134	0.137	0.129	0.129	0.122	0.139	0.107	0.079	0.118	0.111	0.081	0.124	0.112	**0.006**	0.015	0.016	0.017	0.014	0.013	0.017
*Tylodelphys* sp. 4 (0.0–0.3%)	0.136	0.146	0.143	0.119	0.153	0.144	0.121	0.148	0.139	0.124	0.140	0.160	0.142	0.127	0.134	0.143	0.128	0.159	0.145	0.150	0.157	0.139	0.131	0.138	0.128	0.133	0.131	0.108	0.101	0.130	0.122	0.141	0.127	0.099	**0.004**	0.018	0.014	0.017	0.016	0.017
*Tylodelphys* sp. 5 (0.3%)	0.127	0.133	0.139	0.127	0.147	0.134	0.107	0.147	0.134	0.130	0.143	0.127	0.139	0.122	0.145	0.142	0.145	0.166	0.133	0.133	0.133	0.136	0.146	0.126	0.121	0.103	0.091	0.101	0.126	0.126	0.124	0.115	0.115	0.103	0.120	**0.003**	0.018	0.016	0.017	0.017
*Tylodelphys* sp. 6 (0.6%)	0.104	0.130	0.127	0.118	0.149	0.142	0.145	0.161	0.135	0.137	0.112	0.149	0.127	0.140	0.135	0.136	0.129	0.171	0.144	0.141	0.147	0.145	0.145	0.151	0.132	0.143	0.124	0.128	0.086	0.122	0.122	0.136	0.114	0.111	0.082	0.117	**0.006**	0.017	0.016	0.018
*Tylodelphys* sp. A*** (0.0–0.9%)	0.122	0.105	0.138	0.108	0.146	0.138	0.125	0.131	0.134	0.124	0.128	0.139	0.117	0.118	0.139	0.139	0.133	0.135	0.109	0.149	0.147	0.126	0.146	0.129	0.124	0.143	0.129	0.084	0.111	0.110	0.074	0.125	0.101	0.071	0.108	0.100	0.097	**0.003**	0.016	0.018
*Tylodelphy* sp. IBC–2016 (0.0–0.6%)	0.106	0.121	0.118	0.094	0.143	0.154	0.125	0.141	0.124	0.114	0.125	0.133	0.118	0.133	0.130	0.141	0.125	0.143	0.135	0.136	0.143	0.126	0.152	0.129	0.116	0.122	0.093	0.075	0.104	0.109	0.086	0.123	0.119	0.072	0.102	0.106	0.100	0.092	**0.004**	0.017
*Tylodelphys* sp. IND (0.3%)	0.112	0.126	0.138	0.114	0.159	0.164	0.125	0.152	0.126	0.135	0.151	0.157	0.150	0.143	0.147	0.153	0.141	0.156	0.142	0.151	0.141	0.139	0.160	0.138	0.152	0.116	0.126	0.118	0.121	0.129	0.127	0.124	0.127	0.114	0.114	0.112	0.124	0.128	0.114	**0.003**

**Table 16:** **Average** ***cox1*** **divergence within and between genera of Diplostomidae-II.** The number of base differences per site from averaging over all sequence pairs between groups are shown. Standard error estimate(s) are shown above the diagonal. On the diagonal are the average within group divergence values. Numbers within parentheses after species names represent the range of percent divergence within groups. The rate variation among sites was modeled with a gamma distribution (shape parameter = 1). The analysis involved 102 nucleotide sequences. Codon positions included were 1st+2nd+3rd+Noncoding. All positions containing gaps and missing data were eliminated. There were a total of 307 positions in the final dataset

	*Alaria* sp.1	*Alaria* sp.2	*B. damni*.	*Bolb*. sp.	*B*. sp.BOLD	*D*.gen.sp.O	*D*.gen.sp.X	*H. triloba*	*N. americ*.	*O.scardinii*	*Orni*. sp. 1	*Orni*. sp. 2	*Orni*. sp. 3	*Orni*. sp. 4	*Orni*. sp. 8	*P. brevic*.	*P.cetrarchi*	*P.cuticola*	*Post*. sp. 1	*Post*. sp. 2	*Post*. sp. 4	*Post*. sp. 5	*Post*. sp. 7	*Post*. sp. 8
*Alaria* sp. 1	**‐**	0.017	0.022	0.022	0.022	0.023	0.022	0.019	0.021	0.022	0.023	0.022	0.022	0.022	0.022	0.022	0.022	0.022	0.023	0.022	0.021	0.023	0.021	0.023
*Alaria* sp. 2 (0.3–1.0%)	0.109	**0.007**	0.022	0.022	0.023	0.023	0.023	0.019	0.021	0.024	0.023	0.023	0.023	0.023	0.023	0.023	0.023	0.022	0.023	0.022	0.022	0.024	0.023	0.022
*Bolbophorus damnificus*	0.189	0.189	**‐**	0.020	0.019	0.023	0.022	0.021	0.021	0.022	0.023	0.021	0.020	0.021	0.022	0.022	0.022	0.022	0.024	0.023	0.021	0.024	0.021	0.022
*Bolbophorus* sp. (0.0–3.9%)	0.202	0.210	0.156	**0.012**	0.020	0.022	0.022	0.022	0.022	0.022	0.023	0.021	0.021	0.021	0.022	0.022	0.022	0.022	0.023	0.022	0.020	0.023	0.021	0.023
*Bolbophorus* sp. BOLD	0.176	0.212	0.121	0.153	**‐**	0.023	0.021	0.022	0.022	0.021	0.022	0.020	0.021	0.021	0.021	0.022	0.022	0.021	0.022	0.021	0.019	0.022	0.022	0.024
*Diplostomidae* gen. sp. O*** (0.0–1.0%)	0.213	0.221	0.203	0.193	0.199	**0.003**	0.018	0.022	0.021	0.018	0.019	0.016	0.016	0.018	0.018	0.021	0.022	0.021	0.021	0.021	0.019	0.020	0.021	0.021
*Diplostomidae* gen. sp. X***	0.202	0.213	0.189	0.202	0.179	0.115	**‐**	0.022	0.022	0.018	0.018	0.014	0.015	0.017	0.018	0.020	0.021	0.021	0.021	0.020	0.018	0.020	0.021	0.022
*Hysteromorpha triloba* (4.2%)	0.151	0.159	0.184	0.211	0.195	0.208	0.213	**0.042**	0.020	0.020	0.021	0.021	0.021	0.022	0.021	0.021	0.021	0.021	0.022	0.022	0.021	0.023	0.021	0.023
*Neodiplostomum americanum* (0.3–1.3%)	0.162	0.163	0.179	0.194	0.182	0.199	0.196	0.176	**0.008**	0.021	0.022	0.022	0.021	0.021	0.020	0.023	0.022	0.022	0.023	0.022	0.021	0.023	0.022	0.022
*Ornithodiplostomum scardinii*	0.202	0.227	0.195	0.199	0.189	0.128	0.121	0.199	0.186	**‐**	0.019	0.017	0.017	0.019	0.019	0.021	0.021	0.021	0.022	0.021	0.020	0.021	0.020	0.023
*Ornithodiplostomum* sp. 1 (0.3%)	0.228	0.232	0.202	0.212	0.189	0.138	0.132	0.195	0.222	0.148	**0.003**	0.018	0.018	0.020	0.019	0.020	0.020	0.022	0.021	0.021	0.019	0.020	0.021	0.022
*Ornithodiplostomum* sp. 2 (0.0–3.9%)	0.207	0.229	0.174	0.191	0.161	0.105	0.082	0.203	0.200	0.113	0.128	**0.023**	0.012	0.017	0.016	0.018	0.020	0.020	0.021	0.020	0.017	0.019	0.019	0.021
*Ornithodiplostomum* sp. 3 (2.0–4.2%)	0.219	0.218	0.174	0.203	0.190	0.107	0.090	0.205	0.197	0.118	0.135	0.064	**0.033**	0.016	0.017	0.019	0.019	0.020	0.021	0.021	0.018	0.020	0.020	0.020
*Ornithodiplostomum* sp. 4 (0.3–2.9%)	0.224	0.224	0.185	0.184	0.173	0.117	0.116	0.215	0.198	0.143	0.158	0.114	0.104	**0.020**	0.017	0.019	0.021	0.021	0.021	0.020	0.018	0.022	0.019	0.022
*Ornithodiplostomum* sp. 8 (0.0–3.9%)	0.212	0.227	0.196	0.196	0.186	0.127	0.129	0.201	0.172	0.128	0.148	0.113	0.117	0.125	**0.013**	0.021	0.020	0.020	0.020	0.019	0.017	0.020	0.020	0.021
*Posthodiplostomum brevicaudatum* (0.3–2.0%)	0.227	0.237	0.204	0.213	0.203	0.155	0.153	0.224	0.239	0.172	0.158	0.137	0.146	0.149	0.176	**0.013**	0.020	0.023	0.022	0.022	0.020	0.021	0.021	0.021
*Posthodiplostomum cetrarchi* (0.0%)	0.212	0.227	0.189	0.210	0.182	0.185	0.176	0.217	0.216	0.173	0.145	0.160	0.159	0.174	0.170	0.155	**0.000**	0.022	0.021	0.020	0.019	0.020	0.020	0.020
*Posthodiplostomum cuticola*	0.205	0.205	0.195	0.196	0.182	0.185	0.182	0.182	0.213	0.186	0.191	0.173	0.178	0.185	0.171	0.227	0.179	**‐**	0.021	0.020	0.019	0.023	0.022	0.022
*Posthodiplostomum* sp. 1	0.215	0.214	0.221	0.202	0.186	0.173	0.166	0.208	0.208	0.195	0.184	0.163	0.178	0.174	0.154	0.197	0.169	0.169	**‐**	0.010	0.021	0.021	0.021	0.020
*Posthodiplostomum* sp. 2	0.189	0.185	0.208	0.196	0.176	0.165	0.160	0.192	0.191	0.189	0.174	0.156	0.167	0.166	0.144	0.192	0.160	0.153	0.036	**‐**	0.020	0.023	0.022	0.022
*Posthodiplostomum* sp. 4 (1.0–4.6%)	0.202	0.215	0.181	0.174	0.159	0.145	0.140	0.189	0.198	0.171	0.145	0.129	0.143	0.149	0.142	0.176	0.148	0.161	0.173	0.167	**0.031**	0.020	0.020	0.020
*Posthodiplostomum* sp. 5	0.238	0.255	0.231	0.226	0.199	0.173	0.169	0.226	0.234	0.186	0.145	0.164	0.180	0.197	0.176	0.187	0.160	0.221	0.169	0.192	0.156	**‐**	0.021	0.022
*Posthodiplostomum* sp. 7 (0.3–1.0%)	0.204	0.228	0.181	0.197	0.195	0.182	0.164	0.194	0.203	0.175	0.189	0.162	0.173	0.151	0.167	0.188	0.182	0.201	0.177	0.190	0.179	0.189	**0.007**	0.021
*Posthodiplostomum* sp. 8 (0.3–0.7%)	0.231	0.213	0.192	0.199	0.228	0.189	0.191	0.226	0.208	0.208	0.194	0.182	0.169	0.182	0.170	0.192	0.168	0.199	0.164	0.177	0.159	0.194	0.183	**0.004**

**Table 17:** **Average** ***cox1*** **divergence within and between groups of Strigeidae-I.** The number of base differences per site from averaging over all sequence pairs between groups are shown. Standard error estimate(s) are shown above the diagonal. Average intraspecific divergence values are in bold and lie on the diagonal. The range of intraspecific values are given as percentages next to species names. The rate variation among sites was modeled with a gamma distribution (shape parameter = 1). The analysis involved 152 nucleotide sequences. Codon positions included were 1st+2nd+3rd+Noncoding. All positions containing gaps and missing data were eliminated. There were a total of 345 positions in the final dataset

	*C. cornut*.	*C. flabell*.	*C. marcog*.	*C*. sp. A	*C*. sp. B	*C*. sp. C	*C*. sp. D	*C*. sp. E	*C*. sp. F	*C*. sp. ‘lutzi’	*C. strigeo*.	*Ca. medio*.	*Ca*. sp.	*Ic. pileatus*	*Ic*. sp. 2	*Ic*. sp. 3	*T. sheurin*.
*Cotylurus cornutus* (0.0–2.9%)	**0.011**	0.010	0.013	0.018	0.017	0.012	0.014	0.013	0.010	0.016	0.015	0.020	0.020	0.019	0.019	0.018	0.021
*C. flabelliformis* (0.3%)	0.042	**0.003**	0.014	0.019	0.017	0.013	0.015	0.013	0.011	0.016	0.016	0.020	0.020	0.019	0.019	0.019	0.021
*C. marcogliesei* (0.0–1.2%)	0.070	0.073	**0.005**	0.018	0.016	0.013	0.015	0.013	0.012	0.016	0.015	0.021	0.020	0.020	0.019	0.018	0.021
*Cotylurus* sp. A (0.0–1.4%)	0.132	0.133	0.134	**0.003**	0.019	0.019	0.018	0.019	0.019	0.021	0.019	0.020	0.020	0.020	0.019	0.018	0.020
*Cotylurus* sp. B	0.127	0.123	0.112	0.136	**‐**	0.017	0.018	0.017	0.017	0.019	0.018	0.020	0.020	0.020	0.019	0.019	0.021
*Cotylurus* sp. C (0.0%)	0.064	0.064	0.071	0.147	0.116	**0.000**	0.014	0.014	0.014	0.016	0.015	0.020	0.020	0.020	0.019	0.019	0.021
*Cotylurus* sp. D	0.084	0.084	0.085	0.130	0.136	0.081	**‐**	0.014	0.015	0.016	0.013	0.019	0.020	0.020	0.019	0.019	0.022
*Cotylurus* sp. E (0.0–0.6%)	0.074	0.069	0.068	0.142	0.118	0.077	0.077	**0.002**	0.013	0.015	0.014	0.021	0.021	0.018	0.017	0.019	0.021
*Cotylurus* sp. F (0.0–0.9%)	0.044	0.043	0.057	0.140	0.117	0.074	0.090	0.062	**0.004**	0.017	0.016	0.020	0.021	0.019	0.020	0.019	0.021
*Cotylurus* sp. ‘lutzi’	0.111	0.100	0.114	0.173	0.151	0.107	0.101	0.104	0.122	**‐**	0.016	0.020	0.020	0.019	0.019	0.018	0.021
*C. strigeoides* (0.0–1.7%)	0.096	0.100	0.085	0.145	0.139	0.097	0.069	0.078	0.098	0.114	**0.007**	0.021	0.021	0.019	0.019	0.019	0.021
*Cardiocephaloides medioconiger* (0.0%)	0.167	0.159	0.186	0.174	0.168	0.168	0.154	0.181	0.175	0.165	0.185	**0.000**	0.015	0.019	0.019	0.018	0.020
*Cardiocephaloides* sp.	0.173	0.174	0.168	0.171	0.159	0.174	0.174	0.180	0.181	0.174	0.182	0.090	**‐**	0.020	0.018	0.018	0.021
*Ichthyocotylurus pileatus*	0.151	0.154	0.166	0.161	0.164	0.170	0.158	0.142	0.156	0.156	0.154	0.156	0.179	**0.002**	0.015	0.015	0.020
*Ichthyocotylurus* sp. 2	0.147	0.148	0.143	0.145	0.136	0.142	0.148	0.117	0.160	0.157	0.142	0.151	0.145	0.092	**‐**	0.014	0.020
*Ichthyocotylurus* sp. 3	0.134	0.142	0.132	0.142	0.145	0.142	0.142	0.140	0.140	0.142	0.146	0.142	0.148	0.095	0.078	**‐**	0.018
*Tylodelphys sheuringi (out)*	0.200	0.194	0.190	0.177	0.194	0.200	0.206	0.195	0.204	0.191	0.209	0.191	0.186	0.175	0.171	0.139	**‐**

**Table 18:** **Average** ***cox1*** **divergence within and between groups of Strigeidae-II.** The number of base differences per site from averaging over all sequence pairs between groups are shown. Standard error estimate(s) are shown above the diagonal. Average intraspecific divergence values lie on the diagonal. The range of intraspecific values are given as percentages next to species names. Values in red are above the delineation cut‐off. The rate variation among sites was modeled with a gamma distribution (shape parameter = 1). The analysis involved 309 nucleotide sequences. Codon positions included were 1st+2nd+3rd+Noncoding. All positions containing gaps and missing data were eliminated. There were a total of 377 positions in the final dataset

	*A. jamies*.	*A*. sp. 1	*A*. sp. 1x	*A*. sp. 3	*A*. sp. 4	*A*. sp. A	*A*. sp. B	*A*. sp. C	*Au. b. LIN1*	*Au. mclaugh*.	*Au. niew*.	*Au*. LIN10	*Au*. LIN2	*Au*. LIN3	*Au*. LIN4	*Au*. LIN5	*Au*. LIN6	*Au*. LIN8	*Au*. LIN9A	*Au*. LIN9B
*Apatemon* sp. ‘jamiesoni’ (0.3%)	**0.003**	0.015	0.014	0.015	0.016	0.016	0.015	0.014	0.018	0.018	0.017	0.018	0.019	0.018	0.018	0.019	0.018	0.018	0.017	0.018
*Apatemon* sp. 1 (0.8%)	0.103	**0.008**	0.009	0.014	0.015	0.014	0.013	0.015	0.018	0.017	0.018	0.017	0.019	0.019	0.018	0.017	0.018	0.017	0.017	0.018
*Apatemon* sp. 1x (0.5%)	0.101	0.038	**0.005**	0.014	0.015	0.014	0.014	0.014	0.017	0.017	0.017	0.016	0.019	0.018	0.017	0.018	0.017	0.017	0.017	0.017
*Apatemon* sp. 3 (0.0%)	0.097	0.092	0.085	**0.000**	0.015	0.016	0.015	0.015	0.017	0.018	0.017	0.017	0.019	0.017	0.017	0.018	0.017	0.016	0.016	0.017
*Apatemon* sp. 4 (0.3%)	0.118	0.095	0.090	0.094	**0.003**	0.018	0.016	0.016	0.018	0.018	0.017	0.017	0.019	0.019	0.017	0.019	0.017	0.017	0.018	0.019
*Apatemon* sp. A ***(0.0–1.0%)	0.124	0.094	0.087	0.114	0.131	**0.004**	0.012	0.015	0.019	0.019	0.019	0.018	0.019	0.018	0.018	0.019	0.018	0.018	0.018	0.018
*Apatemon* sp. B***	0.113	0.084	0.085	0.093	0.107	0.058	**‐**	0.015	0.018	0.019	0.018	0.018	0.019	0.017	0.018	0.019	0.018	0.017	0.017	0.018
*Apatemon* sp. C ***(0.0–0.3%)	0.097	0.091	0.092	0.093	0.099	0.101	0.092	**0.001**	0.017	0.018	0.017	0.017	0.018	0.017	0.017	0.018	0.017	0.017	0.016	0.016
*Australapatemon burti* LIN1 (0.0–6.4%)	0.147	0.138	0.136	0.131	0.142	0.158	0.149	0.126	**0.011**	0.014	0.016	0.013	0.012	0.014	0.013	0.015	0.013	0.015	0.015	0.015
*Australapatemon mclaughlini* (0.0–0.5%)	0.150	0.140	0.133	0.142	0.156	0.165	0.162	0.148	0.097	**0.002**	0.017	0.011	0.014	0.015	0.014	0.015	0.012	0.017	0.015	0.015
*Australapatemon niewiadomski* (0.0–2.1%)	0.146	0.154	0.138	0.145	0.145	0.156	0.157	0.146	0.128	0.142	**0.013**	0.017	0.017	0.017	0.017	0.018	0.017	0.017	0.017	0.017
*Australapatemon* sp. LIN10***	0.139	0.134	0.117	0.127	0.139	0.142	0.143	0.129	0.084	0.050	0.130	**‐**	0.015	0.015	0.013	0.014	0.010	0.016	0.015	0.015
*Australapatemon* sp. LIN2	0.158	0.155	0.149	0.159	0.153	0.170	0.159	0.148	0.075	0.085	0.140	0.093	**‐**	0.014	0.014	0.017	0.014	0.015	0.015	0.015
*Australapatemon* sp. LIN3	0.158	0.160	0.154	0.133	0.150	0.156	0.141	0.135	0.087	0.109	0.132	0.098	0.088	**‐**	0.015	0.016	0.014	0.016	0.016	0.015
*Australapatemon* sp. LIN4 (0.3–4.8%)	0.161	0.158	0.149	0.139	0.148	0.154	0.163	0.151	0.093	0.101	0.134	0.086	0.098	0.104	**0.026**	0.015	0.012	0.014	0.015	0.015
*Australapatemon* sp. LIN5	0.174	0.145	0.146	0.143	0.167	0.172	0.167	0.145	0.113	0.099	0.144	0.077	0.138	0.119	0.117	**‐**	0.015	0.017	0.016	0.016
*Australapatemon* sp. LIN6 (0.0–4.2%)	0.144	0.146	0.129	0.135	0.134	0.154	0.151	0.138	0.086	0.067	0.137	0.045	0.094	0.105	0.081	0.097	**0.017**	0.015	0.015	0.014
*Australapatemon* sp. LIN8 (0.0–1.0%)	0.143	0.140	0.136	0.120	0.136	0.147	0.139	0.140	0.100	0.124	0.136	0.107	0.112	0.117	0.105	0.147	0.109	**0.002**	0.014	0.015
*Australapatemon* sp. LIN9A (0.0–3.2%)	0.141	0.136	0.130	0.116	0.146	0.142	0.132	0.125	0.104	0.112	0.136	0.099	0.097	0.117	0.115	0.127	0.107	0.088	**0.009**	0.011
*Australapatemon* sp. LIN9B (0.3–1.1%)	0.145	0.145	0.133	0.123	0.149	0.141	0.141	0.111	0.102	0.111	0.140	0.097	0.100	0.100	0.117	0.117	0.105	0.101	0.061	**0.007**

**Table 19:** **Digenean trematode diversity of Alberta.** A review of the literature is supplemented with species records gathered from this study and previous molecular studies to account for the known trematodes of Alberta, Canada. Host and location records are provided herein

*Family*	*Trematode Species*	*Life Cycle Stage*	*Locations*	*Snail Host Species*	*Definitive /Other Host*	*GenBank Accession Number(s)*	*Reference*
Allocreadiidae	*Crepidostomum farionis*	Adult	Cold Lake (54.30'N, 110W)	Unidentified	Cisco, Whitefish, Coho Salmon		Leong, T.S. and Holmes, J.C., 1981, J. Fish Biol. 18:693‐713
		Adult	Caribou Lake, Eva Lake, Fleming Lake, Margaret Lake, Pitchimi Lake, Semo Lake, Sucker Lake, Wentzel Lake	Unidentified	Various fish species		Baldwin, R.E. and Goater, C.P., 2003, JP, 89(2):215‐225
	*Crepidostomum isotomum*	Adult	Garner Lake, Alberta	Unidentified	Yellow Perch		Zelmer, D.A. and Arai, H.P., 1998, JP, 84(1):24.28
Bolbophoridae	*Bolbophorus* sp.	Cercaria	Canada: Alberta, Buffalo Lake	*Helisoma trivolvis*	Unidentified	KT831373	Gordy, M.A., et al., 2016, Parasitol. Res. 115(10): 3867‐80.
		Cercaria	Canada: Alberta, Buffalo Lake, Isle Lake, Wabamun Lake	*Helisoma trivolvis*	Unidentified	MH368843, MH368847, MH368850, MH368862, MH368871, MH368892, MH368918, MH368919	Present study
Bunoderidae	*Bunodera luciopercae*	Adult	Cold Lake (54.30'N, 110W)	Unidentified	Unidentified		Leong, T.S. and Holmes, J.C., 1981, J. Fish Biol. 18:693‐713
Diplostomidae	*Diplostomidae* gen. sp. O	Cercaria	Canada: Alberta, Buffalo Lake	*Physa gyrina*	Unidentified	KT831363§	Gordy, M.A., et al., 2016, Parasitol. Res. 115(10): 3867‐80.
		Cercaria	Canada: Alberta, Buffalo Lake, Wabamun Lake, Gull Lake, Isle Lake	*Physa gyrina*	Unidentified	MH368825, MH368851, MH368854, MH368855, MH368879, MH368880, MH368881, MH368882, MH368883, MH368884, MH368885, MH368886, MH368887, MH368888, MH368889, MH368890, MH368893, MH368903, MH368904, MH368905, MH368906, MH368915, MH368916, MH368917, MH368934, MH368935, MH368936, MH368937, MH368938, MH368939, MH368940, MH368941, MH368942	Present study
	*Diplostomidae* gen. sp. X	Cercaria	Canada: Alberta, Isle Lake	*Physa gyrina*	Unidentified	MH368907	Present study
	*Diplostomum adamsi*	Metacercaria	Garner Lake, Alberta	Unidentified	Yellow Perch		Zelmer, D.A. and Arai, H.P., 1998, JP, 84(1):24.28
	*Diplostomum baeri bucculentum*	Metacercaria	NW Territories	Unidentified	Least Cisco		Shostak, et al, 1987, Can. J. Zool., 65
	*Diplostomum baeri* LIN2	Cercaria	Canada: Alberta, Wabamun Lake, Isle Lake	*Stagnicola elodes*	Unidentified	MH368863, MH368874, MH368875, MH368928	Present study
	*Diplostomum indistinctum*	Cercaria	Canada: Alberta, Gull Lake	*Stagnicola elodes*	Unidentified	KT831379	Gordy, M.A., et al., 2016, Parasitol. Res. 115(10): 3867‐80.
	*Diplostomum* sp. 1	Cercaria	Canada: Alberta, Wabamun Lake, Isle Lake	*Stagnicola elodes*	Unidentified	MH368857, MH368896, MH368932, MH368943, MH368945	Present study
	*Diplostomum* sp. 2	unknown	Margaret Lake, AB	Unidentified	Trout Perch		Baldwin, R.E. and Goater, C.P., 2003, JP, 89(2):215‐225
	*Diplostomum* sp. 3	Cercaria	Canada: Alberta, Wabamun Lake	*Lymnaea stagnalis*	Unidentified	KT831358	Gordy, M.A., et al., 2016, Parasitol. Res. 115(10): 3867‐80.
		Cercaria	Canada: Alberta, Wabamun Lake	*Lymnaea stagnalis*	Unidentified	MH368837, MH368858	Present study
	*Diplostomum* sp. 4	Cercaria	Canada: Alberta, Isle Lake	*Stagnicola elodes*	Unidentified	KT831354	Gordy, M.A., et al., 2016, Parasitol. Res. 115(10): 3867‐80.
		Cercaria	Canada: Alberta, Wabamun Lake, Isle Lake, Gull Lake, Buffalo Lake, Lac La Nonne	*Stagnicola elodes*	Unidentified	MH368808, MH368809, MH368813, MH368814, MH368815, MH368816, MH368818, MH368819, MH368820, MH368821, MH368822, MH368823, MH368824, MH368826, MH368827, MH368828, MH368829, MH368830, MH368831, MH368832, MH368833, MH368834, MH368835, MH368836, MH368838, MH368839, MH368840, MH368841, MH368844, MH368845, MH368846, MH368848, MH368849, MH368853, MH368856, MH368859, MH368860, MH368861, MH368864, MH368865, MH368866, MH368867, MH368868, MH368869, MH368870, MH368872, MH368873, MH368876, MH368877, MH368891, MH368898, MH368899, MH368900, MH368901, MH368911, MH368913, MH368914, MH368924, MH368925, MH368926, MH368927, MH368929, MH368930, MH368931, MH368944, MH368946, MH368947, MH368948, MH368949, MH368950	Present study
	*Diplostomum* sp. A	Cercaria	Canada: Alberta, Buffalo Lake	*Stagnicola elodes*	Unidentified	MH368817	Present study
	*Diplostomum* sp. B	Cercaria	Canada: Alberta, Isle Lake	*Stagnicola elodes*	Unidentified	MH368933	Present study
	*Diplostomum* sp. C	Cercaria	Canada: Alberta, Gull Lake, Wabamun Lake, Isle Lake	*Stagnicola elodes*	Unidentified	KT831360§, KT831378§, KT831382§	Gordy, M.A., et al., 2016, Parasitol. Res. 115(10): 3867‐80.
		Cercaria	Canada: Alberta, Gull Lake, Wabamun Lake, Isle Lake	*Stagnicola elodes, Helisoma trivolvis (MGC208)*	Unidentified	MH368810, MH368811, MH368812, MH368852, MH368895, MH368902, MH368921, MH368922, MH368923	Present study
	*Diplostomum spathaceum*	Adult	Cooking Lake	Unidentified	Bonaparte's Gulls		Hair, J.D. and Holmes, J.C., 1970, Can. J. Zool. 48:1129‐1131
		Larval	Cold Lake (54.30'N, 110W)	Unidentified	Whitefish, Lake Trout, 9‐spine stickleback		Leong, T.S. and Holmes, J.C., 1981, J. Fish Biol. 18:693‐713
		Larval	Big Fish Lake, Caribou Lake, Margaret Lake, Wentzel Lake	Unidentified	Various fish species		Baldwin, R.E. and Goater, C.P., 2003, JP, 89(2):215‐225
		Adult	Beaverhill Lake (53.30'N, 112.30'W) and Miquelon Lake (53.15'N, 112.55'W)	Unidentified	California Gull, Ring‐billed Gulls		Vermeer, K. 1969, Can. J. Zool. 47:267‐270
	*Neodiplostomum americanum*	Cercaria	Canada: Alberta, Buffalo Lake	*Stagnicola elodes*	Unidentified	KT831357§	Gordy, M.A., et al., 2016, Parasitol. Res. 115(10): 3867‐80.
	*Ornithodiplostomum ptychocheilus*	Cercaria	Lake Wabamun (114.35'W, 53.32'N)	*Physa gyrina*	Unidentified		Sankurathri, C.S. and Holmes, J.C., 1976, Can. J. Zool. 54:1742‐1753
		Cercaria/Metacercaria	Central Alberta unnamed lake (54.22'N, 113.27'W)	*Physa gyrina*	Fathead minnows, chickens(experimental)		Schleppe, J.L. and Goater, C.P., 2004, JP, 90(6):1387‐1390
	*Ornithodiplostomum* sp. 2	Cercaria	Canada: Alberta, Wabamun Lake	*Physa gyrina*	Unidentified	KT831368	Gordy, M.A., et al., 2016, Parasitol. Res. 115(10): 3867‐80.
		Cercaria	Canada: Alberta, Wabamun Lake	*Physa gyrina*	Unidentified	KT831368	Gordy, M.A., et al., 2016, Parasitol. Res. 115(10): 3867‐80.
	*Ornithodiplostomum* sp. 8	Cercaria	Canada: Alberta, Pigeon Lake	*Physa gyrina*	Unidentified	KT831383	Gordy, M.A., et al., 2016, Parasitol. Res. 115(10): 3867‐80.
		Cercaria	Canada: Alberta, Isle Lake	*Physa gyrina*	Unidentified	MH368908, MH368910, MH368920	Present study
	*Posthodiplostomum minimum*	Cercaria/Metacercaria	Central Alberta unnamed lake (54.22'N, 113.27'W)	*Physa gyrina*	Fathead minnows, chickens(experimental)		Schleppe, J.L. and Goater, C.P., 2004, JP, 90(6):1387‐1390
	*Posthodiplostomum* sp. 4	Cercaria	Canada: Alberta, Isle Lake	*Physa gyrina*	Unidentified	MH368909, MH368912	Present study
	*Tylodelphys podicipina*	Adult	9 lakes in Alberta	Unidentified	*Aechmophorus occidentalis, Podiceps grisegena, Podiceps nigricollis*		Stock, T.M. and Holmes, J.C., 1988, JP, 74(2): 214‐227
	*Tylodelphys scheuringi*	Metacercaria	Garner Lake, Alberta	Unidentified	Yellow perch		Zelmer, D.A. and Arai, H.P., 1998, JP, 84(1):24.28
	*Tylodelphys* sp. A	Cercaria	Canada: Alberta, Wabamun Lake	*Helisoma trivolvis*	Unidentified	KT831356§	Gordy, M.A., et al., 2016, Parasitol. Res. 115(10): 3867‐80.
		Cercaria	Canada: Alberta, Wabamun Lake	*Helisoma trivolvis*	Unidentified	MH368842, MH368878, MH368894, MH368897	Present study
Echinostomatidae	*Drepanocephalus spathans*	Cercaria	Canada: Alberta, Isle Lake, Buffalo Lake	*Helisoma trivolvis*	Unidentified	MH368951, MH368952, MH369294	Present study
		Cercaria	Canada: Alberta, Buffalo Lake	*Helisoma trivolvis*	Unidentified	KT831381	Gordy, M.A., et al., 2016, Parasitol. Res. 115(10): 3867‐80.
	*Echinoparyphium recurvatum*	Cercaria/Metacercaria	Lake Wabamun (114.35'W, 53.32'N)	*Physa gyrina*	Unidentified		Sankurathri, C.S. and Holmes, J.C., 1976, Can. J. Zool. 54:1742‐1753
		Redia	Isolated pond near Clyde, AB (54.09'N, 113.39'W)	*Helisoma trivolvis*	Unidentified		Morris and Boag, 1982, Can. J. Zool.
		Adult	13 Lakes in Alberta	Unidentified	Lesser Scaup		Bush, A.O. and Holmes, J.C., 1986, Can. J. Zool. 64:132‐141
		Adult	Alberta	Unidentified	Great horned owls		Ramalingam, S. and Samuel, W.M., 1978, Can. J. Zool. 56:2454‐2456
	*Echinoparyphium recurvatum, flexum*	Adult	Beaverhill Lake (53.30'N, 112.30'W) and Miquelon Lake (53.15'N, 112.55'W)	Unidentified	California Gull, Ring‐billed Gulls		Vermeer, K. 1969, Can. J. Zool. 47:267‐270
	*Echinoparyphium* sp. 1A	Cercaria	Canada: Alberta, Lac La Nonne, Wabamun Lake, Isle Lake	*Physa gyrina, Stagnicola elodes (MGC1954, MGC2104), Helisoma trivolvis (MGC2090)*	Unidentified	MH368998, MH368999, MH369001, MH369002, MH369003, MH369004, MH369005, MH369006, MH369007, MH369008, MH369009, MH369010, MH369012, MH369013, MH369014, MH369015, MH369016, MH369017, MH369018, MH369019, MH369022, MH369023, MH369024, MH369025, MH369026, MH369028, MH369031, MH369032, MH369033, MH369034, MH369038, MH369042, MH369044, MH369045, MH369046, MH369047, MH369048, MH369049, MH369052, MH369053, MH369054, MH369055, MH369056, MH369059, MH369060, MH369062, MH369063, MH369065, MH369066, MH369068, MH369070, MH369075, MH369076, MH369087, MH369089, MH369090, MH369091, MH369093, MH369094, MH369095, MH369096, MH369097, MH369098, MH369099, MH369100, MH369101, MH369102, MH369121, MH369122, MH369123, MH369125, MH369131, MH369132, MH369133, MH369136, MH369147, MH369155, MH369156, MH369162, MH369163, MH369164, MH369165, MH369166, MH369167, MH369168, MH369178, MH369188, MH369191	Present study
		Cercaria	Canada: Alberta, Lac La Nonne	*Physa gyrina*	Unidentified	KT831361§	Gordy, M.A., et al., 2016, Parasitol. Res. 115(10): 3867‐80.
	*Echinoparyphium* sp. 1B	Cercaria	Canada: Alberta, Isle Lake	*Physa gyrina*	Unidentified	MH369181	Present study
	*Echinoparyphium* sp. A	Cercaria	Canada: Alberta, Buffalo Lake, Wabamun Lake, Isle Lake, Lac La Nonne, Gull Lake, Pigeon Lake	*Physa gyrina, Stagnicola elodes (MGC1932)*	Unidentified	MH369011, MH369035, MH369043, MH369051, MH369058, MH369061, MH369064, MH369069, MH369081, MH369082, MH369083, MH369084, MH369085, MH369113, MH369120, MH369128, MH369161, MH369169, MH369170, MH369171, MH369172, MH369173, MH369174, MH369175, MH369176, MH369177, MH369179, MH369180, MH369182, MH369183, MH369184, MH369185, MH369187	Present study
	*Echinoparyphium* sp. A2	Cercaria	Canada: Alberta, Gull Lake	*Physa gyrina*	Unidentified	MH369190, MH369127	Present study
		Cercaria	Canada: Alberta, Lac La Nonne	*Stagnicola elodes*	Unidentified	KT831367§	Gordy, M.A., et al., 2016, Parasitol. Res. 115(10): 3867‐80.
	*Echinoparyphium* sp. B	Cercaria	Canada: Alberta, Lac La Nonne	*Stagnicola elodes*	Unidentified	MH368969, MH368970, MH368971, MH368987, MH368988, MH369041, MH369074, MH369086, MH369092	Present study
	*Echinoparyphium* sp. C	Cercaria	Canada: Alberta, Gull Lake, Lac La Nonne	*Stagnicola elodes*	Unidentified	MH369088, MH369152	Present study
	*Echinoparyphium* sp. D	Cercaria	Canada: Alberta, Buffalo Lake	*Stagnicola elodes*	Unidentified	MH369189	Present study
	*Echinoparyphium* sp. E	Cercaria	Canada: Alberta, Gull Lake	*Stagnicola elodes, Lymnaea stagnalis (MGC1878)*	Unidentified	MH369109, MH369129, MH369134, MH369135, MH369159	Present study
	*Echinoparyphium* sp. Lineage 2	Cercaria	Canada: Alberta, Gull Lake, Isle Lake, Buffalo Lake, Wabamun Lake, Lac La Nonne	*Stagnicola elodea, Lymnaea stagnalis (MGC16A/B, MGC369), Helisoma trivolvis (MGC219)*	Unidentified	MH368953, MH368954, MH368955, MH368956, MH368957, MH368959, MH368960, MH368961, MH368962, MH368963, MH368964, MH368965, MH368966, MH368967, MH368968, MH368972, MH368973, MH368974, MH368975, MH368976, MH368977, MH368978, MH368979, MH368980, MH368981, MH368982, MH368983, MH368984, MH368985, MH368986, MH368989, MH368990, MH368991, MH368992, MH368993, MH368994, MH368995, MH368996, MH368997, MH369000, MH369021, MH369027, MH369029, MH369036, MH369037, MH369039, MH369050, MH369057, MH369067, MH369071, MH369072, MH369073, MH369077, MH369078, MH369079, MH369103, MH369104, MH369105, MH369106, MH369107, MH369111, MH369112, MH369114, MH369115, MH369116, MH369117, MH369118, MH369119, MH369124, MH369126, MH369137, MH369138, MH369139, MH369140, MH369141, MH369142, MH369143, MH369144, MH369146, MH369148, MH369149, MH369150, MH369151, MH369153, MH369154, MH369160, MH369186	Present study
		Cercaria	Canada: Alberta, Lac La Nonne	*Stagnicola elodes*	Unidentified	KT831350§	Gordy, M.A., et al., 2016, Parasitol. Res. 115(10): 3867‐80.
	*Echinoparyphium* sp. Lineage 4	Cercaria	Canada: Alberta, Wabamun Lake, Buffalo Lake	*Helisoma trivolvis*	Unidentified	MH369130, MH369158	Present study
	*Echinostoma revolutum*	Adult	Beaverhill Lake (53.30'N, 112.30'W) and Miquelon Lake (53.15'N, 112.55'W)	Unidentified	California Gull, Ring‐billed Gulls		Vermeer, K. 1969, Can. J. Zool. 47:267‐270
		Adult	Alberta	Unidentified	Great horned owls		Ramalingam, S. and Samuel, W.M., 1978, Can. J. Zool. 56:2454‐2456
	*Echinostoma revolutum* Lineage B	Cercaria	Canada: Alberta, Buffalo Lake, Gull Lake, Wabamun Lake, Isle Lake, Lac La Nonne	*Stagnicola elodes*	Unidentified	MH369227, MH369229, MH369230, MH369231, MH369235, MH369242, MH369248, MH369268, MH369279, MH369281, MH369284, MH369286, MH369287, MH369292	Present study
		Cercaria	Canada: Alberta, Buffalo Lake, Gull Lake, Wabamun Lake, Isle Lake, Lac La Nonne	*Stagnicola elodes*	Unidentified	MH369192, MH369193, MH369194, MH369195, MH369196, MH369197, MH369200, MH369201, MH369202, MH369204, MH369206, MH369207, MH369208, MH369209, MH369210, MH369211, MH369213, MH369214, MH369215, MH369216, MH369217, MH369218, MH369219, MH369220, MH369221, MH369222	Present study
	*Echinostoma trivolvis* Lineage A	Cercaria	Canada: Alberta, Isle Lake, Wabamun Lake, Lac La Nonne	*Helisoma trivolvis*	Unidentified	MH369271	Present study
	*Echinostomatidae* gen. sp.	Cercaria	Canada: Alberta, Buffalo Lake	*Stagnicola elodes*	Unidentified	MH369269, MH369295, MH369297	Present study
	*Hypoderaeum* sp. Lineage 1	Cercaria	Canada: Alberta, Gull Lake, Lac La Nonne, Wabamun Lake, Isle Lake	*Stagnicola elodes*	Unidentified	MH368958, MH369040, MH369108, MH369110, MH369145, MH369157	Present study
	*Hypoderaeum* sp. Lineage 2	Cercaria	Canada: Alberta, Isle Lake, Lac La Nonne	*Stagnicola elodes*	Unidentified	MH369020, MH369030, MH369080	Present study
	*Neopetasiger islandicus*	Cercaria	Canada: Alberta, Wabamun Lake	*Planorbula armigera*	Unidentified	KT831342	Gordy, M.A., et al., 2016, Parasitol. Res. 115(10): 3867‐80.
	*Petasiger nitidius*	Cercaria	Amisk Lake (54.35'N, 112.37'W) Baptiste Lake (54.45'N, 113.33'W)	*Heliosoma trivolvis*	Unidentified		Shostak, A.W., 1992, Can. J. Zool. 71:431‐434
		Adult	9 lakes in Alberta	Unidentified	*Aechmophorus occidentalis, Podiceps grisegena, Podiceps nigricollis, Podiceps auritus*		Stock, T.M. and Holmes, J.C., 1988, JP, 74(2): 214‐227
	*Neopetasiger* sp. 4	Cercaria	Canada: Alberta, Wabamun Lake	*Helisoma trivolvis*	Unidentified	KT831343, KT831345	Gordy, M.A., et al., 2016, Parasitol. Res. 115(10): 3867‐80.
		Cercaria	Canada: Alberta, Wabamun Lake, Isle Lake, Buffalo Lake	*Helisoma trivolvis*	Unidentified	MH369311, MH369312, MH369313, MH369314, MH369315, MH369316, MH369317, MH369318	Present study
Fasciolidae	*Fasciola hepatica*	Adult	Rimbey, Alberta	Unidentified	Cattle (Holstein steers)		Giebelhaus, I.T. 1998, Can. Vet. J. 39:433
	*Fascioloides magna*	Adult	Banff National Park (51.12'N, 115.35'W)	Unidentified	*Cervus elaphus canadensis*		Kralova‐Hromadova, et al., 2010, IJP, 41:373‐383
		Adult	Cypress Hills, Elk Island and other parts of Alberta	Unidentified	Moose		Samuel, W.M., 1976, Can J Zool, 54(3)
Gorgoderidae	*Gorgoderina simplex*	Adult	Eastern Alberta (50.35’‐56.44'N, 110.40’‐114.05'W)	Unidentified	*Bufo hemiophrys* (Canadian Toad)		Bursey, C.R. and Goldberg, S.R. 1998, JP, 84(3):617‐618
	*Phyllodistomum coregoni*	Adult	Cold Lake (54.30'N, 110W)	Unidentified	Whitefish		Leong, T.S. and Holmes, J.C., 1981, J. Fish Biol. 18:693‐713
Haematoloechidae	*Haematoloechidae* gen. sp. A	Cercaria	Canada: Alberta, Buffalo Lake	*Stagnicola elodes*	Unidentified	KT831372§	Gordy, M.A., et al., 2016, Parasitol. Res. 115(10): 3867‐80.
		Cercaria	Canada: Alberta, Buffalo Lake	*Stagnicola elodes*	Unidentified	MH369319, MH369320, MH369321	Present study
Lissorchiidae	*Lissorchis attenuatum*	Adult	Cold Lake (54.30'N, 110W)	Unidentified	9‐spine stickleback		Leong, T.S. and Holmes, J.C., 1981, J. Fish Biol. 18:693‐713
Notocotylidae	*Notocotylus* sp. A	Cercaria	Canada: Alberta, Gull Lake	*Stagnicola elodes*	Unidentified	KT831348§, KT831364	Gordy, M.A., et al., 2016, Parasitol. Res. 115(10): 3867‐80.
		Cercaria	Canada: Alberta, Wabamun Lake, Isle Lake, Gull Lake, Buffalo Lake, Lac La Nonne	*Physa gyrina, Stagnicola elodes*	Unidentified	MH369326, MH369333, MH369334, MH369335, MH369345, MH369346, MH369349, MH369350, MH369352, MH369411, MH369353, MH369354, MH369355, MH369362, MH369363, MH369364, MH369365, MH369366, MH369367, MH369372, MH369373, MH369378, MH369381, MH369382, MH369383, MH369415, MH369384, MH369387, MH369390, MH369391, MH369417, MH369393, MH369395, MH369396, MH369397, MH369398, MH369400, MH369401, MH369402	Present study
	*Notocotylus* sp. B	Cercaria	Canada: Alberta, Wabamun Lake	*Physa gyrina*	Unidentified	MH369416	Present study
	*Notocotylus* sp. C	Cercaria	Canada: Alberta, Lac La Nonne	*Helisoma trivolvis*	Unidentified	MH369356	Present study
	*Notocotylus* sp. D	Cercaria	Canada: Alberta, Gull Lake	*Stagnicola elodes*	Unidentified	KT831348§	Gordy, M.A., et al., 2016, Parasitol. Res.
		Cercaria	Canada: Alberta, Isle Lake, Gull Lake, Buffalo Lake, Lac La Nonne	*Stagnicola elodes, Physa gyrina*	Unidentified	MH369386, MH369389, MH369392, MH369399, MH369323, MH369405, MH369324, MH369406, MH369325, MH369327, MH369407, MH369408, MH369328, MH369409, MH369329, MH369330, MH369331, MH369332, MH369336, MH369337, MH369338, MH369339, MH369340, MH369341, MH369342, MH369343, MH369344, MH369410, MH369347, MH369348, MH369351, MH369357, MH369358, MH369359, MH369360, MH369361, MH369368, MH369412, MH369369, MH369370, MH369371, MH369374, MH369375, MH369376, MH369413, MH369414, MH369379, MH369380, MH369385, MH369388, MH369394, MH369403, MH369404	Present study
	*Notocotylus attenuatus*	Adult	Breeding resident in Alberta, but found in SW Texas	Unidentified	Green‐winged Teal		Canaris, A.G., Mena, A.C. and Bristol, J.R., 1981, J. Wildlife Dis. 17(1)
		Adult	Alberta	Unidentified	Great horned owls		Ramalingam, S. and Samuel, W.M., 1978, Can. J. Zool. 56:2454‐2456
	*Notocotylus urbanensis*	Cercaria/Metacercaria	Lake Wabamun (114.35'W, 53.32'N)	*Physa gyrina*	Unidentified		Sankurathri, C.S. and Holmes, J.C., 1976, Can. J. Zool. 54:1742‐1753
Paramphistomatidae	*Zygocotyle lunata*	Cercaria/Metacercaria	Lyle Lake (55.12'N, 112.29W) Beaver impoundment near Fort McMurray (56.31'N, 111.19'W)	*Heliosoma trivolvis*	CD1 Mice (experimental)		Shostak, A.W., Dharampaul, S., and Belosevic, M., 1993, J. Parasitol. 79(6):922‐929
Plagiorchiidae	*Plagiorchis elegans*	Adult	Beaverhill Lake (53.30'N, 112.30'W) and Miquelon Lake (53.15'N, 112.55'W)	Unidentified	California Gull, Ring‐billed Gulls		Vermeer, K. 1969, Can. J. Zool. 47:267‐270
	*Plagiorchis* sp.	Adult	Alberta	Unidentified	Great horned owls		Ramalingam, S. and Samuel, W.M., 1978, Can. J. Zool. 56:2454‐2456
	*Plagiorchis* sp. Lineage 1	Cercaria	Canada: Alberta, Gull Lake, Lac La Nonne, Buffalo Lake	*Stagnicola elodes*	Unidentified	MH369420, MH369421, MH369422, MH369433, MH369434, MH369435, MH369441, MH369460, MH369461, MH369463, MH369464	Present study
	*Plagiorchis* sp. Lineage 2	Cercaria	Canada: Alberta, Buffalo Lake	*Stagnicola elodes*	Unidentified	MH369467	Present study
	*Plagiorchis* sp. Lineage 3	Cercaria	Canada: Alberta, Buffalo Lake	*Stagnicola elodes*	Unidentified	MH369442, MH369454, MH369466	Present study
	*Plagiorchis* sp. Lineage 4	Cercaria	Canada: Alberta, Gull Lake, Lac La Nonne, Buffalo Lake, Wabamun Lake, Isle Lake	*Stagnicola elodes*	Unidentified	MH369418, MH369423, MH369425, MH369428, MH369429, MH369431, MH369432, MH369436, MH369437, MH369440, MH369447, MH369452, MH369453, MH369456, MH369462, MH369471	Present study
	*Plagiorchis* sp. Lineage 5	Cercaria	Canada: Alberta, Gull Lake, Lac La Nonne	*Stagnicola elodes*	Unidentified	MH369419, MH369426, MH369427	Present study
	*Plagiorchis* sp. Lineage 6	Cercaria	Canada: Alberta, Buffalo Lake	*Helisoma trivolvis*	Unidentified	MH369470	Present study
	*Plagiorchis* sp. Lineage 7	Cercaria	Canada: Alberta, Buffalo Lake, Gull Lake	*Lymnaea stagnalis*	Unidentified	MH369438, MH369448, MH369455, MH369458, MH369468, MH369469	Present study
	*Plagiorchis* sp. Lineage 8	Cercaria	Canada: Alberta, Buffalo Lake, Gull Lake	*Stagnicola elodes*	Unidentified	MH369449, MH369450, MH369451, MH369459, MH369465	Present study
	*Plagiorchis* sp. Lineage 9	Cercaria	Canada: Alberta, Lac La Nonne, Buffalo Lake	*Stagnicola elodes*	Unidentified	MH369424, MH369430, MH369439, MH369443, MH369444, MH369445, MH369446	Present study
Psilostomidae	*Psilostomidae* gen. sp. A	Cercaria	Canada: Alberta, Wabamun Lake	*Helisoma trivolvis*	Unidentified	MH369477§	Gordy, M.A., et al., 2016, Parasitol. Res. 115(10): 3867‐80.
		Cercaria	Canada: Alberta, Wabamun Lake, Isle Lake	*Helisoma trivolvis*	Unidentified	MH369473, MH369472, MH369476, MH369474, MH369475	Present study
Renicolidae	*Renicola* sp.	Adult	Beaverhill Lake (53.30'N, 112.30'W) and Miquelon Lake (53.15'N, 112.55'W)	Unidentified	California Gull, Ring‐billed Gulls		Vermeer, K. 1969, Can. J. Zool. 47:267‐270
Schistosomatidae	*Austrobilharzia* sp.	Adult	Beaverhill Lake (53.30'N, 112.30'W) and Miquelon Lake (53.15'N, 112.55'W)	Unidentified	Ring‐billed Gulls		Vermeer, K. 1969, Can. J. Zool. 47:267‐270
	*Avian Schistosomatid* sp. A	Cercaria	Canada: Alberta, Buffalo Lake, Isle Lake	*Physella gyrina*	Unidentified	MH168789, MH168790, MH168795, MH168796	Gordy, M.A., et al., 2018, Env. Health. 17(1):73
	*Avian Schistosomatid* sp. B	Cercaria	Canada: Alberta, Lac La Nonne	*Physella gyrina*	Unidentified	MH168785	Gordy, M.A., et al., 2018, Env. Health. 17(1):73
	*Avian Schistosomatid* sp. C	Cercaria	Canada: Alberta, Wabamun Lake	*Helisoma trivolvis*	Unidentified	MH168793	Gordy, M.A., et al., 2018, Env. Health. 17(1):73
	*Schistosomatium douthitti*	Cercaria	Canada: Alberta, Gull Lake	*Stagnicola elodes*	Unidentified	KT831376	Gordy, M.A., et al., 2016, Parasitol. Res. 115(10): 3867‐80.
		Cercaria	Canada: Alberta, Buffalo Lake, Gull Lake, Wabamun Lake	*Stagnicola elodes, Lymnaea stagnalis*	Unidentified	MH168791, MH168794	Present study
	*Trichobilharzia cameroni*	Cercaria	Lake Wabamun (114.35'W, 53.32'N)	*Physa gyrina*	Unidentified		Sankurathri, C.S. and Holmes, J.C., 1976, Can. J. Zool. 54:1742‐1753
	*Trichobilharzia physellae*		Canada: Alberta, Lac La Nonne	*Physella gyrina*	Unidentified	MH168784	Gordy, M.A., et al., 2018, Env. Health. 17(1):73
		Cercaria	Lake Wabamun (114.35'W, 53.32'N)	*Physa gyrina*	Unidentified		Sankurathri, C.S. and Holmes, J.C., 1976, Can. J. Zool. 54:1742‐1753
	*Trichobilharzia stagnicolae*	Cercaria	Canada: Alberta, Isle Lake	*Stagnicola elodes*	Unidentified	MH168781, MH168782, MH168786, MH168787, MH168788	Gordy, M.A., et al., 2018, Env. Health. 17(1):73
		Cercaria	Canada: Alberta, Isle Lake	*Stagnicola elodes*	Unidentified	KT831352	Gordy, M.A., et al., 2016, Parasitol. Res. 115(10): 3867‐80.
	*Trichobilharzia szidati*	Cercaria	Canada: Alberta, Gull Lake	*Lymnaea stagnalis*	Unidentified	MH168783	Gordy, M.A., et al., 2018, Env. Health. 17(1):73
		Cercaria	Canada: Alberta, Buffalo Lake	*Lymnaea stagnalis*	Unidentified	KT831375	Gordy, M.A., et al., 2016, Parasitol. Res. 115(10): 3867‐80.
Strigeidae	*Apatemon gracilis*	Cercaria	Lake Wabamun (114.35'W, 53.32'N)	*Physa gyrina*	Unidentified		Sankurathri, C.S. and Holmes, J.C., 1976, Can. J. Zool. 54:1742‐1753
		Adult	13 Lakes in Alberta	Unidentified	Lesser Scaup		Bush, A.O. and Holmes, J.C., 1986, Can. J. Zool. 64:132‐141
		Larval	Cold Lake (54.30'N, 110W)	Unidentified	9‐spine stickleback		Leong, T.S. and Holmes, J.C., 1981, J. Fish Biol. 18:693‐713
		Adult	9 lakes in Alberta	Unidentified	*Aechmophorus occidentalis, Podiceps grisegena, Podiceps nigricollis, Podiceps auritus*		Stock, T.M. and Holmes, J.C., 1988, JP, 74(2): 214‐227
		Adult	Alberta	Unidentified	*Chen hyperborea* and *Mareca americana*		Palmieri, J.R., 1973, JP, 59(6):1063
	*Apatemon* sp. A	Cercaria	Canada: Alberta, Isle Lake	*Stagnicola elodes*	Unidentified	MH369603, MH369604, MH369605, MH369606, MH369607, MH369608, MH369609, MH369610, MH369611, MH369612, MH369613, MH369614, MH369615, MH369616, MH369617	Present study
	*Apatemon* sp. B	Cercaria	Canada: Alberta, Isle Lake	*Stagnicola elodes*	Unidentified	MH369618	Present study
	*Apatemon* sp. C	Cercaria	Canada: Alberta, Isle Lake	*Stagnicola elodes*	Unidentified	MH369619, MH369620, MH369621, MH369622	Present study
	*Apharyngostrigea pipientis*	Adult	Eastern Alberta	Unidentified	Western Chorus Frog		Goldberg, S.R., Bursey, C.R., and Wong, C., 2002, Northwest Science, 76(1)
	*Australapatemon burti* LIN1	Cercaria	Canada: Alberta, Isle Lake	*Stagnicola elodes*	Unidentified	KT831346, KT831351	Gordy, M.A., et al., 2016, Parasitol. Res. 115(10): 3867‐80.
		Cercaria	Canada: Alberta, Isle Lake, Wabamun Lake, Lac La Nonne, Gull Lake, Buffalo Lake	*Stagnicola elodes, Physa gyrina, Helisoma trivolvis, Helisoma campanulatum, Planorbis sp., Lymnaea stagnalis*	Unidentified	KY207548, KY207549, KY207551, KY207552, KY207553, KY207554, KY207555, KY207556, KY207559, KY207560, KY207561, KY207562, KY207563, KY207564, KY207565, KY207566, KY207567, KY207568, KY207570, KY207571, KY207572, KY207573, KY207574, KY207575, KY207576, KY207578, KY207579, KY207580, KY207581, KY207584, KY207585, KY207586, KY207588, KY207589, KY207590, KY207591, KY207592, KY207593, KY207594, KY207595, KY207598, KY207599, KY207600, KY207601, KY207602, KY207603, KY207604, KY207605, KY207606, KY207607, KY207608, KY207609, KY207610, KY207611, KY207612, KY207614, KY207617, KY207618, KY207619, KY207620, KY207621, KY207623, KY207624, KY587399, KY587398, KY587394, KY587401, HM385485, KY587400, HM385486	Gordy, M.A., et al., 2017, Parasitol. Res. 116(8): 2181‐98.
		Cercaria	Canada: Alberta, Isle Lake, Wabamun Lake, Lac La Nonne, Gull Lake, Buffalo Lake	*Stagnicola elodes, Physa gyrina, Helisoma trivolvis*	Unidentified	MH369623, MH369624, MH369625, MH369626, MH369627, MH369628, MH369629, MH369630, MH369631, MH369632, MH369633, MH369634, MH369635, MH369636, MH369637, MH369638, MH369639, MH369640, MH369641, MH369642, MH369643, MH369644, MH369645, MH369646, MH369647, MH369648, MH369649, MH369650, MH369651, MH369652, MH369653, MH369654, MH369655, MH369656, MH369657, MH369658, MH369659, MH369660, MH369661, MH369662, MH369663, MH369664, MH369665, MH369666, MH369667, MH369668, MH369669, MH369670, MH369671, MH369672, MH369673, MH369674, MH369675, MH369676, MH369677, MH369678, MH369679, MH369680, MH369681, MH369682, MH369683, MH369684, MH369686, MH369687, MH369688, MH369689, MH369690, MH369691, MH369692, MH369693, MH369694, MH369695, MH369696, MH369697, MH369698, MH369699, MH369700, MH369701, MH369702, MH369703, MH369704, MH369705, MH369706, MH369707, MH369708, MH369709, MH369710, MH369711, MH369712, MH369713, MH369714, MH369715, MH369716, MH369717, MH369718, MH369719, MH369720, MH369721, MH369722, MH369723, MH369724, MH369725, MH369726, MH369727, MH369728, MH369729, MH369730, MH369731, MH369732, MH369733, MH369734, MH369735, MH369736, MH369737, MH369738, MH369739, MH369740, MH369741, MH369742, MH369743, MH369744, MH369745, MH369746, MH369747, MH369748, MH369749, MH369750, MH369751, MH369752, MH369753, MH369754, MH369755, MH369756, MH369757, MH369758, MH369759, MH369760, MH369761, MH369762, MH369763, MH369685	Present study
	*Australapatemon mclaughlini*	Cercaria, Adult	Canada: Alberta, Buffalo Lake; Ontario	*Physa gyrina*	*Anas acuta*	KY207615, KY207627, KY207628	Gordy, M.A., et al., 2017, Parasitol. Res. 116(8): 2181‐98.
		Cercaria	Canada: Alberta, Buffalo Lake	*Physa gyrina*		MH369764	Present study
	*Australapatemon* sp. LIN2	Adult	Canada: Ontario	Unidentified	*Bucephala albeola*	HM385535	Gordy, M.A., et al., 2017, Parasitol. Res. 116(8): 2181‐98.
	*Australapatemon* sp. LIN3	Cercaria	Canada: Alberta, Gull Lake	*Stagnicola elodes*	Unidentified	KY207577	Gordy, M.A., et al., 2017, Parasitol. Res. 116(8): 2181‐98.
	*Australapatemon* sp. LIN4	Cercaria, Adult	Canada: Alberta, Lac La Nonne; Ontario	*Physella gyrina*	Aythya collaris	KY207569, KY587397, KY587396	Gordy, M.A., et al., 2017, Parasitol. Res. 116(8): 2181‐98.
		Cercaria	Canada: Alberta, Gull Lake	*Physa gyrina*	Unidentified	MH369765	Present study
	*Australapatemon* sp. LIN5	Cercaria	Canada: Alberta, Buffalo Lake	*Stagnicola elodes*	Unidentified	KY207597	Gordy, M.A., et al., 2017, Parasitol. Res. 116(8): 2181‐98.
	*Australapatemon* sp. LIN6	Cercaria	Canada: Alberta, Pigeon Lake, Isle Lake	*Physa gyrina*	Unidentified	KY207613, KY207616	Gordy, M.A., et al., 2017, Parasitol. Res. 116(8): 2181‐98.
		Cercaria	Canada: Alberta, Isle Lake, Buffalo Lake, Lac La Nonne	*Physa gyrina*	Unidentified	MH369766, MH369767, MH369768, MH369769, MH369770	Present study
	*Australapatemon* sp. LIN8	Cercaria, Adult	Canada: Alberta, Isle Lake, Buffalo Lake; Ontario	*Physa gyrina*	Oxyura jamaicensis	KY207587, KY207622, HM385538, HM385537, HM385536	Gordy, M.A., et al., 2017, Parasitol. Res. 116(8): 2181‐98.
		Cercaria	Canada: Alberta, Isle Lake, Buffalo Lake, Gull Lake	*Physa gyrina*	Unidentified	MH369771, MH369772, MH369773, MH369774, MH369775, MH369776, MH369777	Present study
	*Australapatemon* sp. LIN9A	Cercaria, Adult	Canada: Alberta, Gull Lake, Isle Lake, Buffalo Lake; Ontario	*Stagnicola elodes*	Anas acuta	KY207550§, KY207557§, KY207558§, KY207582§, KY207596§, HM385534§	Gordy, M.A., et al., 2017, Parasitol. Res. 116(8): 2181‐98.
		Cercaria	Canada: Alberta, Lac La None, Gull Lake, Buffalo Lake, Isle Lake	*Stagnicola elodes, Lymnaea stagnalis (MGC176B)*	Unidentified	MH369779, MH369780, MH369781, MH369782, MH369783, MH369784, MH369785, MH369786, MH369787, MH369788, MH369789, MH369778	Present study
	*Australapatemon* sp. LIN9B	Cercaria	Canada: Alberta, Buffalo Lake	*Stagnicola elodes*	Unidentified	KY207583§	Gordy, M.A., et al., 2017, Parasitol. Res. 116(8): 2181‐98.
		Cercaria	Canada: Alberta, Buffalo Lake	*Stagnicola elodes*	Unidentified	MH369790, MH369791, MH369792	Present study
	*Australapatemon* sp. LIN10	Cercaria	Canada: Alberta, Gull Lake	*Stagnicola elodes*	Unidentified	MH369793	Present study
	*Cercariae douglasi*	Cercaria/Metacercaria	Lake Wabamun (114.35'W, 53.32'N)	*Physa gyrina*	Unidentified		Sankurathri, C.S. and Holmes, J.C., 1976, Can. J. Zool. 54:1742‐1753
	*Cotylurus cornutus*	Cercaria	Canada: Alberta, Gull Lake	*Stagnicola elodes*	Unidentified	KT831347§	Gordy, M.A., et al., 2016, Parasitol. Res. 115(10): 3867‐80.
		Cercaria	Canada: Alberta, Gull Lake, Isle Lake, Lac La Nonne	*Stagnicola elodes, Helisoma trivolvis (MGC205)*	Unidentified	MH369478, MH369480, MH369484, MH369485, MH369486, MH369487, MH369488, MH369489, MH369490, MH369491, MH369492, MH369493, MH369494, MH369495, MH369496, MH369497, MH369498, MH369500, MH369501, MH369502, MH369503, MH369504, MH369505, MH369509, MH369510, MH369511, MH369516, MH369532, MH369538, MH369539, MH369544, MH369557, MH369597, MH369601	Present study
	*Cotylurus erraticus*	Larval	Cold Lake (54.30'N, 110W)	Unidentified	White sucker, Whitefish, Cisco		Leong, T.S. and Holmes, J.C., 1981, J. Fish Biol. 18:693‐713
		Larval	Big Fish Lake, Caribou Lake, Eva Lake, Fleming Lake, Margaret Lake, Pitchimi Lake, Semo Lake, Sucker Lake, Wentzel Lake	Unidentified	Various fish species		Baldwin, R.E. and Goater, C.P., 2003, JP, 89(2):215‐225
		Adult	Beaverhill Lake (53.30'N, 112.30'W) and Miquelon Lake (53.15'N, 112.55'W)	Unidentified	Ring‐billed Gulls		Vermeer, K. 1969, Can. J. Zool. 47:267‐270
	*Cotylurus flabelliformis*	Cercaria	Canada: Alberta, Isle Lake	*Stagnicola elodes*	Unidentified	MH369519	Present study
	*Cotylurus hebraicus*	Adult	13 Lakes in Alberta	Unidentified	Lesser Scaup		Bush, A.O. and Holmes, J.C., 1986, Can. J. Zool. 64:132‐141
	*Cotylurus marcogliesei*	Cercaria	Canada: Alberta, Buffalo Lake, Gull Lake, Isle Lake, Lac La Nonne	*Stagnicola elodes*	Unidentified	MH369479, MH369481, MH369515, MH369530, MH369531, MH369553, MH369564	Present study
	*Cotylurus strigeoides*	Cercaria	Canada: Alberta, Buffalo Lake, Wabamun Lake, Isle Lake, Lac La Nonne	*Physa gyrina*	Unidentified	MH369517, MH369518, MH369525, MH369526, MH369527, MH369528, MH369529, MH369560, MH369571, MH369572, MH369574, MH369575, MH369577, MH369583, MH369584, MH369587, MH369588, MH369590, MH369595, MH369596, MH369599, MH369600	Present study
	*Cotylurus* sp. A	Cercaria	Canada: Alberta, Isle Lake	*Stagnicola elodes*	Unidentified	KT831371§	Gordy, M.A., et al., 2016, Parasitol. Res. 115(10): 3867‐80.
		Cercaria	Canada: Alberta, Isle Lake, Lac La Nonne, Wabamun	*Stagnicola elodes, Physa gyrina (MGC1962)*	Unidentified	MH369513, MH369520, MH369521, MH369522, MH369523, MH369524, MH369533, MH369537, MH369541, MH369542, MH369543, MH369545, MH369546, MH369547, MH369548, MH369549, MH369550, MH369551, MH369552, MH369554, MH369555, MH369556, MH369558, MH369559, MH369561, MH369562, MH369573, MH369578, MH369579, MH369580, MH369581, MH369582, MH369585, MH369589, MH369591, MH369594, MH369598, MH369602	Present study
	*Cotylurus* sp. B	Cercaria	Canada: Alberta, Isle Lake	*Physa gyrina*	Unidentified	MH369586	Present study
	*Cotylurus* sp. C	Cercaria	Canada: Alberta, Buffalo Lake	*Lymnaea stagnalis*	Unidentified	MH369563, MH369566, MH369576	Present study
	*Cotylurus* sp. D	Cercaria	Canada: Alberta, Buffalo Lake	*Physa gyrina*	Unidentified	MH369592	Present study
	*Cotylurus* sp. E	Cercaria	Canada: Alberta, Buffalo Lake, Gull Lake, Isle Lake, Lac La Nonne	*Stagnicola elodes*	Unidentified	MH369482, MH369483, MH369499, MH369506, MH369507, MH369508, MH369534, MH369535, MH369536, MH369540, MH369565, MH369593	Present study
	*Cotylurus* sp. F	Cercaria	Canada: Alberta, Buffalo Lake, Wabamun Lake	*Lymnaea stagnalis*	Unidentified	MH369512, MH369514, MH369567, MH369568, MH369569, MH369570	Present study
Unknown	*Choledocystus pennsylvaniensis*	Adult	Eastern Alberta	Unidentified	Western Chorus Frog		Goldberg, S.R., Bursey, C.R., and Wong, C., 2002, Northwest Science, 76(1)

§ Sequence updated in present study

## References

[ece34939-bib-0001] Adlard, R. , & O'Donoghue, P. (1998). Perspectives on the biodiversity of parasitic protozoa in Australia. International Journal for Parasitology, 28(6), 887–897. 9673868 10.1016/s0020-7519(98)00043-5

[ece34939-bib-0002] Adlard, R. D. , Miller, T. L. , & Smit, N. J. (2015). The butterfly effect: Parasite diversity, environment, and emerging disease in aquatic wildlife. Trends in Parasitology, 31, 160–166. 10.1016/J.PT.2014.11.001 25488771

[ece34939-bib-0003] Altschul, S. F. , Gish, W. , Miller, W. , Myers, E. W. , & Lipman, D. J. (1990). Basic local alignment search tool. Journal of Molecular Biology, 215, 403–410. 10.1016/S0022-2836(05)80360-2 2231712

[ece34939-bib-0004] Behrmann-Godel, J. (2013). Parasite identification, succession and infection pathways in perch fry (*Perca fluviatilis*): New insights through a combined morphological and genetic approach. Parasitol, 140(4), 509–520. 10.1017/S0031182012001989 23279837

[ece34939-bib-0005] Bergmame, L. , Huffman, J. , Cole, R. , Dayanandan, S. , Tkach, V. V. , & McLaughlin, J. D. (2011). *Sphaeridiotrema globulus* and *Sphaeridiotrema pseudoglobulus* (Digenea): Species differentiation based on mtDNA (Barcode) and partial LSU–rDNA sequences. Journal of Parasitology, 97(6), 1132–1136. 21671715 10.1645/GE-2370.1

[ece34939-bib-0006] Blankespoor, H. (1977). Notes on the biology of *Plagiorchis noblei* Park, 1936 (Trematoda: Plagiorchiidae). Proceedings of the Helminthological Society, 44(1), 44–50.

[ece34939-bib-0007] Blasco-Costa, I. , & Locke, S. A. (2017). Life history, systematics and evolution of the diplostomoidea poirier, 1886: Progress, promises and challenges emerging from molecular studies. Advances in Parasitology, 98, 167–225. 10.1016/bs.apar.2017.05.001 28942769

[ece34939-bib-0008] Blasco-Costa, I. , Poulin, R. , & Presswell, B. (2016). Species of *Apatemon* Szidat, 1928 and *Australapatemon* Sudarikov, 1959 (Trematoda: Strigeidae) from New Zealand: Linking and characterising life cycle stages with morphology and molecules. Parasitology Research, 115, 271–289. 10.1007/s00436-015-4744-0 26385467

[ece34939-bib-0009] Bray, R. A. , Cribb, T. H. , Littlewood, D. T. J. , & Waeschenbach, A. (2016). The molecular phylogeny of the digenean family Opecoelidae Ozaki, 1925 and the value of morphological characters, with the erection of a new subfamily. Folia Parasitol (Praha), 63, 1–11. 10.14411/fp.2016.013 27189270

[ece34939-bib-0010] Chibwana, F. D. , Blasco-Costa, I. , Georgieva, S. , Hosea, K. M. , Nkwengulila, G. , Scholz, T. , & Kostadinova, A. (2013). A first insight into the barcodes for African diplostomids (Digenea: Diplostomidae): Brain parasites in *Clarias gariepinus* (Siluriformes: Clariidae). Infection, Genetics and Evolution, 17, 62–70. 10.1016/j.meegid.2013.03.037 23542455

[ece34939-bib-0011] Cribb, T. H. , Bray, R. A. , Littlewood, D. T. J. , Pichelin, S. P. , Herniou, E. A. , & Warren, A. (2001). The Digenea In Littlewood D. T. J. (Ed.), Interrelationships of the Platyhelminthes (pp. 168–185). London: Taylor & Francis.

[ece34939-bib-0012] Detwiler, J. T. , Bos, D. H. , & Minchella, D. J. (2010). Revealing the secret lives of cryptic species: Examining the phylogenetic relationships of echinostome parasites in North America. Molecular Phylogenetics and Evolution, 55, 611–620. 10.1016/j.ympev.2010.01.004 20064622

[ece34939-bib-0013] Detwiler, J. T. , Zajac, A. M. , Minchella, D. J. , & Belden, L. K. (2012). Revealing cryptic parasite diversity in a definitive host: Echinostomes in muskrats. Journal of Parasitology, 98, 1148–1155. 10.1645/GE-3117.1 22694483

[ece34939-bib-0014] García-Varela, M. , Sereno-Uribe, A. L. , Pinacho-Pinacho, C. D. , Hernández-Cruz, E. , & Pérezponce de León, G. (2016). An integrative taxonomic study reveals a new species of *Tylodelphys* Diesing, 1950 (Digenea: Diplostomidae) in Central and Northern Mexico. Journal of Helminthology, 90(6), 668–679. 10.1017/S0022149X15000917 26508032

[ece34939-bib-0015] Georgieva, S. , Kostadinova, A. , & Skirnisson, K. (2012). The life-cycle of *Petasiger islandicus* Kostadinova & Skirnisson, 2007 (Digenea: Echinostomatidae) elucidated with the aid of molecular data. Systematic Parasitology, 82(3), 177–183. 10.1007/s11230-012-9354-y 22711507

[ece34939-bib-0016] Georgieva, S. , Selbach, C. , Faltýnková, A. , Soldánová, M. , Sures, B. , Skírnisson, K. , & Kostadinova, A. (2013a). New cryptic species of the ‘revolutum’ group of *Echinostoma* (Digenea: Echinostomatidae) revealed by molecular and morphological data. Parasit Vectors, 6, 64 10.1186/1756-3305-6-64 23497579 PMC3605289

[ece34939-bib-0017] Georgieva, S. , Soldánová, M. , Pérez-del-Olmo, A. , Dangel, D. R. , Sitko, J. , Sures, B. , & Kostadinova, A. (2013b). Molecular prospecting for European *Diplostomum* (Digenea: Diplostomidae) reveals cryptic diversity. International Journal for Parasitology, 43, 57–72. 10.1016/j.ijpara.2012.10.019 23201234

[ece34939-bib-0018] Georgieva, S , Faltýnková, A , Brown, R , Blasco-Costa, I , Soldánová, M , Sitko, J , & Kostadniova, A . *Echinostoma “revolutum”* (Digenea: Echinostomatidae) species complex revisited: Species delimitation based on novel molecular and morphological data gathered in Europe. Parasit Vectors 2014;7:520 10.1186/s13071-014-0520-8 25430038 PMC4258292

[ece34939-bib-0019] Gibson, DI , Bray, RA , & Harris, EA . Host-Parasite Database of the Natural History Museum, London 2005.

[ece34939-bib-0020] Gordy, M. A. , Cobb, T. P. , & Hanington, P. C. (2018). Swimmer's itch in Canada: A look at the past and a survey of the present to plan for the future. Environmental Health, 17, 73 10.1186/s12940-018-0417-7 30359259 PMC6203143

[ece34939-bib-0021] Gordy, M. A. , Kish, L. , Tarrabain, M. , & Hanington, P. C. (2016). A comprehensive survey of larval digenean trematodes and their snail hosts in central Alberta Canada. Parasitology Research, 115(10), 3867–3880. 10.1007/s00436-016-5152-9 27245072

[ece34939-bib-0022] Gordy, MA , Locke, SA , Rawlings, TA , Lapierre, AR , & Hanington, PC . Molecular and morphological evidence for nine species in North American *Australapatemon* (Sudarikov, 1959): A phylogeny expansion with description of the zygocercous *Australapatemon mclaughlini* n. sp. Parasitology Research 2017;116:2181–2198. 10.1007/s00436-017-5523-x 28623502

[ece34939-bib-0023] Graczyk, T. K. , & Shiff, C. J. (1993a). Excystment in vitro of *Cotylurus cornutus* (Trematoda: Strigeidae) Metacercariae. Journal of Parasitology, 79, 448 10.2307/3283586 8501606

[ece34939-bib-0024] Graczyk, T. K. , & Shiff, C. J. (1993b). Experimental infection of domestic ducks and rodents by *Notocotylus attenuatus* (Trematoda: Notocotylidae). Journal of Wildlife Diseases, 29, 434–439. 10.7589/0090-3558-29.3.434 8355345

[ece34939-bib-0025] Guindon, S , Dufayard, JF , Lefort, V , Anisimova, M , Hordijk, W , & Gascuel, O . New algorithms and methods to estimate maximum-likelihood phylogenies: Assessing the performance of PhyML 3.0. Systematic Biology 2010;59:307–321. 10.1093/sysbio/syq010 20525638

[ece34939-bib-0026] Hernández-Cruz, E , Hernández-Orts, JS , Sereno-Uribe, AL , dePérez-Ponce León, G , & García-Varela, M . Multilocus phylogenetic analysis and morphological data reveal a new species composition of the genus *Drepanocephalus* Dietz, 1909 (Digenea: Echinostomatidae), parasites of fish-eating birds in the Americas. Journal of Helminthology 2018;92:572–595. 10.1017/s0022149x17000815 28974279

[ece34939-bib-0027] Hernández-Mena, D. I. , García-Prieto, L. , & García-Varela, M. (2014). Morphological and molecular differentiation of parastrigea (Trematoda: Strigeidae) from Mexico, with the description of a new species. Parasitology International, 63(2), 315–323. 10.1016/j.parint.2013.11.012 24309555

[ece34939-bib-0028] Hernández-Mena, DI , García-Varela, M , & dePérez-Ponce León, G . Filling the gaps in the classification of the Digenea Carus, 1863: Systematic position of the Proterodiplostomidae Dubois, 1936 within the superfamily Diplostomoidea Poirier, 1886, inferred from nuclear and mitochondrial DNA sequences. Systematic Parasitology 2017; 94:833 10.1007/s11230-017-9745-1 28822036

[ece34939-bib-0029] Herrmann, K. K. , Poulin, R. , Keeney, D. B. , & Blasco-Costa, I. (2014). Genetic structure in a progenetic trematode: Signs of cryptic species with contrasting reproductive strategies. International Journal for Parasitology, 44, 811–818. 10.1016/j.ijpara.2014.06.006 25058509

[ece34939-bib-0030] Hoberg, EP , Galbreath, KE , Cook, JA , Kutz, SJ , & Polley, L . Northern Host-Parasite Assemblages . History and biogeography on the borderlands of episodic climate and environmental transition. vol. 79. Elsevier; 2012; 79:1–97. 10.1016/b978-0-12-398457-9.00001-9 22726642

[ece34939-bib-0031] Huelsenbeck, J. P. , & Ronquist, F. (2001). MrBayes: Bayesian inference of phylogeny. Bioinformatics, 17, 754–755. 10.1093/bioinformatics/17.8.754 11524383

[ece34939-bib-0032] Jensen, K. , & Bullard, S. A. (2010). Characterization of a diversity of tetraphyllidean and rhinebothriidean cestode larval types, with comments on host associations and life-cycles. International Journal for Parasitology, 40, 889–910. 10.1016/J.IJPARA.2009.11.015 20026125

[ece34939-bib-0033] Kearse, M. , Moir, R. , Wilson, A. , Stones-Havas, S. , Cheung, M. , Sturrock, S. , … Drummond, A. (2012). Geneious Basic: An integrated and extendable desktop software platform for the organization and analysis of sequence data. Bioinformatics, 28, 1647–1649. 10.1093/bioinformatics/bts199 22543367 PMC3371832

[ece34939-bib-0034] Keas, B. E. , & Blankespoor, H. D. (1997). The prevalence of Cercariae from *Stagnicola emarginata* (Lymnaeidae) over 50 years in Northern Michigan. Journal of Parasitology, 83, 536 10.2307/3284427 9194844

[ece34939-bib-0035] Kostadinova, A. , & Herniou, E. (2003). Phylogenetic relationships of *Echinostoma* Rudolphi, 1809 (Digenea: Echinostomatidae) and related genera re-assessed via DNA and morphological analyses. Systematic Parasitology, 54(3), 159–176. 12652069 10.1023/a:1022681123340

[ece34939-bib-0036] Kudlai, O. , Kostadinova, A. , Pulis, E. E. , & Tkach, V. V. (2015). A new species of *Drepanocephalus* Dietz, 1909 (Digenea: Echinostomatidae) from the double-crested cormorant *Phalacrocorax auritus* (Lesson) (Aves: Phalacrocoracidae) in North America. Systematic Parasitology, 90(3), 221–230. 10.1007/s11230-015-9550-7 25693456

[ece34939-bib-0037] Kuhn, J. A. , Kristoffersen, R. , Knudsen, R. , Jakobsen, J. , Marcogliese, D. J. , Locke, S. A. , & Amundsen, P.-A. (2015). Parasite communities of two three-spined stickleback populations in subarctic norway—effects of a small spatial-scale host introduction. Parasitology Research, 114(4), 1327-1339. https://doi:10.1007/s00436-015-4309-2. 25630694 10.1007/s00436-015-4309-2

[ece34939-bib-0038] Kumar, S , Stecher, G , & Tamura, K . MEGA7: Molecular Evolutionary Genetics Analysis version 7.0 for bigger datasets. Molecular Biology and Evolution 2016;33:msw054 10.1093/molbev/msw054 PMC8210823 27004904

[ece34939-bib-0039] Liu, Z-X , Zhang, Y , Liu, Y‐T , Chang, Q‐C , Su, X , Fu, X , & Wang, C-R . Complete mitochondrial genome of *Echinostoma hortense* (Digenea: Echinostomatidae). Korean Journal of Parasitology 2016;54:173–179. 10.3347/kjp.2016.54.2.173 27180575 PMC4870973

[ece34939-bib-0040] Locke, SA , McLaughlin, JD , Dayanandan, S , & Marcogliese, D. J. Diversity and specificity in *Diplostomum* spp. metacercariae in freshwater fishes revealed by cytochrome *c* oxidase I and internal transcribed spacer sequences. International Journal for Parasitology 2010a;40:333–343. 10.1016/j.ijpara.2009.08.012 19737570

[ece34939-bib-0041] Locke, S. A. , McLaughlin, J. D. , & Marcogliese, D. J. (2010b). DNA barcodes show cryptic diversity and a potential physiological basis for host specificity among Diplostomoidea (Platyhelminthes: Digenea) parasitizing freshwater. Molecular Ecology, 19, 2813–2827. 20561195 10.1111/j.1365-294X.2010.04713.x

[ece34939-bib-0042] Locke, S. A. , McLaughlin, J. D. , Lapierre, A. R. , Johnson, P. T. J. , & Marcogliese, D. J. (2011). Linking larvae and adults of *Apharyngostrigea cornu*, *Hysteromorpha triloba*, and *Alaria mustelae* (Diplostomoidea: Digenea) using molecular data. Journal of Parasitology, 97(5), 846–851. 10.1645/GE-2775.1 21510747

[ece34939-bib-0043] Locke, S. A. , Al-Nasiri, F. S. , Caffara, M. , Drago, F. , Kalbe, M. , Lapierre, A. R. , McLaughlin, J. D. , … Marcogliese, D. J. (2015). Diversity, specificity and speciation in larval diplostomidae (Platyhelminthes: Digenea) in the eyes of freshwater fish, as revealed by DNA barcodes. International Journal for Parasitology, 45(13), 841–855. 10.1016/j.ijpara.2015.07.001 26276524

[ece34939-bib-0044] Locke, S. A. , Van Dam, A. , Caffara, M. , Pinto, H. A. , López-Hernández, D. , & Blanar, C. A. (2018). Validity of the diplostomoidea and diplostomida (Digenea, Platyhelminthes) upheld in phylogenomic analysis. International Journal for Parasitology, 48(13), 1043–1059. 10.1016/J.IJPARA.2018.07.001 30347194

[ece34939-bib-0045] Mathison, B. A. , & Pritt, B. S. (2014). Laboratory identification of arthropod ectoparasites. Clinical Microbiology Reviews, 27, 48–67. 10.1128/CMR.00008-13 24396136 PMC3910909

[ece34939-bib-0046] Miura, O. , Kuris, A. , & Torchin, M. (2005). Molecular-genetic analyses reveal cryptic species of trematodes in the intertidal gastropod, *Batillaria cumingi* (Crosse). International Journal for Parasitology, 35, 793–801. 15925598 10.1016/j.ijpara.2005.02.014

[ece34939-bib-0047] Mollaret, I. , Jamieson, B. G. , Adlard, R. D. , Hugall, A. , Lecointre, G. , Chombard, C. , & Justine, J. L. (1997). Phylogenetic analysis of the Monogenea and their relationships with Digenea and Eucestoda inferred from 28S rDNA sequences. Molecular and Biochemical Parasitology, 90, 433–438. 9476791 10.1016/s0166-6851(97)00176-x

[ece34939-bib-0048] Morgan, J. A. T. , & Blair, D. (1998). Mitochondrial ND1 gene sequences used to identify echinostome isolates from Australia and New Zealand. International Journal for Parasitology, 28, 493–502. 10.1016/S0020-7519(97)00204-X 9559367

[ece34939-bib-0049] Moszczynska, A , Locke, SA , McLaughlin, JD , Marcogliese, DJ , & Crease, TJ . Development of primers for the mitochondrial cytochrome *c* oxidase I gene in digenetic trematodes (Platyhelminthes) illustrates the challenge of barcoding parasitic helminths. Molecular Ecology Resources 2009;9 Suppl s1:75–82. 10.1111/j.1755-0998.2009.02634.x 21564967

[ece34939-bib-0050] Nadler, S. , & DE León, G. (2011). Integrating molecular and morphological approaches for characterizing parasite cryptic species: Implications for parasitology. Parasitology, 138, 1688–1709. 21281559 10.1017/S003118201000168X

[ece34939-bib-0051] Nagataki, M , Tantrawatpan, C , Agatsuma, T , Sugiura, T , Duenngai, K , Sithithaworn, P , … Saijuntha, W . Mitochondrial DNA sequences of 37 collar-spined echinostomes (Digenea: Echinostomatidae) in Thailand and Lao PDR reveals presence of two species: *Echinostoma revolutum* and *E. miyagawai* . Infect Genet Evol, 2015; 35: 56–62. 26205690 10.1016/j.meegid.2015.07.022

[ece34939-bib-0052] O'Hear, M. , Pote, L. , Yost, M. , Doffitt, C. , King, T. , & Panuska, C. (2014). Morphologic and molecular identifications of digenetic trematodes in double-crested cormorants (*Phalacrocorax auritus*) from the Mississippi Delta. USA. J Wildlife Dis, 50(1), 42–49. 10.7589/2012-10-249 24171572

[ece34939-bib-0053] Oksanen, J , Blanchet, F. G. , Friendly, M , Kindt, R , Legendre, P , McGlinn, D , & Wagner, H . Vegan: Community ecology package 2018.

[ece34939-bib-0054] Otachi, E. O. , Locke, S. A. , Jirsa, F , Fellner-Frank, C , & Marcogliese, DJ . Morphometric and molecular analyses of *Tylodelphys* sp. Metacercariae (Digenea: Diplostomidae) from the vitreous humour of four fish species from lake Naivasha, Kenya. Journal of Helminthology 2015; 89(4): 404–414. 10.1017/s0022149x14000170 24690126

[ece34939-bib-0055] Pérez-Ponce de León, G , & Poulin, R . An updated look at the uneven distribution of cryptic diversity among parasitic helminths. Journal of Helminthology 2018;92:197–202. 10.1017/s0022149x17000189 28260533

[ece34939-bib-0056] Pinto, H. A. , Griffin, M. J. , Quiniou, S. M. , Ware, C , & Melo, A. L. . *Biomphalaria straminea* (Mollusca: Planorbidae) as an intermediate host of *Drepanocephalus* spp. (Trematoda: Echinostomatidae) in Brazil: A morphological and molecular study. Parasitology Research 2016; 115(1): 51–62. 10.1007/s00436-015-4469-0 25982569

[ece34939-bib-0057] Poulin, R. (2014). Parasite biodiversity revisited: Frontiers and constraints. International Journal for Parasitology, 44, 581–589. 10.1016/J.IJPARA.2014.02.003 24607559

[ece34939-bib-0058] Poulin, R. , Besson, A. A. , Morin, M. B. , & Randhawa, H. S. (2016). Missing links: Testing the completeness of host-parasite checklists. Parasitology, 143, 114–122. 10.1017/S0031182015001559 26549369

[ece34939-bib-0059] Poulin, R. , & Jorge, F. (2018). The geography of parasite discovery across taxa and over time. Parasitology, 1–8. 10.1017/S003118201800118X 30012225

[ece34939-bib-0060] Poulin, R , & Morand, S . Parasite Biodiversity. Smithsonian Books; 2004 10.1645/ge-702r.1

[ece34939-bib-0061] Puillandre, N. , Lambert, A. , Brouillet, S. , & Achaz, G. (2012). ABGD, automatic barcode gap discovery for primary species delimitation. Molecular Ecology, 21, 1864–1877. 10.1111/j.1365-294X.2011.05239.x 21883587

[ece34939-bib-0062] R Core Team . R: A language and environment for statistical computing 2017.

[ece34939-bib-0063] Roeber, F. , Jex, A. R. , & Gasser, R. B. (2013). Advances in the diagnosis of key gastrointestinal nematode infections of livestock, with an emphasis on small ruminants. Biotechnology Advances, 31, 1135–1152. 10.1016/J.BIOTECHADV.2013.01.008 23376340 PMC7126997

[ece34939-bib-0064] Rosser, T. G. , Baumgartner, W. A. , Alberson, N. R. , Woodyard, E. T. , Reichley, S. R. , Wise, D. J. , … Griffin, M. J. . *Austrodiplostomum* sp., *Bolbophorus* sp.(Digenea: Diplostomidae), and Clinostomum marginatum (Digenea: Clinostomidae) metacercariae in inland silverside *Menidia beryllina* from catfish aquaculture ponds, with notes on the infectivity of *Austrodiplostomum* sp. cercariae in channel catfish Ictalurus punctatus. Parasitology Research 2016; 115(11): 4365–4378. 27539726 10.1007/s00436-016-5222-z

[ece34939-bib-0065] Schell, S. C. (1985). Handbook of the trematodes of North America north of Mexico. Moscow (Idaho): University Press Idaho.

[ece34939-bib-0066] Schwelm, J. , Soldánová, M. , Vyhlídalová, T. , Sures, B. , & Selbach, C. (2018). Small but diverse: Larval trematode communities in the small freshwater planorbids *Gyraulus albus* and *Segmentina nitida* (Gastropoda: Pulmonata) from the Ruhr River, Germany. Parasitology Research, 117, 241–255. 10.1007/s00436-017-5699-0 29222665

[ece34939-bib-0067] Selbach, C. , Soldánová, M. , Georgieva, S. , Kostadinova, A. , Kalbe, M. , & Sures, B. (2014). Morphological and molecular data for larval stages of four species of *Petasiger* Dietz, 1909 (Digenea: Echinostomatidae) with an updated key to the known cercariae from the palaearctic. Systematic Parasitology, 89(2), 153–166. 10.1007/s11230-014-9513-4 25204601

[ece34939-bib-0068] Selbach, C , Soldánová, M , Georgieva, S , Kostadinova, A , & Sures, B . Integrative taxonomic approach to the cryptic diversity of *Diplostomum* spp. in lymnaeid snails from Europe with a focus on the ‘*Diplostomum mergi*'species complex.” Parasit Vectors 2015; 8(1): 300. 26036245 10.1186/s13071-015-0904-4 PMC4476078

[ece34939-bib-0069] Snyder, S. D. , & Tkach, V. V. (2001). Phylogenetic and biogeographical relationships among some holarctic frog lung flukes (Digenea: Haematoloechidae). Journal of Parasitology, 87, 1433 10.2307/3285315 11780834

[ece34939-bib-0070] Soldánová, M. , Georgieva, S. , Roháčová, J. , Knudsen, R. , Kuhn, J. A. , Henriksen, E. H. , … Kostadinova, A. (2017). Molecular analyses reveal high species diversity of trematodes in a sub-Arctic lake. International Journal for Parasitology, 47, 327–345. 10.1016/j.ijpara.2016.12.008 28315362

[ece34939-bib-0071] Stoyanov, B. , Georgieva, S. , Pankov, P. , Kudlai, O. , Kostadinova, A. , & Georgiev, B. B. (2017). Morphology and molecules reveal the alien *Posthodiplostomum centrarchi* Hoffman, 1958 as the third species of *Posthodiplostomum* Dubois, 1936 (Digenea: Diplostomidae) in Europe. Systematic Parasitology, 94, 1–20. 10.1007/s11230-016-9680-6 28062983

[ece34939-bib-0072] Tkach, V. V. , Kudlai, O. , & Kostadinova, A. (2016). Molecular phylogeny and systematics of the Echinostomatoidea Looss, 1899 (Platyhelminthes: Digenea). International Journal for Parasitology, 46, 171–185. 10.1016/J.IJPARA.2015.11.001 26699402

[ece34939-bib-0073] Van Steenkiste, N. , Locke, S. A. , Castelin, M. , Marcogliese, D. J. , & Abbott, C. L. (2014). New primers for DNA barcoding of digeneans and cestodes (Platyhelminthes). Molecular Ecology Resources, 15, 945–952. 10.1111/1755-0998.12358 25490869

[ece34939-bib-0074] Vaughan, JA , Tkach, VV , & Greiman, SE . Neorickettsial endosymbionts of the digenea: Diversity, transmission and distribution. vol. 79. Elsevier; 2012 10.1016/b978-0-12-398457-9.00003-2 22726644

[ece34939-bib-0075] Vilas, R. , Criscione, C. , & Blouin, M. (2005). A comparison between mitochondrial DNA and the ribosomal internal transcribed regions in prospecting for cryptic species of platyhelminth parasites. Parasitology, 131, 1–8. 16336737 10.1017/S0031182005008437

[ece34939-bib-0076] Wickham, H , Francois, R , Henry, L , & Müller, K . dplyr: A grammar of data manipulation. R package 2017

